# The trends in death of primary liver cancer caused by specific etiologies worldwide: results from the Global Burden of Disease Study 2019 and implications for liver cancer management

**DOI:** 10.1186/s12885-023-11038-3

**Published:** 2023-06-28

**Authors:** Yongzhi Li, Zejin Ou, Danfeng Yu, Huan He, Liting Zheng, Jiaqi Chen, Caiyun Chen, Hushen Xiong, Qing Chen

**Affiliations:** 1 Guangdong Provincial Key Laboratory of Tropical Disease Research, Department of Epidemiology, School of Public Health, Southern Medical University, 1838 Guangzhou North Road, Guangzhou, 510515 China; 2 Key Laboratory of Occupational Environment and Health, Guangzhou Twelfth People’s Hospital, Guangzhou, China; 3 Department of MICU, Guangdong Women and Children Hospital, Guangzhou, China

**Keywords:** Liver cancer, Global burden of disease, Age-standardized rate, Estimated annual percentage changes, Death

## Abstract

**Background:**

Over past decades, epidemiological patterns of liver cancer (LC) have changed dramatically. The Global Burden of Disease (GBD) study provides an opportunity for tracking the progress in cancer control with its annual updated reports at national, regional and global level, which can facilitate the health decision-making and the allocation of health resources. Therefore, we aim to estimate the global, regional and national trends of death caused by liver cancer due to specific etiologies and attributable risks from 1990 to 2019.

**Materials and methods:**

Data was collected from the GBD study 2019. Estimated annual percentage changes (EAPC) were used to quantify the trends of age-standardized death rate (ASDR). We applied a linear regression for the calculation of estimated annual percentage change in ASDR.

**Results:**

From 1990 to 2019, the ASDR of liver cancer decreased globally (EAPC =  − 2.23, 95% confidence interval [CI]: − 2.61 to − 1.84). Meanwhile, declining trends were observed in both sexes, socio-demographic index (SDI) areas, and geographies, particularly East Asia (EAPC =  − 4.98, 95% CI: − 5.73 to − 4.22). The ASDR for each of the four major etiologies fell globally, while liver cancer caused by hepatitis B had the largest drop (EPAC =  − 3.46, 95% CI: − 4.01 to − 2.89). China has had dramatic decreases in death rates on a national scale, particularly when it comes to the hepatitis B etiology (EAPC =  − 5.17, 95% CI: − 5.96 to − 4.37). However, certain nations, such as Armenia and Uzbekistan, saw a rise in liver cancer mortality. Controlling smoking, alcohol, and drug use contributed to a drop in LC-related mortality in the majority of socio-demographic index areas. Nevertheless, the excessive body mass index (BMI) was portrayed as the underlying cause for LC fatalities.

**Conclusion:**

From 1990 to 2019, there was a worldwide decrease in deaths caused by liver cancer and its underlying causes. However, rising tendencies have been observed in low-resource regions and countries. The trends in drug use- and high BMI-related death from liver cancer and its underlying etiologies were concerning. The findings indicated that efforts should be increased to prevent liver cancer deaths through improved etiology control and risk management.

**Supplementary Information:**

The online version contains supplementary material available at 10.1186/s12885-023-11038-3.

## Background

Liver cancer was the fourth leading cause of neoplasm death after lung, colorectal, and stomach cancer in 2017 [1]. Due to exposure to risk factors, there are obvious differences in the burden of liver cancer by sex and geographic region [2]. Major risk factors include infections (hepatitis B virus [HBV], hepatitis C virus [HCV], liver flukes in endemic areas), behavioral factors (alcohol, tobacco), metabolic factors (excess body fatness), and aflatoxins [3]. Because of the time lag between exposure to risk factors and the development of liver cancer, even the best-case scenarios of these preventative methods are unlikely to appreciably lower the number of patients with liver cancer that healthcare systems must treat in the near future.

Over past decades, epidemiological patterns of liver cancer have changed dramatically [4]. To overcome deficient epidemiological data, the Global Burden of Disease (GBD) scientists created statistical methods that provided the most accurate and comparable estimates of the worldwide burden of 29 cancer groups across in 195 countries [5]. However, no updated global studies on liver cancer have been published since the 2017 estimates. To provide comparable, comprehensive, and up-to-date details, this study presents estimates of numbers and age-standardized rates of death (ASDRs), and estimated annual percentage change (EAPC) for liver cancer in 204 countries and territories from 1990 to 2019.

ASDR considerably decreased in regions with high liver cancer burden such as East Asia and Western sub-Saharan Africa from 1990 to 2015, while increased more than doubled in Philippines, Moldova, and Guatemala [6]. In addition, survival of liver cancer only increased by 5–10% in most countries during the period 1995–2014, particularly in some developed countries [7]. Changing survival patterns of liver cancer were influenced by many factors, including vaccine coverage, local medical resource, metabolic syndrome, and lifestyles [8–10]. Prevention and treatment of hepatitis B contributed for the majority of decrease in death caused by liver cancer [11]. Furthermore, the detection of liver cancer at an early stage had markedly improved the 5-year survival [12]. Nevertheless, the high prevalence of alcohol use, drug use, and obesity were growing risks in the expansion of liver cancer death in recent years [13–15]. All these risk factors were preventable, and dynamically varied in different countries over time, emphasizing the necessity of tracking the temporal trends of burden caused by liver cancer. Consequently, the analysis of liver cancer as part of the GBD 2019 study serves two main objectives: first, to provide detailed information on liver cancer etiologies and their trends over time, without which targeted prevention strategies are impossible to design and to evaluate; and second, to encourage strategic investments in research and clinical resources.

Trends of death caused by liver cancer underlying specific etiologies and attributable risks were demonstrated using the latest version of Global Burden of Disease (GBD) study, providing an important data to public health strategies. The aim of this study was to estimate the global, regional and national trends in liver cancer deaths from 1990 to 2019 due to specific etiological and attributable risks. Progress in controlling liver cancer is tracked through annual updates to assist in health decision-making and allocation of health resources.

## Methods

### Ethics statement

This research was approved by the Ethics Committee of Southern Medical University (Guangzhou, China). The methods were carried out following the Declaration of Helsinki and its later amendments or comparable ethical standards.

### Data sources

Data on liver cancer cases and mortality from GBD study 2019 by age, sex, region, country, etiology and attributable risks were obtained using the Global Health Data Exchange (GHDx) query tool (http://ghdx.healthdata.org/gbd-results-tool). Data from a total of 204 countries and territories divided into 21 regions were available. The socio-demographic index (SDI) regions were then categorized into five levels, including low, low-middle, middle, high-middle, and high. Data on the Human Development Index (HDI) was also acquired from the United Nations Development Program (http://hdr.undp.org/en/data) in this study [16].

In order to assess the burden of liver cancer, data from all accessible sources, including published studies, surveys, censuses, surveillance systems, vital statistics, and other sources of health-related data, were gathered. The International Classification of Diseases (ICD) version 9 (155–155.963) and version 10 (C22.0–22.9) codes for liver cancer were applied [17].

For each study the proportions of liver cancer due to the four etiologies (HBV, HCV, alcohol consumption, and non-alcoholic steatohepatitis) were calculated. Remaining risk factors and exposures related to underlying etiologies were included under a combined “attributable risks” group, which consist of smoking, alcohol use, drug use, high fasting plasma glucose and high body-mass index (BMI) with the data of 46,000 empirical data points on the basis of cohort studies and randomized controlled trials.

### Statistical analysis

We used the age-standardized death rate (ASDR) and estimated annual percentage change (EAPC) to quantify the liver cancer death trends [18]. Age-standardization is necessary and representative when comparing in several populations with different age structures or for the same population over time.

The ASDR (per 100,000 population) in accordance with the direct method is calculated by summing up the products of the age-specific rates (*a*_*i*_ represents the age-specific rate in the *i*^*th*^ age group) and the number of persons (or weight) (*w*_*i*_) in the corresponding *i*^*th*^ age group from among the selected reference standard population, then dividing the sum of standard population weights, i.e.,

$$\mathrm{ASDR}=\frac{{\sum }_{i=1}^{A}{a}_{i}{w}_{i}}{{\sum }_{i=1}^{A}{w}_{i}}\times \mathrm{100,000}$$

More importantly, the ASDR trends can serve as a good surrogate for shifting patterns of disease within a population, as well as clues to the changing risk factors. Consequently, we can assess the effectiveness of current prevention strategies and establish more targeted ones, if needed, based on the analysis in the ASDR [19].

Estimated annual percentage change (EAPC) is a reliable method and widely used measure for describing the magnitude of the trends in ASDR [17, 20]. A regression line was fitted to the natural logarithm of the rates. The EAPC and its 95% confidence interval (CI) were calculated using the linear regression model, i.e.,

$$y =a+\beta x+\varepsilon ,$$

$$\mathrm{EAPC}=100\times \left(\mathrm{exp}\left(\upbeta \right)-1\right)$$

where *y* = ln (ASDR) and *x* = calendar year. An increasing trend was determined if both EAPC and its 95% CI were > 0. Conversely, a decreasing trend was determined if both EAPC and its 95% CI were < 0. Other outcomes were considered to be “stable” over time. Additionally, in order to explore the impact factors of EAPC, the associations between EAPC and ASDR in 1990, and between EAPC and HDI in 2019 were explored at the national level, respectively. Data were analyzed using R v3.6.2 (R Institute for Statistical Computing, Vienna, Austria). A *P* value of less than 0.05 was deemed to be statistically significant.

## Results

### Global burden and age patterns of liver cancer

**Fig. 1:**
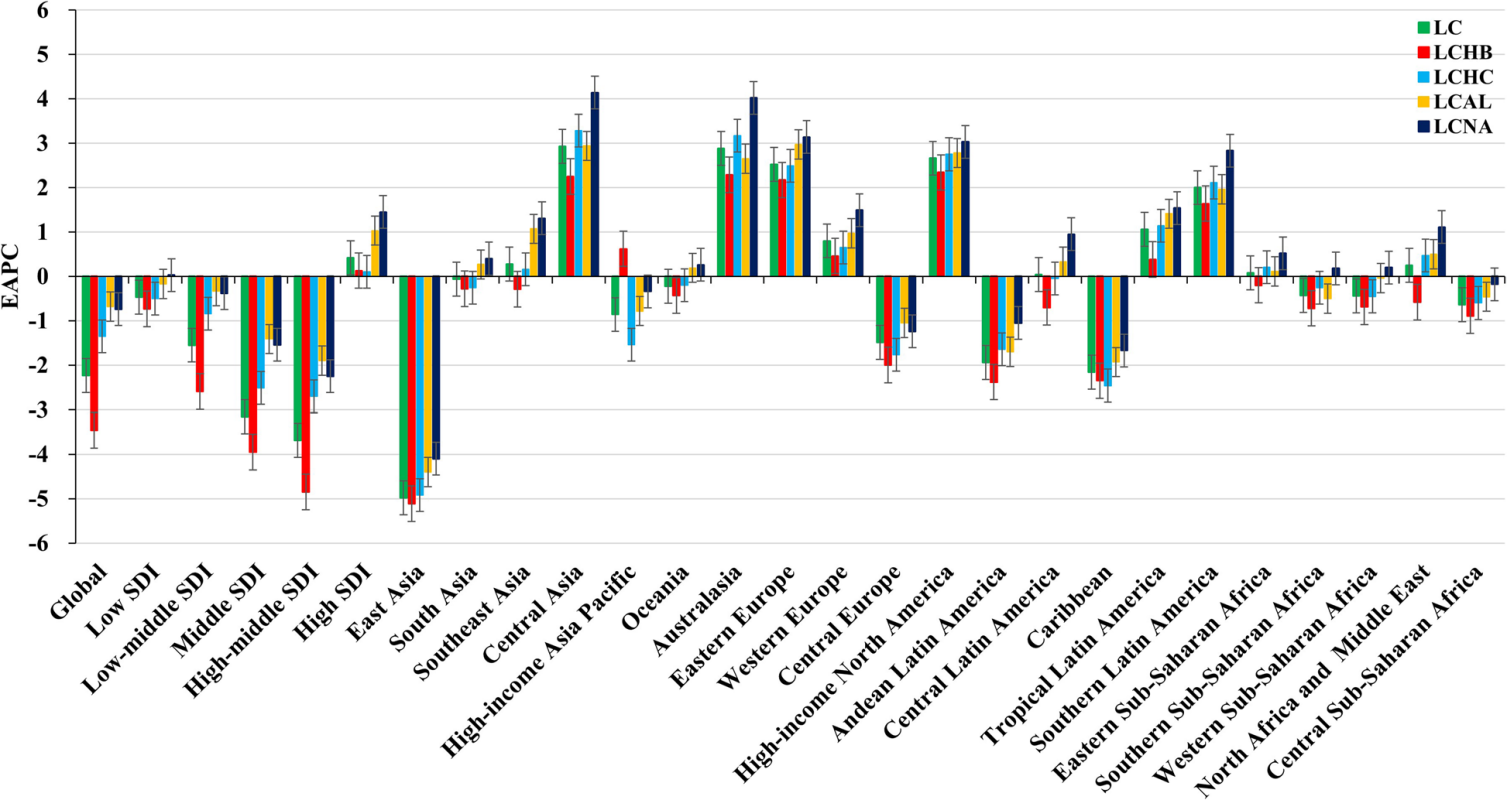
Trends of death caused by liver cancer and its four etiologies globally, and in SDI areas and geographic regions from 1990 to 2019. LC, liver cancer; LCHB, liver cancer due to hepatitis B; LCHC, liver cancer due to hepatitis C; LCAL, liver cancer due to alcohol consumption; LCNA, liver cancer due to non-alcoholic steatohepatitis; EAPC, Estimated annual percentage change; SDI, sociodemographic index

**Fig. 2:**
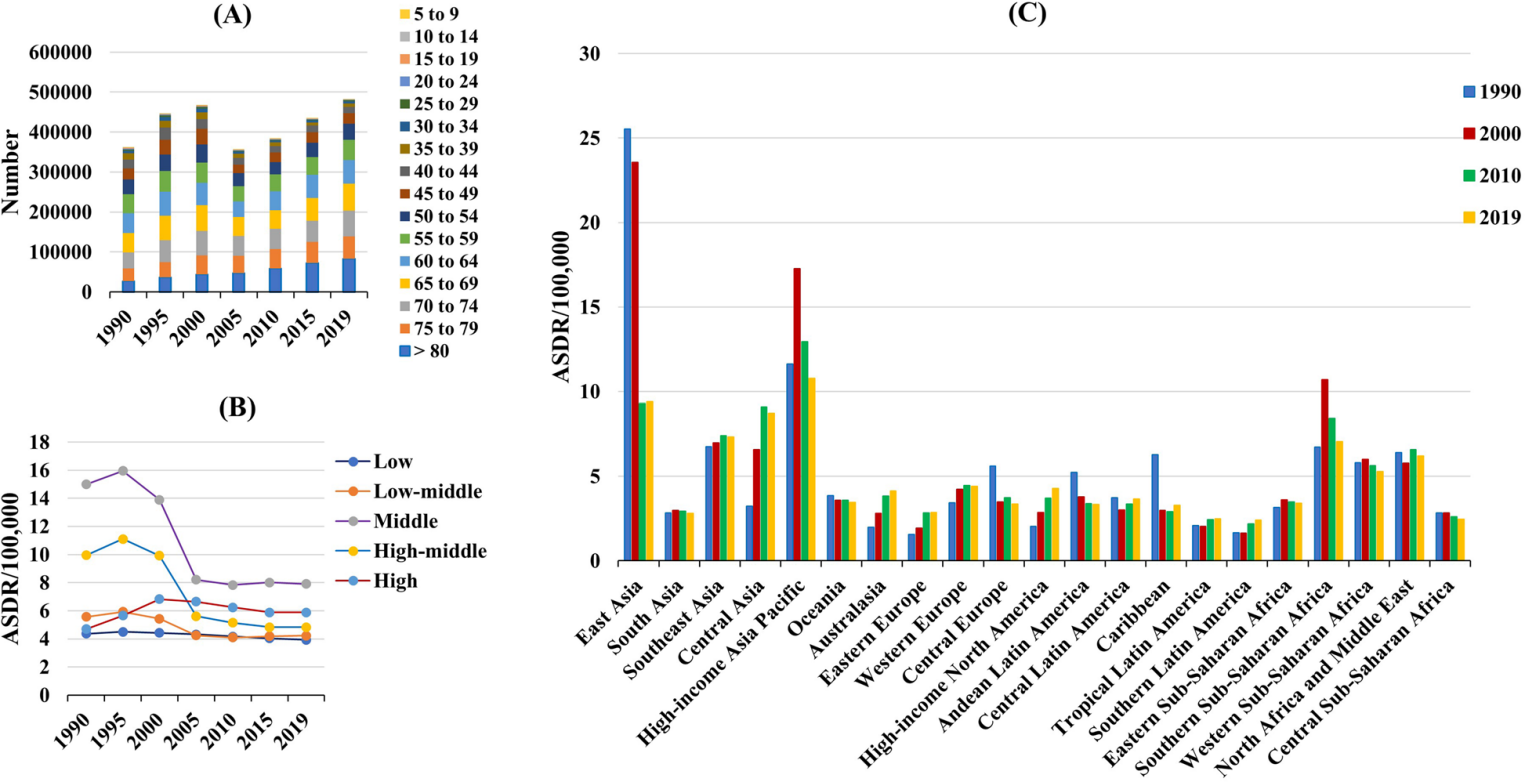
The distribution of number and ASDR caused by LC in age groups, SDI areas, and geographic regions from 1990 to 2019. **A** The death number of liver cancer in age groups; **B** and **C** The ASDR of LC in SDI areas and geographical regions, respectively. ASDR, age-standardized death rate; SDI, sociodemographic index

Globally, the liver cancer (LC) caused 484.58 × 10^3^ (95% uncertainty interval [UI]: 444.09 × 10^3^ to 525.80 × 10^3^) death worldwide in 2019, with an increase of 32.68% since 1990 (Table 1). The overall age-standardized death rate (ASDR) decreased by an average 2.23% per year from 1990 to 2019 (EAPC =  − 2.23, 95% CI: − 2.61 to − 1.84) (Table 1, Fig. 1). Increasing changes of death number occurred in those aged over 50 years, particularly in the group of > 80 years (202.62%) (Table 2, Fig. 2A).

**Table 1:** The number and age-standardized rate of death due to liver cancer in global, sexes, SDI areas and geographic regions in 1990 and 2019, and the percentage change in number and EAPCs from 1990 to 2019

	**1990**		**2019**		**1990–2019**	
**Characteristics**	Number No. × 10^3^ (95% UI)	ASDR (/100,000) No. (95% UI)	Number No. × 10^3^ (95% UI)	ASDR (/100,000) No. (95% UI)	Change in number (%)	EAPC No. (95% CI)
**Overall**	365.22 (329.97–405.77)	8.93 (8.09–9.90)	484.58 (444.09–525.80)	5.95 (5.44–6.44)	32.68	− 2.23 (− 2.61− − 1.84)
**Sex**						
Male	251.00 (218.33–287.14)	12.90 (11.30–14.67)	333.67 (299.58–368.33)	8.73 (7.88–9.60)	32.94	− 2.26 (− 2.68− − 1.84)
Female	114.22 (100.16–130.72)	5.33 (4.67–6.09)	150.90 (134.12–167.01)	3.46 (3.08–3.83)	32.12	− 2.10 (− 2.39− − 1.80)
**SDI**						
Low	10.56 (9.24–11.90)	4.37 (3.80–4.97)	20.76 (18.22–23.33)	3.93 (3.49–4.38)	96.50	− 0.47 (− 0.53− − 0.40)
Low-middle	34.78 (31.45–38.18)	5.57 (5.05–6.13)	57.24 (52.13–63.45)	4.23 (3.86–4.68)	64.58	− 1.55 (− 1.84− − 1.25)
Middle	163.81 (142.72–189.46)	15.00 (13.15–17.26)	196.96 (172.83–223.21)	7.92 (6.97–8.93)	20.24	− 3.16 (− 3.71− − 2.61)
High-middle	107.83 (94.48–122.72)	9.96 (8.75–11.27)	97.19 (87.23–108.11)	4.83 (4.34–5.38)	− 9.87	− 3.69 (− 4.23− − 3.15)
High	48.13 (46.47–49.30)	4.69 (4.54–4.81)	112.24 (102.49–118.74)	5.89 (5.44–6.21)	133.20	0.42 (0.01–0.83)
**Regions**						
East Asia	237.01 (202.34–279.89)	25.52 (21.98–29.94)	193.86 (163.85–228.76)	9.39 (7.98–11.03)	− 18.20	− 4.98 (− 5.73− − 4.22)
South Asia	15.85 (13.38–18.09)	2.82 (2.33–3.27)	38.65 (33.52–44.56)	2.81 (2.43–3.24)	143.79	− 0.06 (− 0.16–0.04)
Southeast Asia	17.57 (15.68–19.28)	6.76 (6.03–7.45)	42.86 (35.33–51.52)	7.33 (6.08–8.79)	143.90	0.28 (0.20–0.36)
Central Asia	1.51 (1.35–1.66)	3.24 (2.89–3.58)	6.19 (5.39–7.08)	8.72 (7.63–9.88)	310.70	2.93 (2.42–3.45)
High-income Asia Pacific	23.59 (22.76–24.37)	11.62 (11.18–12.00)	49.68 (43.78–53.50)	10.78 (9.77–11.53)	110.62	− 0.86 (− 1.46− − 0.25)
Oceania	0.11 (0.09–0.13)	3.85 (3.25–4.47)	0.23 (0.19–0.28)	3.46 (2.93–4.09)	106.99	− 0.22 (− 0.29− − 0.16)
Australasia	0.46 (0.44–0.48)	1.98 (1.90–2.06)	2.01 (1.83–2.17)	4.12 (3.80–4.46)	332.62	2.88 (2.67–3.09)
Eastern Europe	4.22 (4.06–4.41)	1.55 (1.49–1.62)	9.68 (8.51–11.12)	2.87 (2.51–3.29)	129.05	2.52 (2.27–2.77)
Western Europe	19.88 (19.16–20.42)	3.43 (3.31–3.52)	40.30 (37.22–42.88)	4.41 (4.10–4.68)	102.67	0.80 (0.66–0.94)
Central Europe	8.11 (7.83–8.32)	5.60 (5.38–5.75)	7.20 (6.22–8.33)	3.36 (2.90–3.90)	− 11.24	− 1.49 (− 1.87− − 1.11)
High-income North America	7.07 (6.78–7.25)	2.03 (1.95–2.07)	26.48 (23.64–28.91)	4.29 (3.83–4.68)	274.32	2.66 (2.52–2.80)
Andean Latin America	1.07 (0.95–1.21)	5.23 (4.60–5.87)	1.84 (1.51–2.23)	3.34 (2.73–4.03)	71.18	− 1.94 (− 2.37− − 1.51)
Central Latin America	3.07 (2.86–3.23)	3.74 (3.46–3.94)	8.42 (7.36–9.75)	3.65 (3.18–4.22)	173.76	0.04 (− 0.28–0.35)
Caribbean	1.64 (1.52–1.74)	6.29 (5.85–6.68)	1.69 (1.42–2.01)	3.29 (2.76–3.89)	3.58	− 2.16 (− 2.97− − 1.35)
Tropical Latin America	1.88 (1.80–1.95)	2.09 (1.99–2.17)	5.94 (5.54–6.24)	2.50 (2.32–2.62)	215.17	1.06 (0.91–1.21)
Southern Latin America	0.76 (0.68–0.83)	1.65 (1.49–1.81)	2.03 (1.90–2.15)	2.41 (2.26–2.56)	168.33	2.00 (1.78–2.23)
Eastern Sub-Saharan Africa	2.54 (2.08–3.15)	3.15 (2.63–3.99)	5.68 (4.68–6.92)	3.41 (2.85–4.15)	123.76	0.08 (− 0.04–0.21)
Southern Sub-Saharan Africa	1.91 (1.35–3.14)	6.74 (4.71–11.07)	4.04 (3.62–4.54)	7.05 (6.31–7.91)	111.14	− 0.43 (− 1.01–0.16)
Western Sub-Saharan Africa	5.31 (4.55–6.17)	5.81 (4.99–6.76)	9.97 (8.36–11.56)	5.29 (4.48–6.04)	87.85	− 0.44 (− 0.51− − 0.36)
North Africa and Middle East	10.91 (9.58–12.23)	6.39 (5.55–7.19)	26.43 (21.21–32.61)	6.20 (5.06–7.62)	142.20	0.25 (0.10–0.39)
Central Sub-Saharan Africa	0.72 (0.60–0.85)	2.85 (2.43–3.29)	1.39 (1.11–1.75)	2.47 (1.99–3.07)	94.59	− 0.64 (− 0.71− − 0.57)

*EAPC* estimated annual percentage change, *ASDR* age-standardized death rate, *CI* confidence interval, *UI* uncertainty interval, *SDI* socio-demographic index

**Table 2:** The number of death due to liver cancer caused by specific etiologies in 2019 and the percentage changes in number during 1990–2019 in age groups

Age Groups	LC		LCHB		LCHC		LCAL		LCNA	
	Number × 10^3 ^(95% UI)	Change in number (%)	Number × 10^3 ^(95% UI)	Change in number (%)	Number × 10^3 ^(95% UI)	Change in number (%)	Number × 10^3 ^(95% UI)	Change in number (%)	Number × 10^3 ^(95% UI)	Change in number (%)
**5 to 9**	0.63 (0.52–0.75)	− 15.53	0	0	0	0	0	0	0	0
**10 to 14**	0.93 (0.78–1.11)	− 16.37	0.34 (0.26–0.43)	− 37.00	0.01 (0.01–0.02)	− 1.61	0	0	0	0
**15 to 19**	0.70 (0.62–0.79)	− 45.64	0.37 (0.30–0.45)	− 56.92	0.01 (0.01–0.02)	− 19.20	0.01 (0–0.02)	− 4.66	0.14 (0.10–0.19)	2.87
**20 to 24**	1.17 (1.05–1.30)	− 46.46	0.75 (0.65–0.87)	− 53.59	0.03 (0.02–0.05)	− 18.53	0.05 (0.03–0.07)	5.53	0.15 (0.11–0.20)	0.56
**25 to 29**	2.33 (2.10–2.57)	− 32.02	1.70 (1.45–1.96)	− 37.00	0.08 (0.05–0.14)	− 10.16	0.12 (0.07–0.20)	17.13	0.17 (0.12–0.25)	10.57
**30 to 34**	5.38 (4.78–6.05)	− 21.95	4.10 (3.50–4.76)	− 26.08	0.25 (0.17–0.36)	− 2.27	0.34 (0.23–0.50)	20.70	0.27 (0.19–0.37)	16.16
**35 to 39**	8.99 (7.84–10.21)	− 42.52	6.57 (5.44–7.77)	− 47.01	0.59 (0.38–0.91)	− 23.50	0.82 (0.51–1.25)	− 2.49	0.40 (0.28–0.56)	− 11.01
**40 to 44**	15.84 (13.81–18.10)	− 28.78	11.06 (9.08–13.29)	− 34.37	1.40 (0.96–1.92)	− 12.30	1.74 (1.13–2.52)	9.07	0.68 (0.48–0.93)	3.10
**45 to 49**	26.19 (22.58–29.98)	− 2.05	16.92 (13.64–20.87)	− 10.33	3.15 (2.16–4.37)	12.33	3.45 (2.15–5.01)	39.08	1.20 (0.83–1.73)	35.52
**50 to 54**	40.26 (35.32–45.36)	8.02	23.07 (18.59–28.25)	− 4.34	6.70 (4.86–8.81)	23.15	6.29 (4.09–9.02)	52.13	2.06 (1.44–2.94)	49.80
**55 to 59**	50.31 (45.15–56.27)	6.37	24.94 (19.96–30.86)	− 9.85	10.79 (7.83–14.35)	16.75	9.30 (6.39–12.67)	56.85	2.84 (1.92–4.04)	49.42
**60 to 64**	59.69 (54.22–65.19)	18.78	25.60 (20.19–31.80)	− 0.29	15.06 (11.15–19.69)	23.37	12.59 (8.83–16.83)	65.73	3.76 (2.58–5.41)	64.57
**65 to 69**	67.13 (61.81–72.56)	38.91	25.17 (19.45–31.42)	17.20	18.81 (14.96–22.83)	40.11	15.64 (11.64–20.56)	81.99	4.62 (3.26–6.56)	83.97
**70 to 74**	64.25 (59.36–69.40)	60.22	20.23 (14.99–25.75)	29.52	20.94 (16.98–24.99)	61.21	15.29 (11.4–20.14)	115.08	5.09 (3.71–6.93)	106.67
**75 to 79**	55.89 (51.66–59.75)	79.51	14.76 (11.47–18.47)	49.36	21.51 (17.24–25.77)	77.74	11.98 (9.17–14.99)	126.15	5.25 (3.83–7.13)	125.23
** > 80**	83.15 (70.45–90.63)	202.62	16.15 (11.97–20.53)	149.70	42.46 (34.28–49.41)	210.77	13.12 (9.74–16.69)	241.84	8.09 (5.76–10.76)	257.80

*LC* liver cancer, *LCHB* liver cancer due to hepatitis B, *LCHC* liver cancer due to hepatitis C, *LCAL* liver cancer due to alcohol use, *LCNA* liver cancer due to non-alcoholic steatohepatitis

### Regional and national burden of liver cancer

**Fig. 3:**
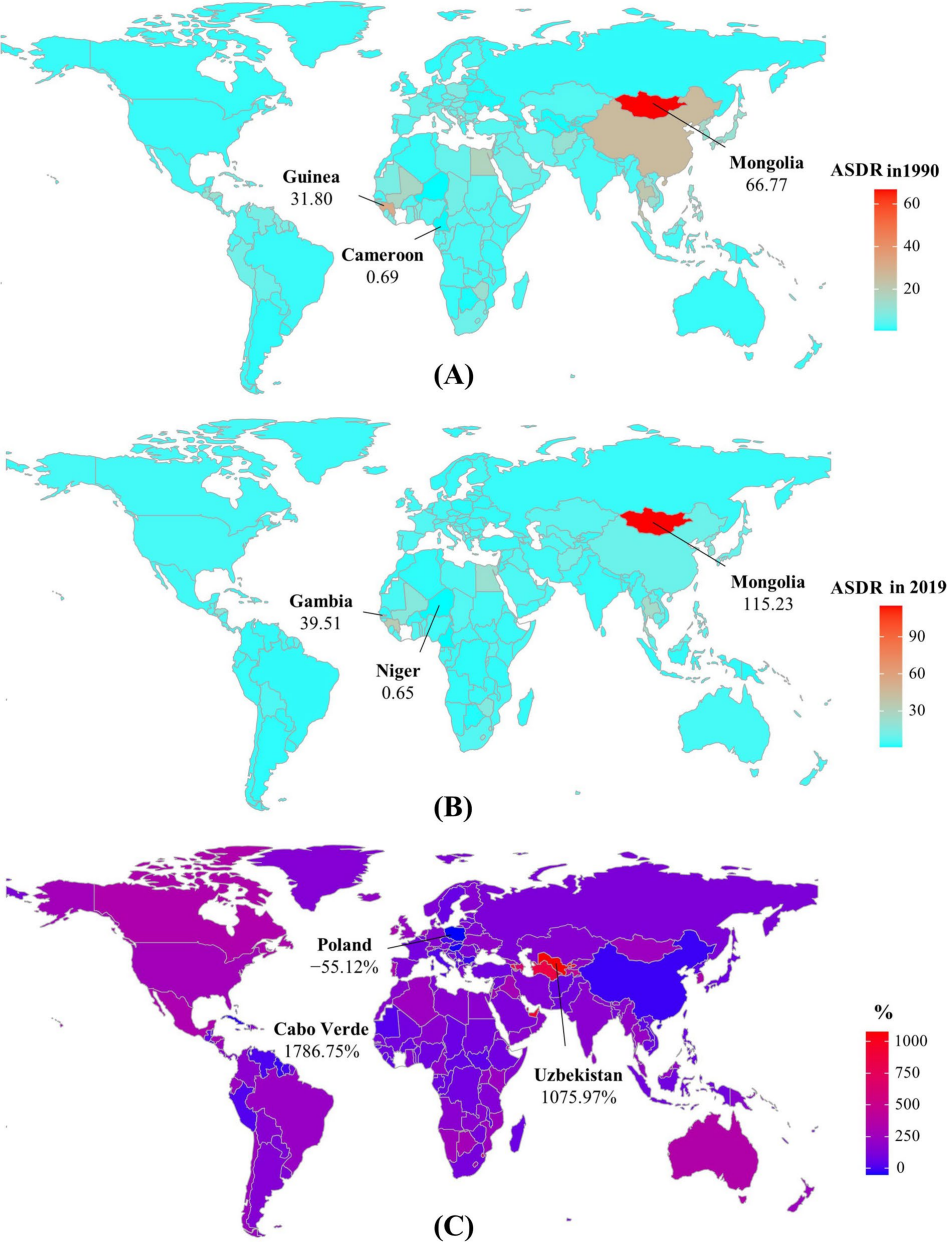
The distribution of ASDR and percentage changes in death number of liver cancer at the national level. **A** and **B** The ASDR of caused by liver cancer in 1990 and 2019, respectively; **C** The percentage changes in death number of liver cancer between 1990 and 2019. Countries/territories with an extreme value were annotated. ASDR, age-standardized death rate

**Fig. 4:**
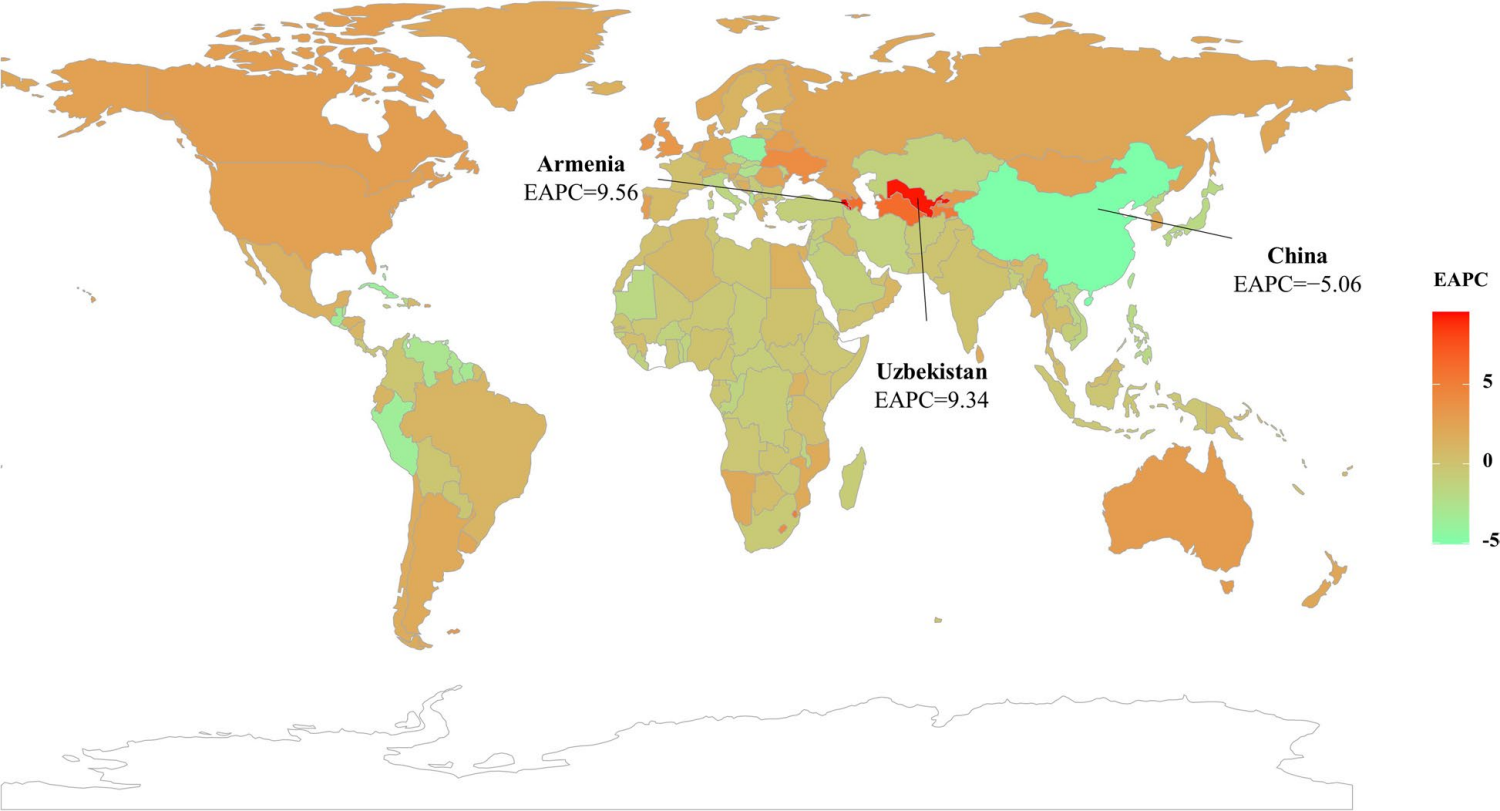
The distribution of EAPCs of death caused by liver cancer at the national level. Countries/territories with an extreme value were annotated. EAPC, estimated annual percentage change

The decreasing trends of LC were observed in both sexes and most socio-demographic index (SDI) areas, particularly the high-middle SDI area (EAPC =  − 3.69, 95% CI: − 4.23 to − 3.15) (Table 1, Fig. 2B). In terms of geographic regions, the ASDR of LC showed increasing trends in eleven regions, particularly Central Asia (EAPC = 2.93, 95% CI: 2.42 to 3.45) (Table 1). However, decreasing trends were demonstrated in ten regions, particularly East Asia (EAPC =  − 4.98, 95% CI: − 5.73 to − 4.22) (Table 1, Fig. 2C). In 1990, the highest ASDR among the 204 nations and territories was in Mongolia and Guinea, while the lowest was in Cameroon (Fig. 3A, Supplementary table 1). Mongolia continues to have the highest ASDR at 115.23 (91.48–142.48), followed by Gambia worldwide in 2019; While Niger has the lowest ASR of death (Fig. 3B). The most pronounced increasing percentage in number occurred in Cabo Verde (1786.75%), whereas the largest decreasing change was seen in Poland (− 55.12%) (Fig. 3C). Decreasing trends were observed in 107 countries/territories of which, particularly in China, with the respective EAPC of − 5.06 (95% CI: − 5.84 to − 4.27). Additionally, increasing trends were seen in ninety-seven countries/territories, and the largest one was Armenia (EAPC = 9.56, 95% CI: 8.02 to 11.12), followed by Uzbekistan (Fig. 4, Supplementary table 1).

### Burden of liver cancer by human development index

**Fig. 5:**
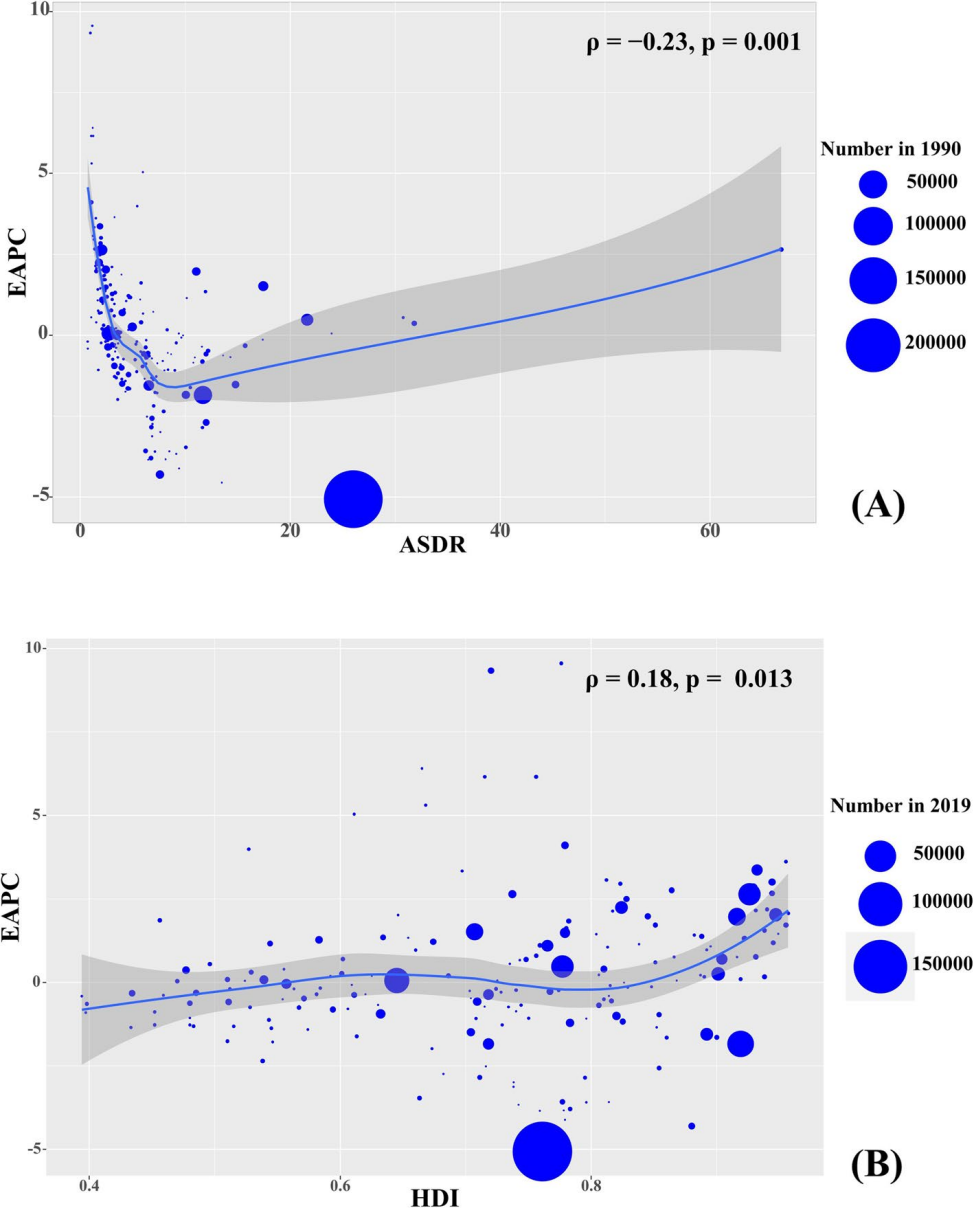
The relationship between EAPCs and ASDR at the national level. The association was calculated with Pearson correlation analysis. The size of circle increase with the corresponding death number in 1990. EAPC, estimated annual percentage change; ASDR, age-standardized death rate

The trends of EAPC of death caused by liver cancer had a negative association with ASDR at a national level in 1990 (*ρ* =  − 0.23, *P* = 0.001, Fig. 5A), but not with the Human Development Index (HDI) in 2019 (ρ = 0.18, *P* = 0.013, Fig. 5B). Similar correlations were also seen in the four etiologies of liver cancer (Supplementary Fig. 1A-D, Supplementary Fig. 2A-D). Overall, the decreasing trends of death due to liver cancer and its etiologies generally occurred in the countries with high HDI, while increasing trends were more common in countries with low HDI.

### Trends of death caused by liver cancer due to four etiologies

#### Liver cancer burden due to hepatitis B (LCHB)

During the period 1990–2019, the death number of LCHB was 191.74 × 10^3^ (95%UI: 161.86 × 10^3^ to 223.73 × 10^3^) worldwide in 2019, with an increase of 0.76% since 1990. The ASDR of LCHB showed a decreasing trend from 1990 to 2019 (EAPC =  − 3.46, 95% CI: − 4.01 to − 2.89) (Fig. 1, Supplementary table 2). In age groups, the highest death number of LCHB was seen in the group aged 60–64, and the increasing percentage changes occurred in populations aged > 65 years (Table 2, Supplementary Fig. 3A). Decreasing trends of LCHB were observed in both sexes, most of SDI areas and geographic regions. The high-middle SDI area and East Asia region presented the EAPCs in − 4.85 (95% CI: − 5.54 to − 4.15) and − 5.11 (95% CI: − 5.88 to − 4.33), respectively (Supplementary table 2, Supplementary Fig. 3B). However, the most pronounced increasing trends were seen in high-income North America and Australasia, with the respective EAPCs were 2.34 (95% CI: 2.15 to 2.52) and 2.29 (95% CI: 2.08 to 2.50) (Supplementary table 2, Supplementary Fig. 3C). At the national level, decreasing trends of LCHB were demonstrated in 126 countries/territories, particularly China (EAPC =  − 5.17, 95% CI: − 5.96 to − 4.37), followed by Saint Kitts and Nevis and Poland. On the contrary, increasing trends were observed in 78 countries/territories, particularly Uzbekistan and Armenia, in which the respective EAPCs were 9.53 (95% CI: 8.31 to 10.77) and 9.21 (95% CI: 7.65 to 10.79) (Supplementary table 3, Supplementary Fig. 4A-C).

#### Liver cancer burden due to hepatitis C (LCHC)

LCHC caused 141.81 × 10^3^ (95%UI: 121.79 × 10^3^ to 161.83 × 10^3^) death in 2019, with an increase of 67.50% since 1990. Decreasing trend in ASDR of LCHC was observed worldwide from 1990 to 2019, in which the EAPC was − 1.35 (95% CI: − 1.59 to − 1.11) (Fig. 1, Supplementary table 2). During the period 1990–2019, the death number of LCHC declined in the age groups under 45 years, while increased in objects aged > 45 years (Table 2, Supplementary Fig. 5A). The trends of LCHC pronouncedly declined in SDI areas except the high SDI area (EAPC = 0.10, 95% CI: − 0.37 to 0.58) (Supplementary table 2, Supplementary Fig. 5B). Among 21 geographic regions, increasing trends in ASDR were found in ten regions, particularly Central Asia (EAPC = 3.28, 95% CI: 2.74 to 3.81). Whereas decreasing trends were seen in eleven regions, and the most pronounced one was in East Asia (EAPC =  − 4.92 (95% CI: − 5.59 to − 4.24), Supplementary Fig. 5C). At the national level, decreasing trends of LCHC were seen in 107 countries/territories, particularly China (EAPC =  − 5.07, 95% CI: − 5.79 to − 4.35), followed by Poland and Bermuda. Whereas increasing trends occurred in 97 countries/territories, and the most pronounced ones were in Armenia and Uzbekistan, in which respective EAPCs were 9.54 (95% CI: 7.99 to 11.12) and 9.03 (95% CI: 8.04–10.03) (Supplementary table 3, and Supplementary Fig. 6A-C).

#### Liver cancer burden due to alcohol consumption (LCAL)

Globally, the death number of LCAL increased 89.60% since 1990, and was 90.74 × 10^3 ^(73.35 × 10^3^ to 109.4 × 10^3^) in 2019. Decreasing trend in ASDR of LCAL was observed worldwide from 1990 to 2019, with the EAPC of − 0.68 (95% CI: − 0.87 to − 0.49) (Fig. 1, Supplementary table 4). During the period 1990–2019, percentages in death number of LCAL increased in most age group, particularly the group over 80 years old (241.84%) (Table 2, Supplementary Fig. 7A). The ASDR of LCAL showed decreasing trends in both sexes and most SDI areas, except the high SDI area (EAPC = 1.03 [95% CI: 0.79 to 1.26]) (Supplementary table 4, and Supplementary Fig. 7B). Among 21 geographic regions, decreasing trend was found in eight regions, particularly East Asia (EAPC =  − 4.40, 95% CI: − 5.19 to − 3.60). Whereas increasing trends were seen in thirteen regions, particularly Eastern Europe and Central Asia regions, in which the respective EAPCs were 2.97 (95% CI: 2.66 to 3.28) and 2.94 (95% CI: 2.41 to 3.46) (Supplementary Fig. 7C). At the national level, the highest increase in death number of LCAL was observed in Cabo Verde (2060.08%), whereas the largest decreasing one was in Hungary (− 51.16%). Decreasing trends of LCAL were demonstrated in 94 countries/territories, particularly in China, and Saint Kitts and Nevis, in which the EAPCs were − 4.46 (95% CI: − 5.28 to − 3.63) and − 4.42 (95% CI: − 5.47 to − 3.36), respectively. However, increasing trends were seen in 110 countries/territories. The most pronounced ones were Armenia and Uzbekistan, with the respective EAPCs of 10.45 (95% CI: 8.85–12.08) and 10.06 (95% CI: 8.97 to 11.17) (Supplementary table 5, Supplementary Fig. 8A-C).

#### Liver cancer burden due to non-alcoholic steatohepatitis (LCNA)

The death number of LCNA was 34.73 × 10^3^ (95%UI: 28.39 × 10^3^ to 43.18 × 10^3^) globally in 2019, with an increase of 95.10% since 1990. The ASDR of LCNA showed a decreasing trend from 1990 to 2019 (EAPC =  − 0.74, 95% CI: − 1.02 to − 0.46) (Fig. 1, Supplementary table 4). The death number of LCNA increased in most age groups, particularly in whom above 80 years old (257.80%) (Table 2, Supplementary Fig. 9A). Decreasing trends of LCNA were observed in both sexes and most SDI areas, but increasing trend was observed in the high SDI area (EAPC = 1.45, 95% CI: 1.09 to 1.81) (Supplementary table 4, Supplementary Fig. 9B). Among 21 geographic regions, increasing trends were seen in fifteen regions, particularly Central Asia (EAPC = 4.14, 95% CI: 3.64 to 4.65). However, decreasing trends were observed in six regions, particularly East Asia (EAPC =  − 4.10, 95% CI: − 4.86 to − 3.32) (Supplementary Fig. 9C). At the national level, increasing trends were observed in 138 countries/territories, particularly Armenia and Uzbekistan, with the respective EAPCs were 10.87 (95% CI: 9.25–12.51) and 10.39 (95% CI: 9.44–11.34). Moreover, decreasing trends were seen in 66 countries//territories. The countries in significant decline were Poland and China, with the respective EAPCs of − 4.38 (95% CI: − 5.73 to − 3.01) and − 4.20 (95% CI: − 5.01 to − 3.39) (Supplementary table 5, Supplementary Fig. 10A-C).

### Trends of death caused by liver cancer due to underlying etiologies attributable risks

**Fig. 6:**
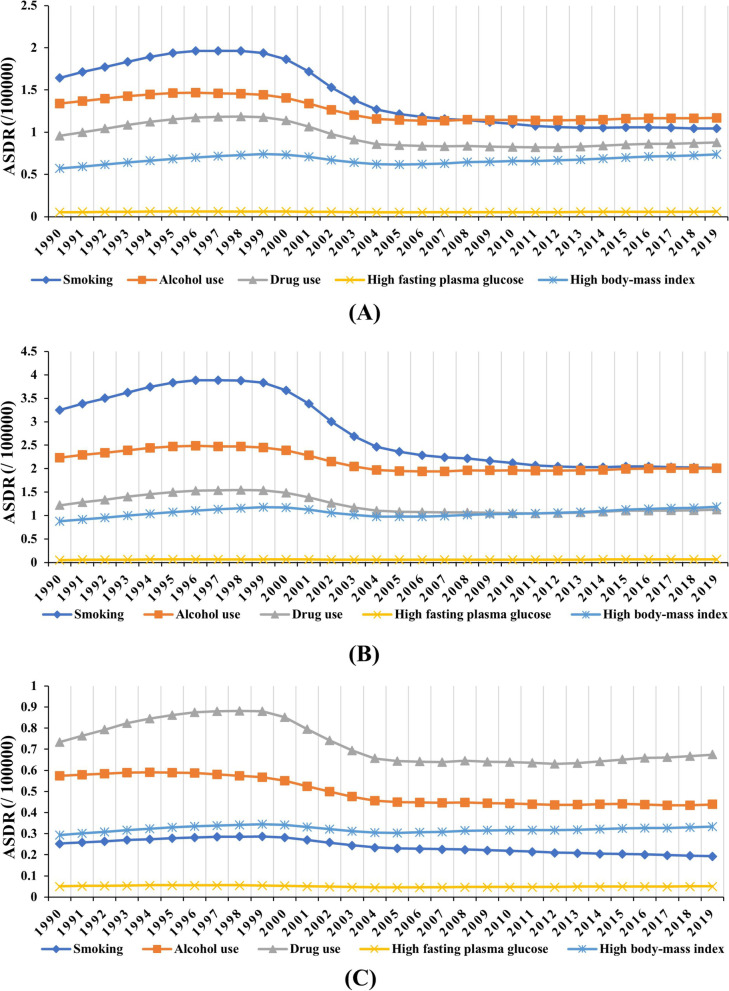
The distribution of ASDR of liver cancer due to attributable risks. ASDR of liver cancer due to risk factors including smoking, alcohol consumption, drug use, high fasting plasma glucose, high body-mass index from 1990 to 2019. ASDR of death caused by liver cancer due to risk factors worldwide in both sexes (**A**), male (**B**), and female (**C**), respectively. ASDR of death caused by liver cancer due to risk factors worldwide in females. ASDR, age-standardized death rate

**Fig. 7:**
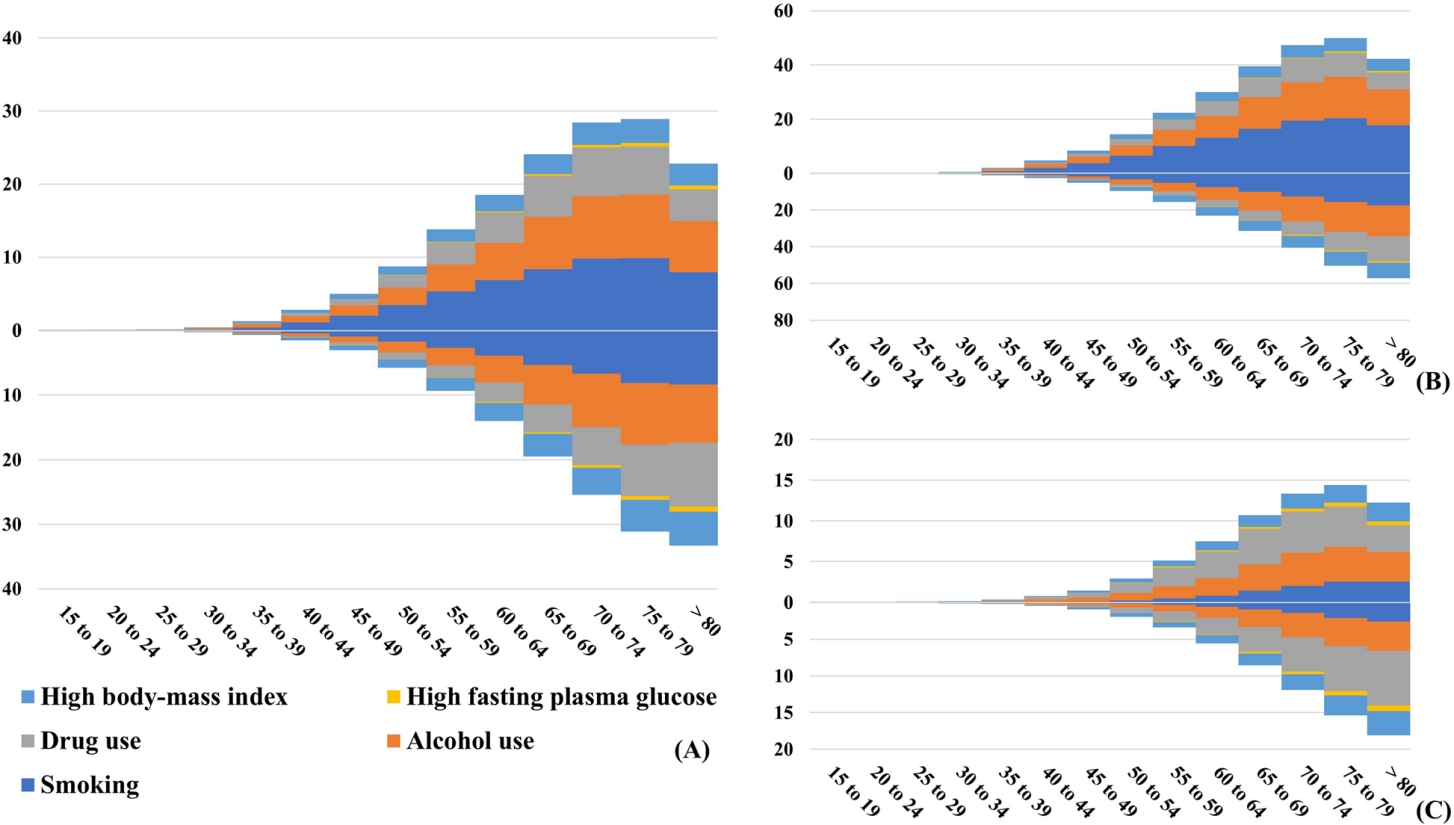
The overall rate of death caused by liver cancer by age, sex, and attributable risks. The upper column in each group is the data in 1990, and the lower column is in 2019. All age death rate caused by liver cancer worldwide in both sexes, male, and female were (**A**), (**B**), and (**C**), respectively

During the period 1990–2019, decreasing trends were observed in smoking-, alcohol use-, and drug use-related death caused by LC worldwide particularly smoking-related (EAPC =  − 2.62, 95% CI: − 3.06 to − 2.16, Table 3, Fig. 6A). However, increasing trend was seen in the high body-mass index (BMI)-related death caused by LC (EAPC = 0.31, 95% CI: 0.05 to 0.58) (Table 3, Fig. 6A). After stratified analysis, we found that the overall rate of death caused by liver cancer by attributable risks in different age groups in 2019 demonstrated an increase when compared to the results in 1990 (Fig. 7A). In the groups over 80s, there was a clear trend towards an increasing number of deaths due to the four attributable risks, particularly alcohol use and drug use (Fig. 7A). Compared with female, male had more pronounced decreasing trends in risks-related death of LC, particularly in smoking-related death (EAPC =  − 2.73, 95% CI: − 3.19 to − 2.26) (Table 4, Fig. 6B and 6C). Smoking is the most important contributory risk among male, while drug use is the most significant contributory risk among women (Fig. 7B and C).

**Table 3:** The number and age-standardized rate of death caused by liver cancer and underlying etiologies in risks globally, in both sexes, in 1990 and 2019, and percentage changes in number and the EAPCs from 1990 to 2019

	**1990**		**2019**		**1990 − 2019**	
**Characteristics**	Number × 10^3^ (95% UI)	ASDR per 100k (95% UI)	Number × 10^3^ (95% UI)	ASDR per 100k (95% UI)	Change in number (%)	EAPC (95% CI)
**LC**						
Smoking	66.46 (36.89–95.85)	1.64 (0.91–2.36)	85.88 (50.01–122.99)	1.04 (0.61–1.49)	29.23	− 2.62 (− 3.06− − 2.16)
Alcohol use	54.07 (42.16–67.93)	1.34 (1.05–1.67)	96.05 (77.51–116.17)	1.17 (0.94–1.41)	77.66	− 0.98 (− 1.22− − 0.74)
Drug use	38.52 (28.99–49.48)	0.96 (0.72–1.23)	71.45 (57.09–89.24)	0.88 (0.71–1.10)	85.48	− 1.19 (− 1.57− − 0.81)
High fasting plasma glucose	1.99 (0.45–4.43)	0.05 (0.01–0.11)	4.73 (1.15–10.41)	0.06 (0.01–0.13)	137.15	− 0.17 (− 0.43–0.10)
High body-mass index	23.18 (6.96–52.46)	0.57 (0.17–1.29)	60.80 (24.24–114.62)	0.74 (0.29–1.39)	162.34	0.31 (0.05–0.58)
**LCHB**						
Smoking	36.89 (19.39–55.63)	0.89 (0.47–1.33)	38.07 (20.93–56.37)	0.46 (0.25–0.67)	3.20	− 3.72 (− 4.35− − 3.09)
Alcohol use	5.58 (0.15–14.60)	0.12 (0–0.33)	4.58 (0.20–11.74)	0.05 (0–0.14)	− 17.92	− 4.52 (− 5.34− − 3.69)
Drug use	1.76 (1.10–2.68)	0.04 (0.03–0.06)	3.12 (2.10–4.60)	0.04 (0.03–0.06)	77.42	− 1.44 (− 1.94− − 0.95)
High body-mass index	10.80 (2.69–26.45)	0.25 (0.06–0.62)	24.07 (8.74–48.33)	0.29 (0.10–0.58)	122.95	− 0.57 (− 1.04− − 0.10)
**LCHC**						
Smoking	14.09 (7.72–19.94)	0.37 (0.20–0.52)	21.95 (12.52–31.73)	0.27 (0.16–0.39)	55.74	− 1.74 (− 2.07− − 1.41)
Alcohol use	0.63 (0.01–2.25)	0.02 (0–0.06)	0.73 (0.01–2.65)	0.01 (0–0.03)	16.52	− 3.34 (− 3.91− − 2.78)
Drug use	36.76 (27.51–47.29)	0.91 (0.69–1.18)	68.33 (54.6–85.4)	0.84 (0.67–1.05)	85.87	− 1.18 (− 1.55− − 0.80)
High body-mass index	6.59 (2.13–14.13)	0.17 (0.06–0.37)	19.07 (7.58–35.43)	0.24 (0.09–0.44)	189.50	0.77 (0.60–0.95)
**LCAL**						
Smoking	10.22 (5.65–15.05)	0.26 (0.14–0.38)	17.66 (9.74–26.16)	0.21 (0.12–0.32)	72.78	− 1.12 (− 1.33− − 0.91)
Alcohol use	47.86 (38.59–58.61)	1.20 (0.97–1.46)	90.74 (73.35–109.40)	1.10 (0.89–1.33)	89.60	− 0.68 (− 0.87− − 0.49)
High body-mass index	4.55 (1.56–9.70)	0.11 (0.04–0.24)	14.64 (5.56–28.32)	0.18 (0.07–0.34)	221.53	1.37 (1.28–1.46)
**LCNA**						
Smoking	2.58 (1.42–3.87)	0.07 (0.04–0.10)	4.96 (2.79–7.54)	0.06 (0.03–0.09)	92.28	− 1.04 (− 1.38− − 0.71)
High fasting plasma glucose	1.02 (0.24–2.29)	0.03 (0.01–0.06)	3.04 (0.74–6.87)	0.04 (0.01–0.09)	196.45	0.69 (0.48–0.89)

*LCHB* liver cancer due to hepatitis B, *LCHC* liver cancer due to hepatitis C, *LCAL* liver cancer due to alcohol use, *LCNA* liver cancer due to non- alcoholic steatohepatitis, *EAPC* estimated annual percentage change, *ASDR* age-standardized death rate, *CI* confidence interval

**Table 4:** The age-standardized rate of liver cancer death attributed to risks globally in sexes and 2019, and percentage changes in number and the EAPCs from 1990 to 2019

	**Male**			**Female**		
**Characteristics**	ASDR per 100k No. (95% UI)	Change in number (%)	EAPC No. (95% CI)	ASDR per 100k No. (95% UI)	Change in number (%)	EAPC No. (95% CI)
**LC**						
Smoking	2.02 (1.18–2.87)	26.50	− 2.73 (− 3.19− − 2.26)	0.19 (0.10–0.30)	60.75	− 1.36 (− 1.59− − 1.13)
Alcohol use	2.01 (1.64–2.42)	83.67	− 0.88 (− 1.13− − 0.64)	0.44 (0.34–0.55)	57.10	− 1.34 (− 1.55− − 1.13)
Drug use	1.13 (0.92–1.38)	83.75	− 1.23 (− 1.63− − 0.82)	0.67 (0.50–0.88)	87.99	− 1.11 (− 1.45− − 0.76)
High fasting plasma glucose	0.07 (0.01–0.16)	165.46	0.07 (− 0.25–0.39)	0.05 (0.01–0.12)	112.21	− 0.41 (− 0.63− − 0.19)
High body-mass index	1.19 (0.40–2.41)	172.72	0.41 (0.12–0.70)	0.33 (0.06–0.74)	134.06	0.06 (− 0.10–0.23)
**LCHB**						
Smoking	0.92 (0.52–1.35)	2.52	− 3.76 (− 4.40− − 3.13)	0.03 (0.02–0.05)	24.94	− 2.41 (− 2.74− − 2.09)
Alcohol use	0.11 (0–0.28)	− 18.06	− 4.49 (− 5.31− − 3.66)	0	18.43	− 2.23 (− 2.77− − 1.69)
Drug use	0.07 (0.05–0.10)	73.04	− 1.59 (− 2.13− − 1.05)	0.01 (0.01–0.02)	110.32	− 0.14 (− 0.32–0.04)
High body-mass index	0.51 (0.17–1.06)	129.42	− 0.51 (− 1.00− − 0.01)	0.08 (0.01–0.18)	91.44	− 0.81 (− 1.11− − 0.51)
**LCHC**						
Smoking	0.49 (0.28–0.71)	52.96	− 1.87 (− 2.21− − 1.52)	0.10 (0.05–0.15)	68.44	− 1.23 (− 1.48− − 0.99)
Alcohol use	0.02 (0–0.06)	6.80	− 3.55 (− 4.14− − 2.95)	0 (0–0.01)	99.20	− 1.35 (− 2.00− − 0.71)
Drug use	1.06 (0.86–1.30)	84.53	− 1.21 (− 1.60− − 0.81)	0.66 (0.49–0.87)	87.69	− 1.12 (− 1.46− − 0.77)
High body-mass index	0.31 (0.10–0.62)	213.22	1.02 (0.80–1.23)	0.17 (0.03–0.37)	159.09	0.45 (0.31–0.58)
**LCAL**						
Smoking	0.43 (0.24–0.63)	73.54	− 1.18 (− 1.4− − 0.97)	0.03 (0.01–0.05)	63.43	− 1.03 (− 1.13− − 0.93)
Alcohol use	1.88 (1.53–2.27)	100.72	− 0.52 (− 0.7− − 0.34)	0.44 (0.33–0.55)	56.95	− 1.34 (− 1.55− − 1.13)
High body-mass index	0.32 (0.11–0.63)	239.37	1.49 (1.40–1.58)	0.05 (0.01–0.12)	151.92	0.54 (0.45–0.62)
**LCNA**						
Smoking	0.11 (0.06–0.16)	89.65	− 1.24 (− 1.62− − 0.87)	0.02 (0.01–0.04)	103.35	− 0.38 (− 0.57− − 0.20)
High fasting plasma glucose	0.04 (0.01–0.10)	234.67	0.91 (0.66–1.17)	0.03 (0.01–0.08)	163.91	0.46 (0.30–0.62)

*LCHB* liver cancer due to hepatitis B, *LCHC* liver cancer due to hepatitis C, *LCAL* liver cancer due to alcohol use, *LCNA* liver cancer due to non-alcoholic steatohepatitis, *EAPC* estimated annual percentage change, *ASDR* age-standardized death rate, *CI* confidence interval

In the SDI level, smoking- and alcohol consumption-related death of LC showed decreasing trends in most SDI areas, and the largest one was smoking-related death in high-middle SDI area (EAPC =  − 3.91, 95% CI: − 4.52 to − 3.29, Table 5). Whereas high fasting plasma glucose- and high BMI-related death presented increasing trends in most of SDI areas, particularly the high fasting plasma glucose-related deaths in high SDI area (EAPC = 2.82, 95% CI: 2.58 to 3.06). During the period 1990–2019, smoking-, alcohol use- and drug use-related death caused by four specific etiologies showed decreasing trends in both sexes and most SDI areas, particularly alcohol use caused by LCHB in the high-middle SDI area (EAPC =  − 5.59, 95%I: − 6.48 to − 4.69) (Table 5, Supplementary Fig. 11A, Supplementary Fig. 12). In terms of drug use, the most pronounced decreasing trend was observed in the etiology of LCHC in middle SDI area (EAPC =  − 2.85, 95% CI: − 3.51 to − 2.18). Whereas the increasing trends of drug use occurred in high- SDI areas, particularly in the four specific etiologies of LCHB, with EAPC of 2.17 (95% CI: 1.94 to 2.40) (Table 5, Supplementary Fig. 11B, Supplementary Fig. 13). However, high BMI- and high fasting plasma glucose-related death caused by four specific etiologies showed increasing trends in both sexes and most SDI areas. The most pronounced increasing trend was observed in the high fasting plasma glucose-related death caused by LCNA in high SDI area, with the EAPC of 3.11 (95% CI: 2.84 to 3.38) (Table 5, Supplementary Fig. 11C, Supplementary Fig. 14). In terms of high BMI, the largest increasing trend was observed in the etiology of LCAL in low-middle SDI area (EAPC = 2.57, 95% CI: 2.41 to 2.73) (Table 5, Supplementary Fig. 11D, Supplementary Fig. 15).

**Table 5:** The EAPCs of death due to liver cancers underlying etiologies in attributable risks in SDI quintiles from 1990 to 2019

	**Low SDI**	**Low-middle SDI**	**Middle SDI**	**High-middle SDI**	**High SDI**
**Characteristics**	EAPC (95% CI)	EAPC (95% CI)	EAPC (95% CI)	EAPC (95% CI)	EAPC (95% CI)
**LC**					
Smoking	− 0.65 (− 0.76− − 0.55)	− 2.21 (− 2.61− − 1.81)	− 3.35 (− 3.94− − 2.75)	− 3.91 (− 4.52− − 3.29)	− 0.45 (− 0.89− − 0.01)
Alcohol use	− 0.17 (− 0.23− − 0.11)	− 0.48 (− 0.67− − 0.28)	− 1.76 (− 2.21− − 1.31)	− 2.26 (− 2.61− − 1.92)	0.90 (0.65–1.15)
Drug use	1.21 (1.09–1.33)	− 0.33 (− 0.59− − 0.08)	− 2.82 (− 3.48− − 2.15)	− 2.54 (− 3.03− − 2.05)	1.78 (1.41–2.15)
High fasting plasma glucose	1.11 (1.01–1.20)	0.34 (0.10–0.58)	− 1.30 (− 1.75− − 0.84)	− 1.91 (− 2.26− − 1.56)	2.82 (2.58–3.06)
High body-mass index	1.62 (1.57–1.67)	1.31 (1.07–1.56)	0.05 (− 0.37–0.47)	− 1.02 (− 1.38− − 0.66)	1.70 (1.35–2.04)
**LCHB**					
Smoking	− 0.88 (− 1.02− − 0.74)	− 3.11 (− 3.67− − 2.56)	− 4.09 (− 4.78− − 3.41)	− 5.05 (− 5.82− − 4.27)	− 0.47 (− 1.08–0.13)
Alcohol use	− 0.73 (− 0.94− − 0.52)	− 4.31 (− 5.23− − 3.38)	− 4.48 (− 5.39− − 3.56)	− 5.59 (− 6.48− − 4.69)	− 1.68 (− 2.33− − 1.04)
Drug use	1.03 (0.91–1.14)	− 1.61 (− 2.16− − 1.05)	− 2.11 (− 2.73− − 1.49)	− 2.66 (− 3.33− − 1.98)	2.17 (1.94–2.40)
High body-mass index	1.37 (1.33–1.42)	0.47 (0.08–0.86)	− 0.75 (− 1.34− − 0.16)	− 1.89 (− 2.47− − 1.31)	1.42 (0.96–1.89)
**LCHC**					
Smoking	− 0.66 (− 0.75− − 0.58)	− 1.25 (− 1.44− − 1.07)	− 2.37 (− 2.79− − 1.94)	− 2.74 (− 3.18− − 2.31)	− 1.04 (− 1.55− − 0.54)
Alcohol use	− 0.87 (− 0.96− − 0.78)	− 3.46 (− 4.35− − 2.56)	− 4.48 (− 5.54− − 3.41)	− 5.26 (− 6.12− − 4.39)	− 2.71 (− 3.40− − 2.01)
Drug use	1.22 (1.10–1.34)	− 0.26 (− 0.50− − 0.03)	− 2.85 (− 3.51− − 2.18)	− 2.53 (− 3.01− − 2.05)	1.77 (1.39–2.14)
High body-mass index	1.56 (1.49–1.63)	1.82 (1.69–1.95)	0.82 (0.62–1.01)	− 0.43 (− 0.63− − 0.22)	1.31 (0.91–1.71)
**LCAL**					
Smoking	− 0.49 (− 0.58− − 0.40)	− 1.02 (− 1.24− − 0.79)	− 1.72 (− 2.10− − 1.34)	− 2.07 (− 2.37− − 1.78)	0.28 (0.02–0.54)
Alcohol use	− 0.17 (− 0.23− − 0.10)	− 0.33 (− 0.49− − 0.16)	− 1.41 (− 1.80− − 1.03)	− 1.90 (− 2.18− − 1.62)	1.03 (0.79–1.26)
High body-mass index	2.13 (2.09–2.17)	2.57 (2.41–2.73)	1.68 (1.43–1.92)	0.06 (− 0.07–0.19)	2.36 (2.15–2.57)
**LCNA**					
Smoking	− 0.12 (− 0.21− − 0.03)	− 1.04 (− 1.30− − 0.77)	− 1.70 (− 2.18− − 1.21)	− 2.41 (− 2.93− − 1.88)	0.74 (0.39–1.09)
High fasting plasma glucose	1.21 (1.11–1.31)	0.98 (0.80–1.16)	− 0.28 (− 0.67–0.11)	− 0.96 (− 1.24− − 0.68)	3.11 (2.84–3.38)

*LCHB* liver cancer due to hepatitis B, *LCHC* liver cancer due to hepatitis C, *LCAL* liver cancer due to alcohol use, *LCNA* liver cancer due to non-alcoholic steatohepatitis, *EAPC* estimated annual percentage change, *ASDR* age-standardized death rate, *CI* confidence interval

## Discussion

The GBD study from 1990 to 2017 by Lin L et al. [21] showed that the global liver cancer incidence and mortality had been increasing. However, our study revealed that total liver cancer and etiology-specific liver cancer cases all showed a decreasing trend in mortality from 1990 to 2019 globally. For etiology-specific liver cancer cases, the magnitude and rate of decline were more pronounced for liver cancer attributable to HBV and HCV than for liver cancer attributable to other etiologies. Chronic HBV infection has been widely acknowledged as the leading cause of liver cancer worldwide [22]. Considerable progress had achieved in the etiology prevention and therapeutic measures of LC over the past decades. Under the recommendation of World Health Organization, Hepatitis B vaccine for infants were available in 186 countries by 2016, and coverage with the full recommended dose was estimated more than 80% worldwide [23]. Effective prevention of HBV has dramatically declined the incidence of LC in high-risk countries/territories [24]. Meanwhile, liver ultrasonography was the most common LC surveillance test, which was widely available to the high-risk population in many countries [25]. Among the HBsAg carriers, semiannual alpha-fetoprotein (AFP) was sensitive in LC detection, and significantly prolonged survival rates [26]. Chronic HCV infects over 170 million people worldwide. Chronic infection occurs in 50–80% of cases and eventually leads to cirrhosis and hepatocellular carcinoma [27]. Although challenges remain in the development and application of prophylactic vaccines for HCV, advances in HCV treatment with specific drugs have reduced the morbidity and mortality of LC [7, 28, 29]. In general, the decreasing trends of death caused by LCHB and LCHC might be driven by reductions in aflatoxin exposure, increasing hepatitis B vaccination rates and the cumulative effect of hepatitis C viral suppression from new-generation anti-viral agents [30]. And the extensive HBV vaccine coverage now in place augurs even greater risk reductions in the future.

Additionally, our study had estimated the liver cancer burden of the other potential factors (smoking, alcohol use, drug use, high fasting plasma glucose and BMI), regional and economic status. Apparently, the most rapid decline in LC, LCHB and LCHC cases occurred between 2000 and 2005. Whereas, the overall trends of death caused by LC and its underlying etiologies declined slowly, probably due to population growth and aging [5, 31], and the alarming prevalence of unhealthy lifestyle, and metabolic disorders [32, 33]. In addition, there were recent upward trends in liver cancer due to underlying etiologies attributable to high body-mass index (BMI), especially in LCHB cases. Metabolic risk factors for liver cancer will continue to increase in prevalence and may become the dominant risk factor in the next 5 years in western populations. Studies reported that nonalcoholic fatty liver disease promoted the rapidly increase in the LC death [34, 35], and unsatisfied survival for LC patients [7, 36].

High fasting plasma glucose-related caused by LCNA had increasing trends in high SDI and low SDI area, which probably were explained by the high prevalence of obesity, and metabolic syndromes in these areas [37]. Injecting drug use were likely drivers for the spread of the HCV epidemics in North America and Australia [38, 39]. In addition, the injecting drug use-related HCV burden was highest in these high-income countries [40], which explained why the pronounced increasing trends of drug use-related death caused by LCHC occurred in high SDI area. In conclusion, the low HDI countries generally had a higher burden and worse outcomes than the high HDI countries, which also explained why EAPC had a negative relationship with HDI.

Liver cancer was among the top five causes of cancer death in 90 countries. Most of these countries were in Eastern and South-Eastern Asia. Decreasing EAPC for mortality were found in Eastern Asia but no significant change in South-Eastern Asia. This may be related to the long-term vision and cost-effective interventions in high-risk countries (e.g. China) [41]. The most pronounced decreasing trends of death caused by LC were observed in China, which was mainly due to the effective medical-care system [42], particularly the universal coverage of HBV vaccination over the past decades [43, 44]. Meanwhile, a web-based surveillance system well protected children and adolescents from HBV infection across 31 provinces in China over 11 years [45]. Similarly, decreasing trends of LCHB and LCHC were all demonstrated in Poland, whose HBV and HCV infections were well managed using Epidemiological Interview Registration System (SRWE) from 1997 to 2018 [46, 47]. The newborns covered by obligatory hepatitis B vaccinations after 1994, and the third HBV vaccine dose covered 91% of children aged two years [47]. Meanwhile, primary prevention activities emphasized the safer medical procedures and reduction for people who inject drugs [48]. However, the ASDR of LC and underlying etiologies showed the largest increasing trends in Armenia and Uzbekistan. High mortality due to liver cancer was associated with the chloroprene exposure in 1990s, alcohol frequent use, as well as high prevalence of inject drugs and HCV and HIV in the youths in Armenia [49–51]. In Uzbekistan, the seroprevalence of HBV and HCV infections was high, and the transmission of HCV was common in medical treatment and drug abusers [52].

The most pronounced increasing trends were seen in high-income North America with the EAPC was 2.34 (95% CI: 2.15 to 2.52). The incidence and mortality of liver cancer were increasing in America countries as a result of an ageing cohort infected with chronic hepatitis C, and were expected to continue to rise as a consequence of the epidemic by metabolic factors, including metabolic syndrome, obesity and non-alcoholic fatty liver disease [53, 54]. Additionally, we did not observe a decreasing trend in EAPCs for liver cancer mortality in American countries. Information about the prevalence and incidence of, and risk factors for, liver cancer in Latin America is scarce [55]. Mendez-Sanchez N. had shown that the cause-specific mortality rate was 4.1 per 100,000 in 2000 and increased to 4.7 per 100,000 in 2006 in Mexico [56]. The main etiologies of liver cancer were HBV and HCV infection, followed by alcohol abuse alone, cryptogenic cirrhosis and schistosomiasis in Argentina or Brazil patients [57, 58]. Further studies are required to identify accurately the incidence, prevalence, mortality rate, and risk factors in Latin America. Likewise, no appreciable reductions in liver cancer mortality from EAPC between 1990 and 2019 were discovered in African countries. It has been estimated that populations in sub-Saharan Africa have the highest burdens of liver cancer attributable to aflatoxin exposure, particularly as there is a synergistic effect between aflatoxin and HBV infection [41]. And only 1% of the population in Africa were covered by the population-based cancer registries [41].

There were still several limitations in this study. Firstly, the GBD death estimates depend upon the quality and quantity of data. Potential bias due to misclassification or miscoding, such as there was only one broad category of ICDs included as mentioned in data sources, we were unable to perform a subgroup analysis of the disease classification of hepatocellular carcinoma and intrahepatic cholangiocarcinoma for mortality trends, would probably affect the accuracy and reliability of the findings. Secondly, the diagnostic standards of LC and underlying etiologies had refined over time, which complicated the trends estimation of LC. Last but not least, in terms of death caused by LC and four etiologies, only five attributable risks were available in the GBD estimates, but there certainly existed other potential risk factors (such as aflatoxin exposure and HBV vaccine), so risk-related trends cannot be fully assessed. Analysis on birth-cohort effects and others were not involved. Better primary data from a national wide-coverage observational study or cancer registry on liver cancer burden are needed in the future.

## Conclusions

The decreasing trends in death caused by liver cancer and underlying etiologies were observed worldwide from 1990 to 2019. However, increasing trends occurred in low-resource regions and countries (such as Armenia and Uzbekistan). The trends of drug use- and high BMI-related death caused by liver cancer and underlying etiologies were alarming. The findings highlighted that actions should be intensified to reduce the liver cancer death by effective control of etiologies and risk management.

## Supplementary Information

**Additional file 1:** **Supplementary figure 1.** The relationship between EAPCs and ASDR in 1990 at the national level. EAPCs of death due to LCHB (A), LCHC (B), LCAL (B), and LCNA (D) had negative associations with the corresponding ASDR in 1990. The association was calculated with Pearson correlation analysis. The size of circle increases with the corresponding death number in 1990. LCHB, liver cancer due to hepatitis B; LCHC, liver cancer due to hepatitis C; LCAL, liver cancer due to alcohol consumption; LCNA, liver cancer due to non-alcoholic steatohepatitis; EAPCs, estimated annual percentage changes; ASDR, age-standardized death rate. **Supplementary figure 2.** The relationship between EAPCs and HDI in 2019 at the national level. EAPCs of death due to LCHB (A), LCHC (B), LCAL (B), and LCNA (D) had positive associations with HDI in 2019. The association was calculated with Pearson correlation analysis. The size of circle increases with the corresponding death numbers in 2019. LCHB, liver cancer due to hepatitis B; LCHC, liver cancer due to hepatitis C; LCAL, liver cancer due to alcohol consumption; LCNA, liver cancer due to non-alcoholic steatohepatitis; EAPCs, estimated annual percentage changes; HDI, human development index. **Supplementary figure 3.** The distribution of death number of LCHB in age groups, SDI areas, and geographic regions from 1990 to 2019. (A) the death number of LCHB in age groups; (B) the ASDR of LCHB in SDI areas; (C) the ASDR of LCHB in geographical regions. LCHB, liver cancer due to hepatitis B; ASDR, age-standardized death rate; SDI, sociodemographic index. **Supplementary figure 4.** The distribution of percentage changes in number and EAPCs of death caused by LCHB at the national level from 1990 to 2019. (A) The ASDR of LCHB in 2019; (B) The percentage changes in death number of LCHB; (C) EAPCs of death due to LCHB. Countries/territories with an extreme value were annotated. LCHB, liver cancer due to hepatitis B; ASDR, age-standardized death rate; EAPC, estimated annual percentage change. **Supplementary figure 5.** The distribution of death number of LCHC in age groups, SDI areas, and geographic regions from 1990 to 2019. (A) the death number of LCHC in age groups; (B) the ASDR of LCHC in SDI areas; (C) the ASDR of LCHC in geographical regions. LCHC, liver cancer due to hepatitis C; ASDR, age-standardized death rate; SDI, sociodemographic index. **Supplementary figure 6.** The distribution of percentage changes in number and EAPCs of death caused by LCHC at the national level from 1990 to 2019. (A) The ASDR of LCHC in 2019; (B) The percentage changes in death number of LCHC; (C) EAPCs of death due to LCHC. Countries/territories with an extreme value were annotated. LCHC, liver cancer due to hepatitis C; ASDR, age-standardized death rate; EAPC, estimated annual percentage change. **Supplementary figure 7.** The distribution of death number of LCAL in age groups, SDI areas, and geographic regions from 1990 to 2019. (A) the death number of LCAL in age groups; (B) the ASDR of LCAL in SDI areas; (C) the ASDR of LCAL in geographical regions. LCAL, liver cancer due to alcohol use; ASDR, age-standardized death rate; SDI, sociodemographic index. **Supplementary figure 8.** The distribution of percentage changes in number and EAPCs of death caused by LCAL at the national level from 1990 to 2019. (A) The ASDR of LCAL; (B) The percentage changes in number of death due to LCAL; (C) EAPCs of death due to LCAL. Countries/territories with an extreme value were annotated. LCAL, liver cancer due to alcohol use; EAPC, estimated annual percentage change. **Supplementary figure 9.** The distribution of death number of LCNA in age groups, SDI areas, and geographic regions from 1990 to 2019. (A) the death number of LCNA in age groups; (B) the death number of LCNA in SDI areas; (C) the death number of LCNA in geographical regions. LCNA, liver cancer due to non-alcoholic steatohepatitis; ASDR, age-standardized death rate; SDI, sociodemographic index. **Supplementary figure 10.** The distribution of percentage changes in number and EAPCs of death caused by LCNA at the national level from 1990 to 2019. (A) The ASDR of LCNA; (B) The percentage changes in number of death due to LCNA; (C) EAPCs of death due to LCNA. Countries/territories with an extreme value were annotated. LCNA, liver cancer due to non-alcoholic steatohepatitis; EAPC, estimated annual percentage change. **Supplementary figure 11.** The distribution of ASDR of LCHB, LCHC, LCAL, and LCNA due to attributable risks from 1990 to 2019. The ASDR of LCHB, LCHC, LCAL, and LCNA due to attributable risks were (A), (B), (C), and (D), respectively. LCHB, liver cancer due to hepatitis B; LCHC, liver cancer due to hepatitis C; LCAL, liver cancer due to alcohol use; LCNA, liver cancer due to non-alcoholic steatohepatitis; ASDR, age-standardized death rate. **Supplementary figure 12.** The overall death rate of LCHB by sex, age groups, and attributable risks. Death rate due to LCHB in both sexes, male, and female were (A), (B), and (C), respectively. The upper column in each group is data in 1990 and the lower column in 2019. LCHB, liver cancer due to hepatitis B. **Supplementary figure 13.** The death overall rate of caused by LCHC by sex, age groups, and attributable risks. Death rate due to LCHC in both sexes, male, and female were (A), (B), and (C), respectively. The upper column in each group is data in 1990 and the lower column in 2019. LCHC, liver cancer due to hepatitis C. **Supplementary figure 14.** The death overall rate of caused by LCAL by sex, age groups, and attributable risks. Death rate due to LCAL in both sexes, male, and female were (A), (B), and (C), respectively. The upper column in each group is data in 1990 and the lower column in 2019. LCAL, liver cancer due to alcohol use. **Supplementary figure 15.** The death overall rate of caused by LCNA by sex, age groups, and attributable risks. Death rate due to LCNA in both sexes, male, and female were (A), (B), and (C), respectively. The upper column in each group is data in 1990 and the lower column in 2019. LCNA, liver cancer due to non-alcoholic steatohepatitis.

**Additional file 2:** **Supplementary table 1.** The number and age-standardized rate of death due to liver cancer at national level and both sexes in 1990 and 2019, and EAPCs and the percentage change in number from 1990 to 2019. **Supplementary table 2.** The percentage change in number and the EAPCs of death attribute to liver cancer caused by specific etiologies in global, sexes, SDI areas and geographic regions from 1990 to 2019. **Supplementary table 3.** The percentage change in number and the EAPCs of death due to liver cancer caused by specific etiologies at national level from 1990 to 2019. **Supplementary table 4.** The percentage change in number and the EAPCs of death attribute to liver cancer caused by specific etiologies in global, sexes, SDI areas and geographic regions from 1990 to 2019. **Supplementary table 5.** The percentage change in number and the EAPCs of death due to liver cancer caused by specific etiologies at national level  from 1990 to 2019.

## Data Availability

Data on liver cancer cases and mortality from GBD study 2019 by age, sex, region, country, etiology and attributable risks were obtained using the Global Health Data Exchange (GHDx) query tool (http://ghdx.healthdata.org/gbd-results-tool). Data on the Human Development Index (HDI) was also acquired from the United Nations Development Program (http://hdr.undp.org/en/data). The original contributions presented in the study are included in the article/Supplementary Material, further inquiries can be directed to the corresponding author.

## Abbreviations

LC: Liver cancer
LCHB: Liver cancer due to hepatitis B
LCHC: Liver cancer due to hepatitis C
LCAL: Liver cancer due to alcohol consumption
LCNA: Liver cancer due to non-alcoholic steatohepatitis
GBD: Global Burden of Disease
ASDR: Age-standardized death rate
UI: Uncertainty interval
CI: Confidence interval
EAPC: Estimated annual percentage change
GHDx: Global Health Data Exchange
SDI: Socio-demographic index
