# Supplementary material for: The trends in death of primary liver cancer caused by specific etiologies worldwide: results from the Global Burden of Disease Study 2019 and implications for liver cancer management
Source: BMC Cancer. 2023 Jun 28;23:598. doi: 10.1186/s12885-023-11038-3 (PMC10303795; doi:10.1186/s12885-023-11038-3)
Supplement: Supplementary file 1 — Additional file 1: Supplementary figure 1. The relationship between EAPCs and ASDR in 1990 at the national level. EAPCs of death due to LCHB (A), LCHC (B), LCAL (B), and LCNA (D) had negative associations with the corresponding ASDR in 1990. The association was calculated with Pearson correlation analysis. The size of circle increases with the corresponding death number in 1990. LCHB, liver cancer due to hepatitis B; LCHC, liver cancer due to hepatitis C; LCAL, liver cancer due to alcohol consumption; LCNA, liver cancer due to non-alcoholic steatohepatitis; EAPCs, estimated annual percentage changes; ASDR, age-standardized death rate. Supplementary figure 2. The relationship between EAPCs and HDI in 2019 at the national level. EAPCs of death due to LCHB (A), LCHC (B), LCAL (B), and LCNA (D) had positive associations with HDI in 2019. The association was calculated with Pearson correlation analysis. The size of circle increases with the corresponding death numbers in 2019. LCHB, liver cancer due to hepatitis B; LCHC, liver cancer due to hepatitis C; LCAL, liver cancer due to alcohol consumption; LCNA, liver cancer due to non-alcoholic steatohepatitis; EAPCs, estimated annual percentage changes; HDI, human development index. Supplementary figure 3. The distribution of death number of LCHB in age groups, SDI areas, and geographic regions from 1990 to 2019. (A) the death number of LCHB in age groups; (B) the ASDR of LCHB in SDI areas; (C) the ASDR of LCHB in geographical regions. LCHB, liver cancer due to hepatitis B; ASDR, age-standardized death rate; SDI, sociodemographic index. Supplementary figure 4. The distribution of percentage changes in number and EAPCs of death caused by LCHB at the national level from 1990 to 2019. (A) The ASDR of LCHB in 2019; (B) The percentage changes in death number of LCHB; (C) EAPCs of death due to LCHB. Countries/territories with an extreme value were annotated. LCHB, liver cancer due to hepatitis B; ASDR, age-standardized death rate; [file 12885_2023_11038_MOESM1_ESM.doc]

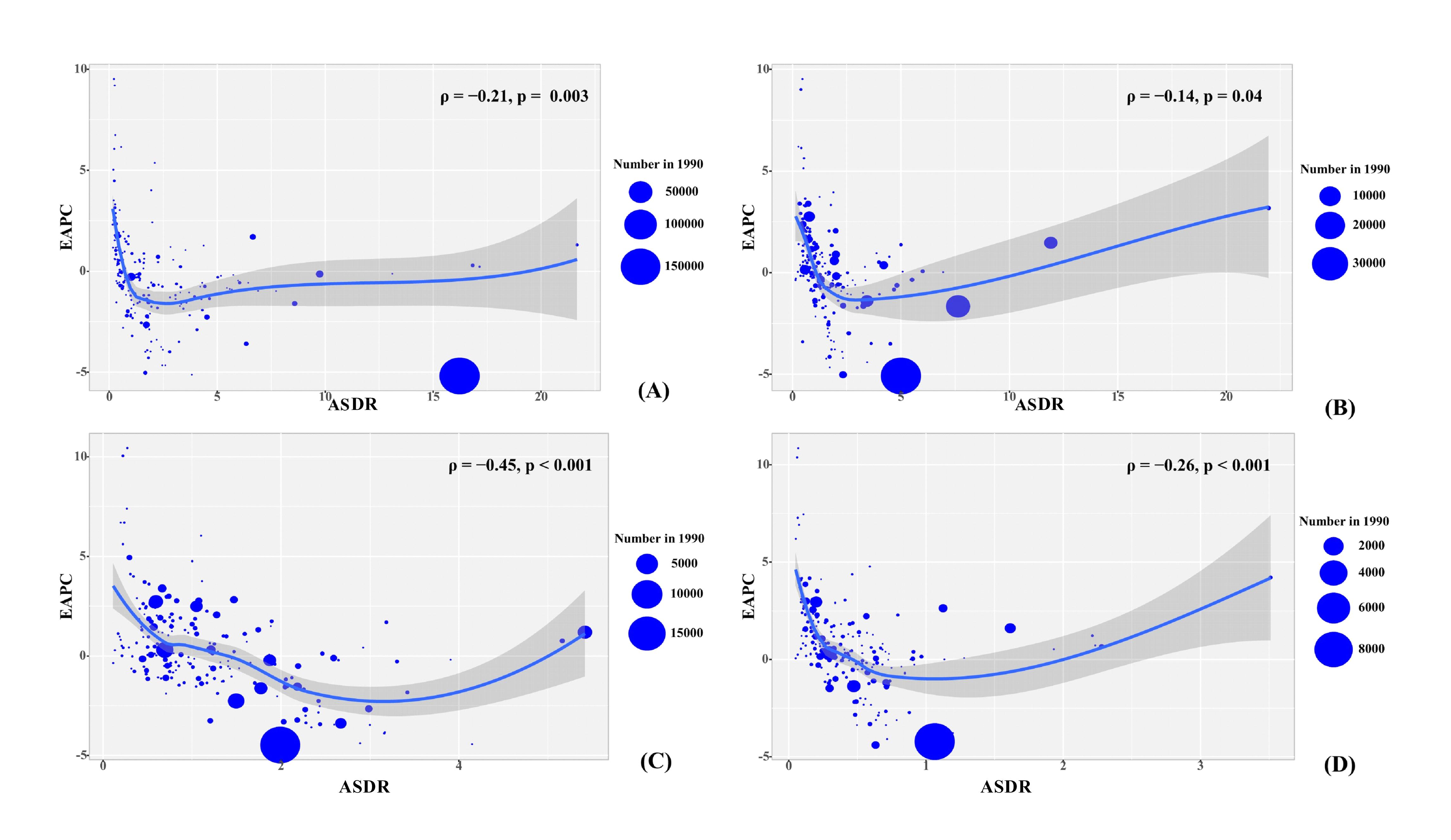


**Supplementary figure 1**. The relationship between EAPCs and ASDR in 1990 at the national level. EAPCs of death due to LCHB (A), LCHC (B), LCAL (B), and LCNA (D) had negative associations with the corresponding ASDR in 1990. The association was calculated with Pearson correlation analysis. The size of circle increases with the corresponding death number in 1990. LCHB, liver cancer due to hepatitis B; LCHC, liver cancer due to hepatitis C; LCAL, liver cancer due to alcohol consumption; LCNA, liver cancer due to non-alcoholic steatohepatitis; EAPCs, estimated annual percentage changes; ASDR, age-standardized death rate.


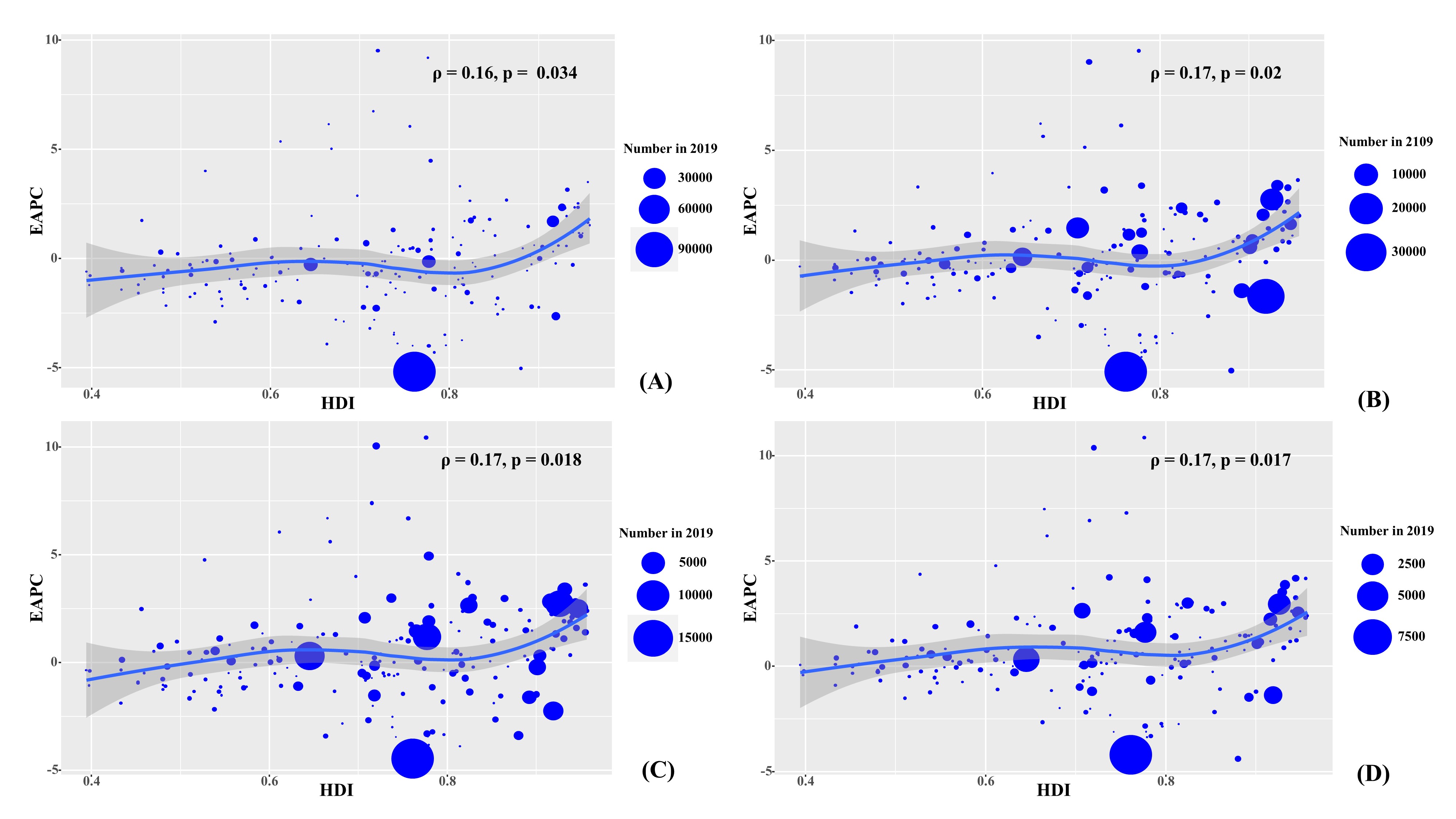


**Supplementary figure 2**. The relationship between EAPCs and HDI in 2019 at the national level. EAPCs of death due to LCHB (A), LCHC (B), LCAL (B), and LCNA (D) had positive associations with HDI in 2019. The association was calculated with Pearson correlation analysis. The size of circle increases with the corresponding death numbers in 2019. LCHB, liver cancer due to hepatitis B; LCHC, liver cancer due to hepatitis C; LCAL, liver cancer due to alcohol consumption; LCNA, liver cancer due to non-alcoholic steatohepatitis; EAPCs, estimated annual percentage changes; HDI, human development index.

**
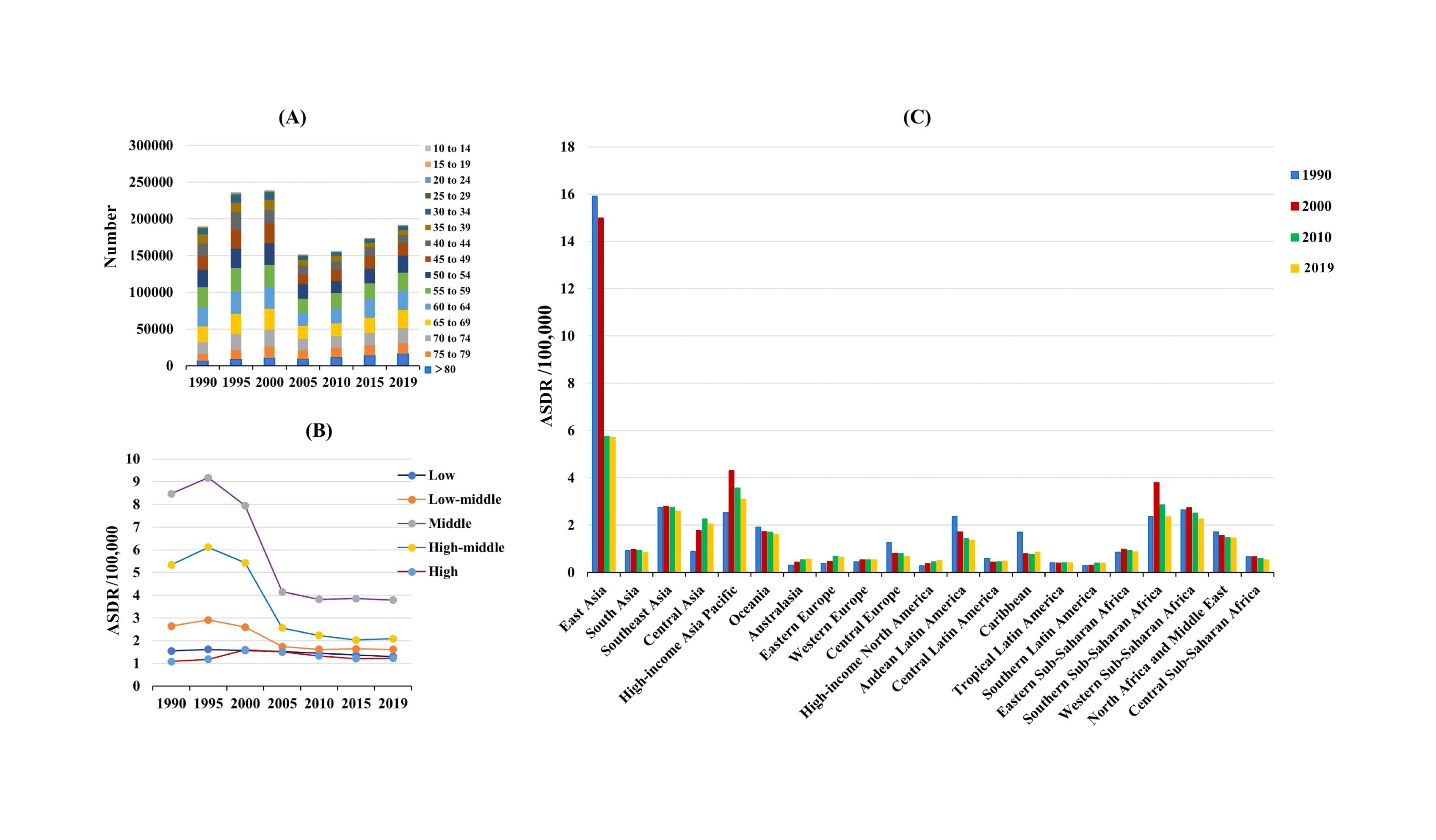
**

**Supplementary figure 3**. The distribution of death number of LCHB in age groups, SDI areas, and geographic regions from 1990 to 2019. (A) the death number of LCHB in age groups; (B) the ASDR of LCHB in SDI areas; (C) the ASDR of LCHB in geographical regions. LCHB, liver cancer due to hepatitis B; ASDR, age-standardized death rate; SDI, sociodemographic index.


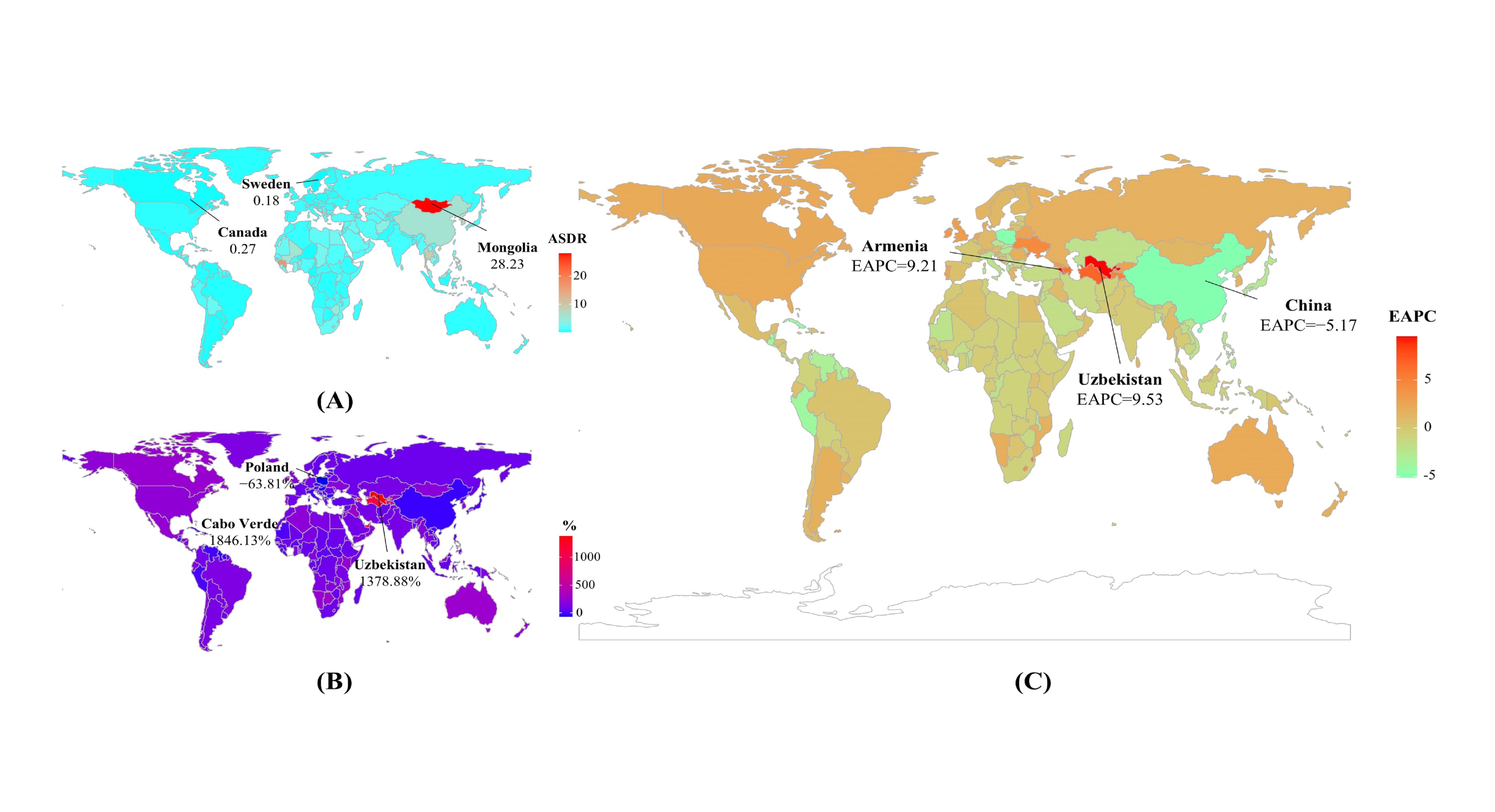


**Supplementary figure 4**. The distribution of percentage changes in number and EAPCs of death caused by LCHB at the national level from 1990 to 2019. (A) The ASDR of LCHB in 2019; (B) The percentage changes in death number of LCHB; (C) EAPCs of death due to LCHB. Countries/territories with an extreme value were annotated. LCHB, liver cancer due to hepatitis B; ASDR, age-standardized death rate; EAPC, estimated annual percentage change.

**
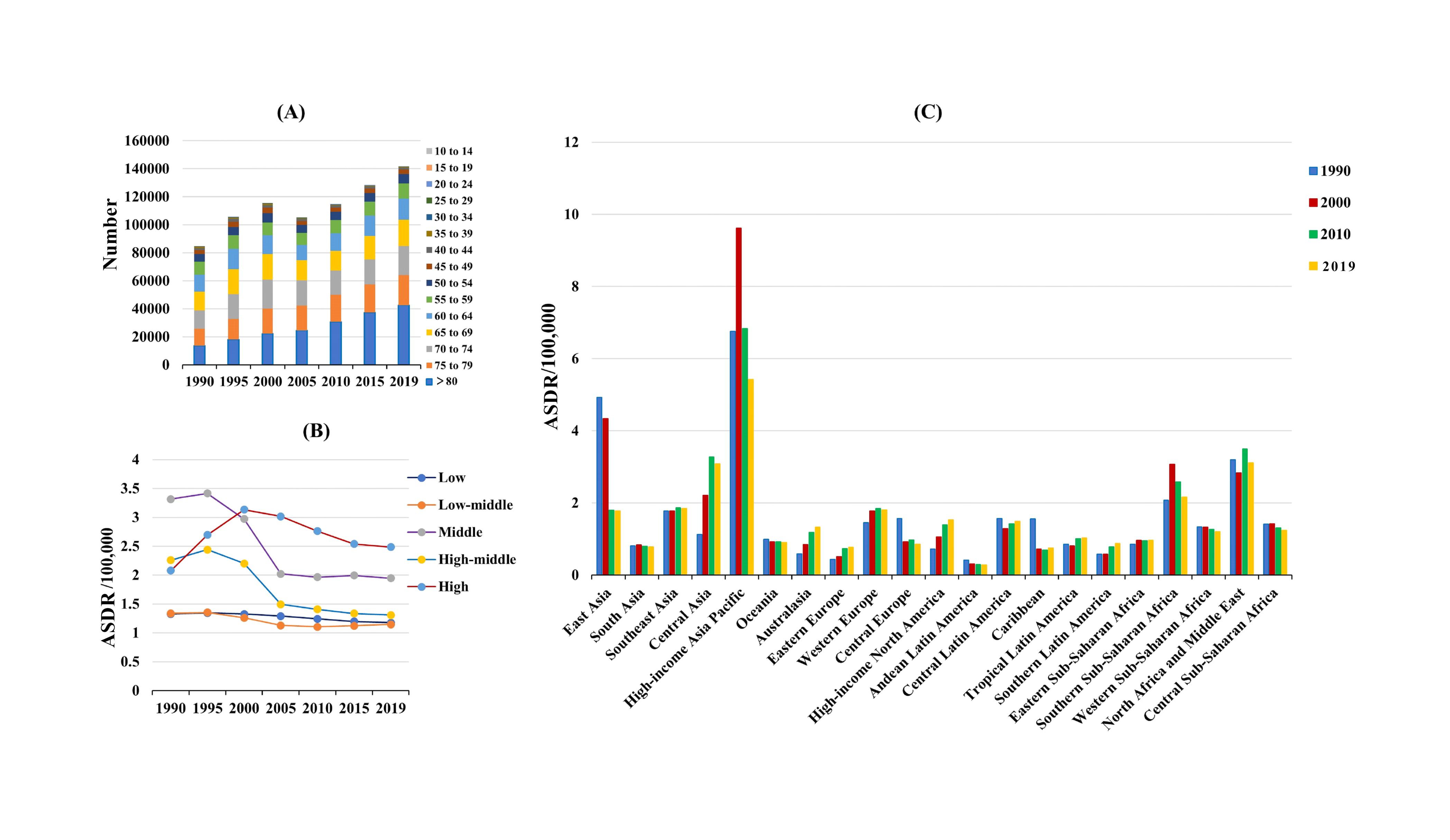
**

**Supplementary figure 5**. The distribution of death number of LCHC in age groups, SDI areas, and geographic regions from 1990 to 2019. (A) the death number of LCHC in age groups; (B) the ASDR of LCHC in SDI areas; (C) the ASDR of LCHC in geographical regions. LCHC, liver cancer due to hepatitis C; ASDR, age-standardized death rate; SDI, sociodemographic index.

**
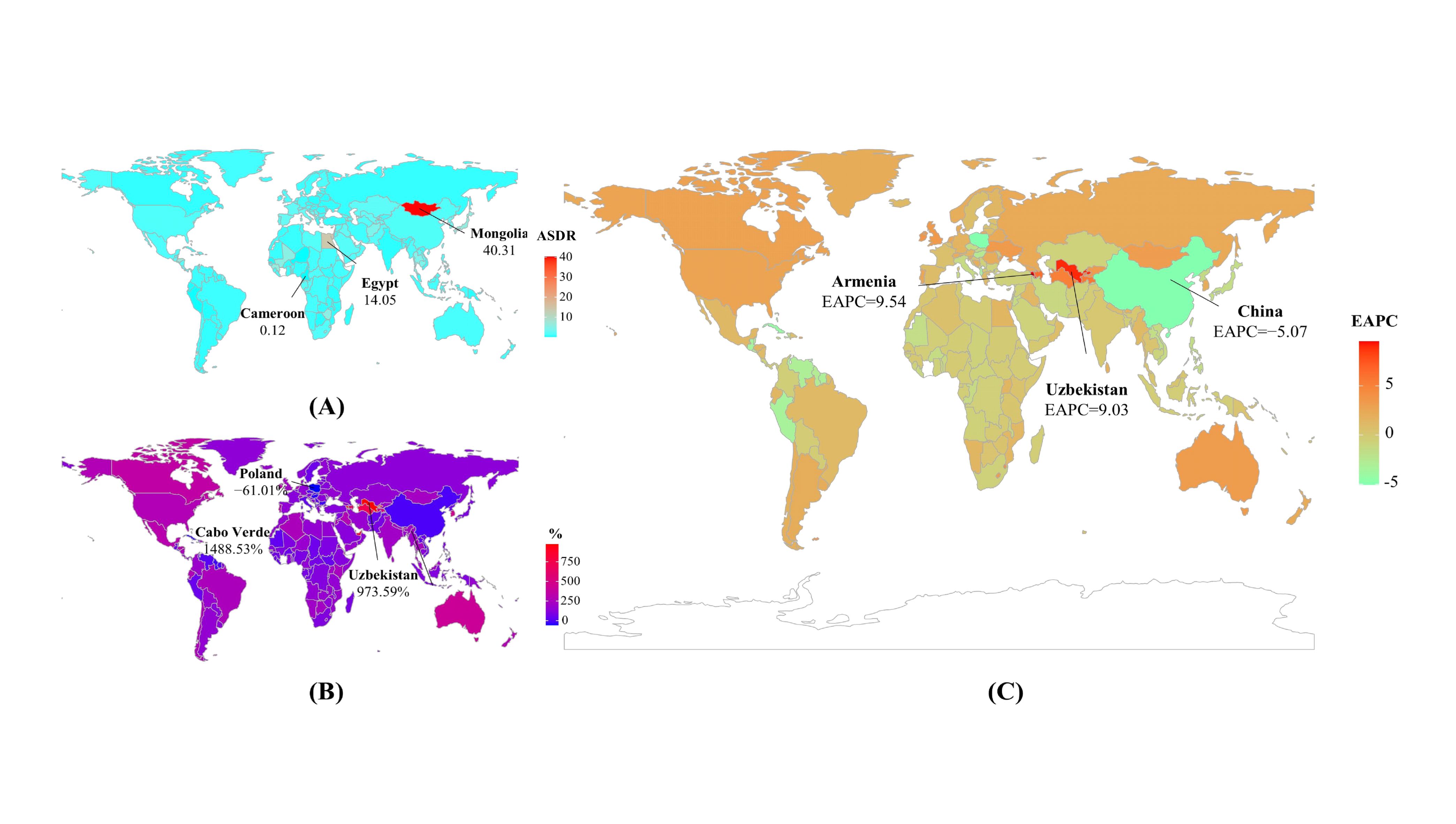
**

**Supplementary figure 6**. The distribution of percentage changes in number and EAPCs of death caused by LCHC at the national level from 1990 to 2019. (A) The ASDR of LCHC in 2019; (B) The percentage changes in death number of LCHC; (C) EAPCs of death due to LCHC. Countries/territories with an extreme value were annotated. LCHC, liver cancer due to hepatitis C; ASDR, age-standardized death rate; EAPC, estimated annual percentage change.

**
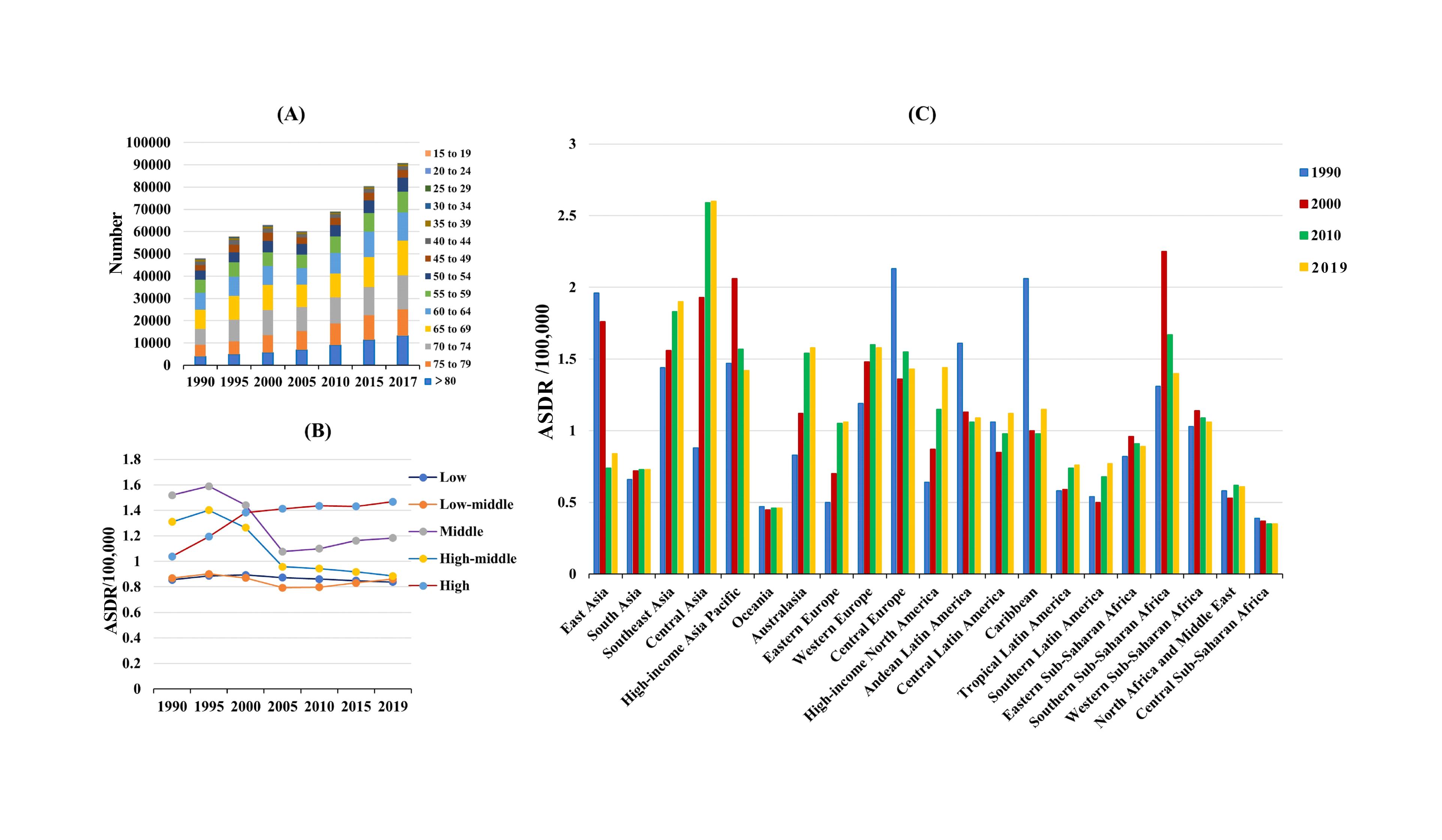
**

**Supplementary figure 7**. The distribution of death number of LCAL in age groups, SDI areas, and geographic regions from 1990 to 2019. (A) the death number of LCAL in age groups; (B) the ASDR of LCAL in SDI areas; (C) the ASDR of LCAL in geographical regions. LCAL, liver cancer due to alcohol use; ASDR, age-standardized death rate; SDI, sociodemographic index.

**
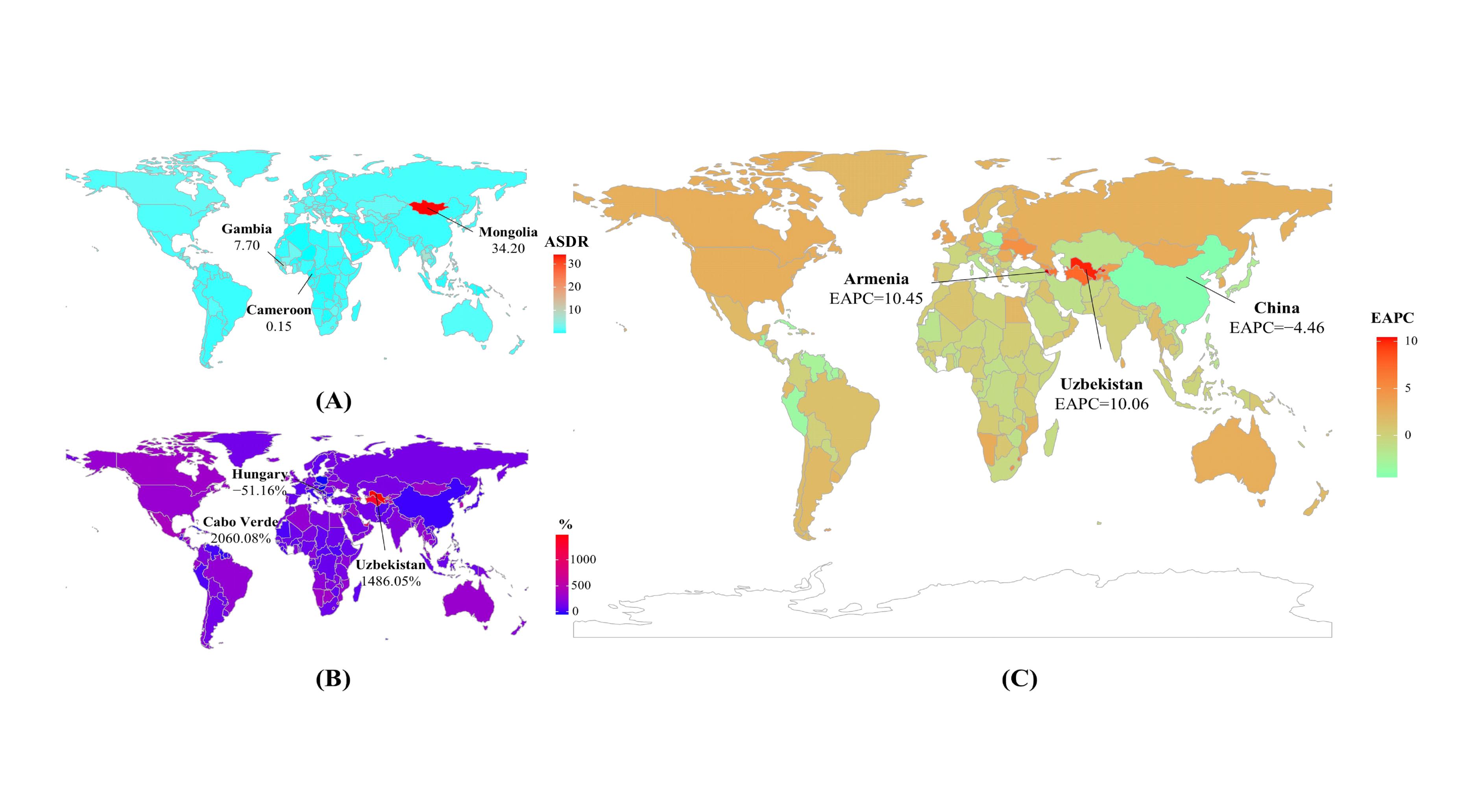
**

**Supplementary figure 8**. The distribution of percentage changes in number and EAPCs of death caused by LCAL at the national level from 1990 to 2019. (A) The ASDR of LCAL; (B) The percentage changes in number of death due to LCAL; (C) EAPCs of death due to LCAL. Countries/territories with an extreme value were annotated. LCAL, liver cancer due to alcohol use; EAPC, estimated annual percentage change.

**
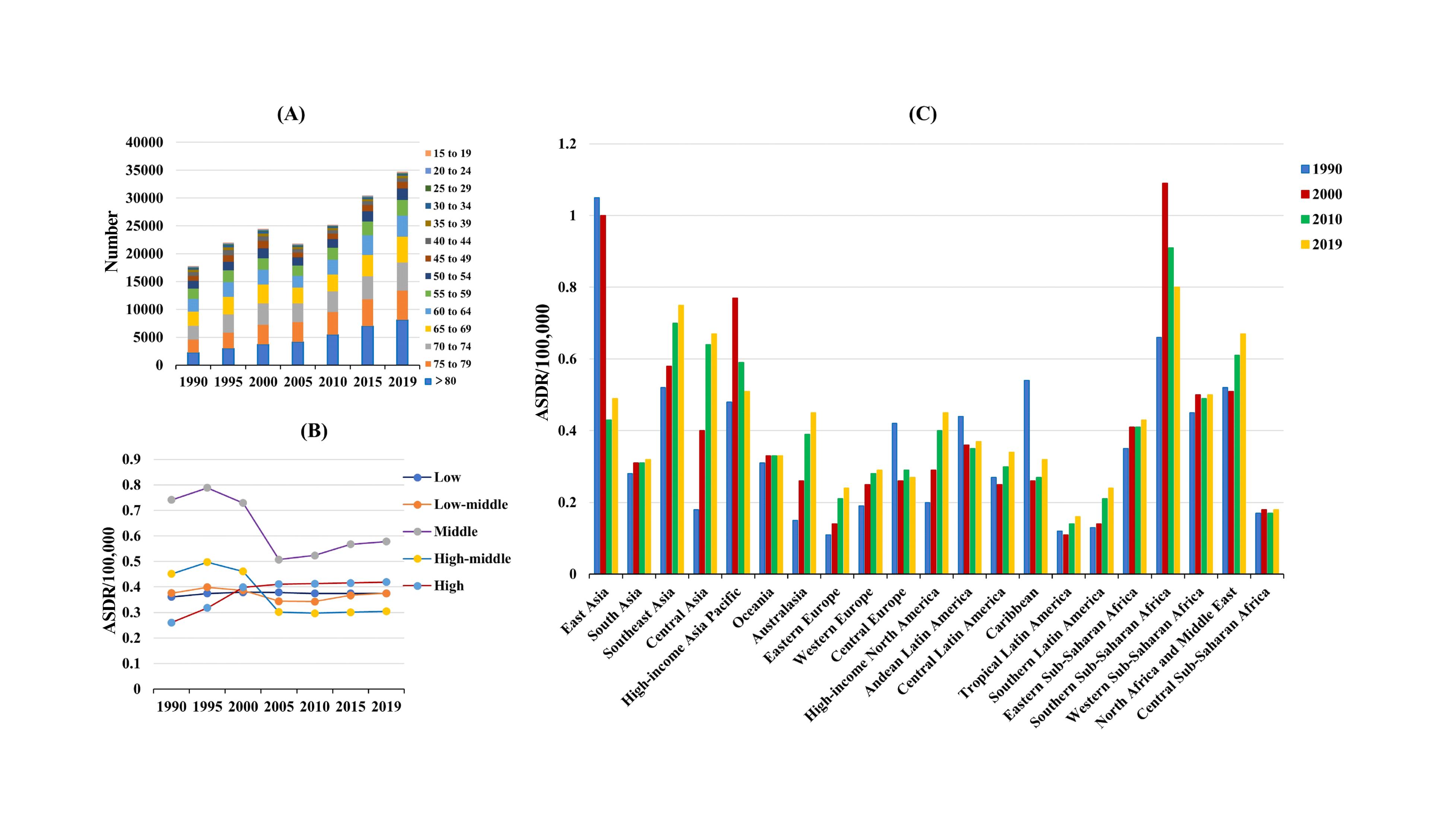
**

**Supplementary figure 9**. The distribution of death number of LCNA in age groups, SDI areas, and geographic regions from 1990 to 2019. (A) the death number of LCNA in age groups; (B) the death number of LCNA in SDI areas; (C) the death number of LCNA in geographical regions. LCNA, liver cancer due to non-alcoholic steatohepatitis; ASDR, age-standardized death rate; SDI, sociodemographic index.

**
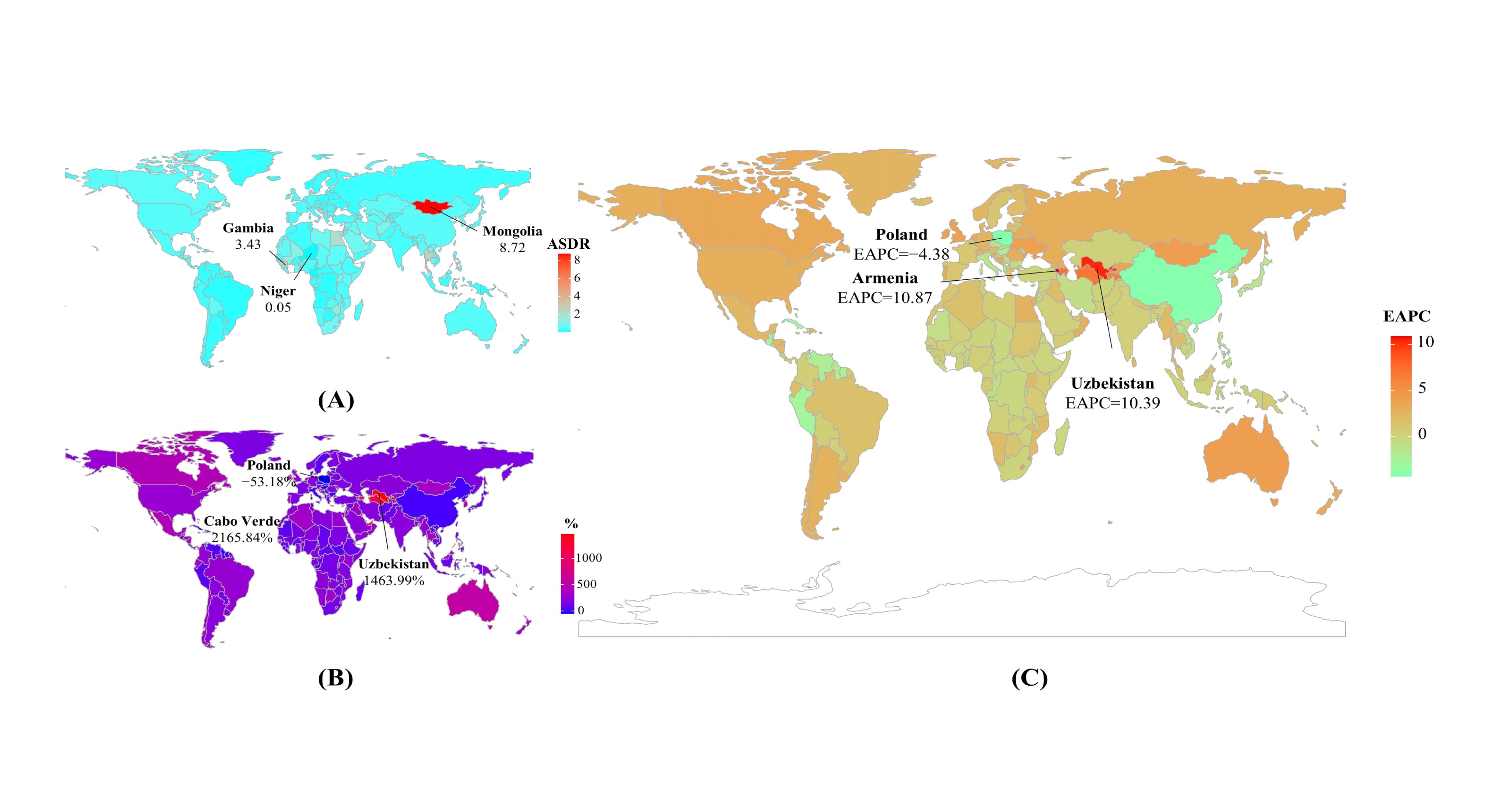
**

**Supplementary figure 10**. The distribution of percentage changes in number and EAPCs of death caused by LCNA at the national level from 1990 to 2019. (A) The ASDR of LCNA; (B) The percentage changes in number of death due to LCNA; (C) EAPCs of death due to LCNA. Countries/territories with an extreme value were annotated. LCNA, liver cancer due to non-alcoholic steatohepatitis; EAPC, estimated annual percentage change.

**
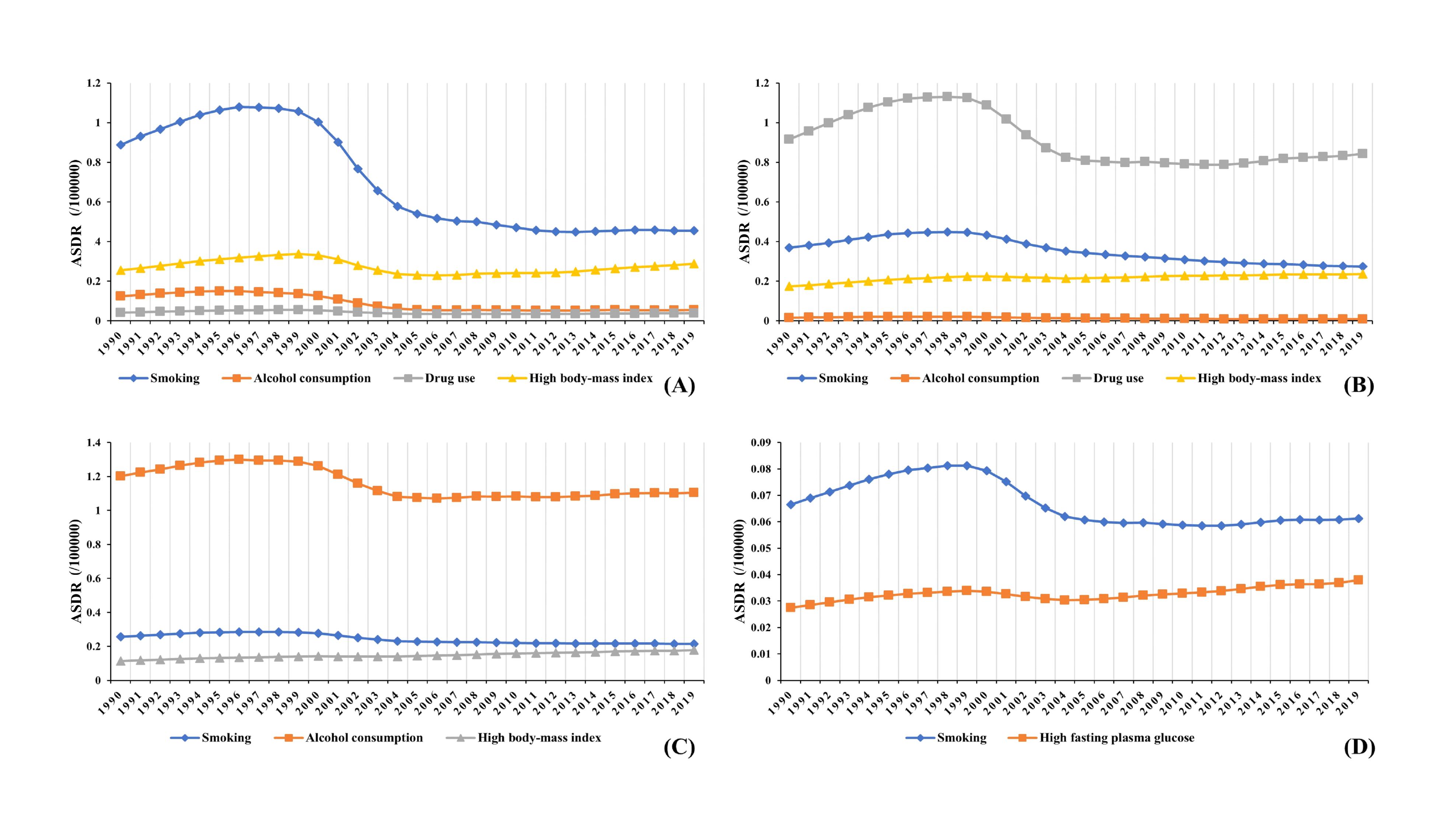
**

**Supplementary figure 11**. The distribution of ASDR of LCHB, LCHC, LCAL, and LCNA due to attributable risks from 1990 to 2019. The ASDR of LCHB, LCHC, LCAL, and LCNA due to attributable risks were (A), (B), (C), and (D), respectively. LCHB, liver cancer due to hepatitis B; LCHC, liver cancer due to hepatitis C; LCAL, liver cancer due to alcohol use; LCNA, liver cancer due to non-alcoholic steatohepatitis; ASDR, age-standardized death rate.


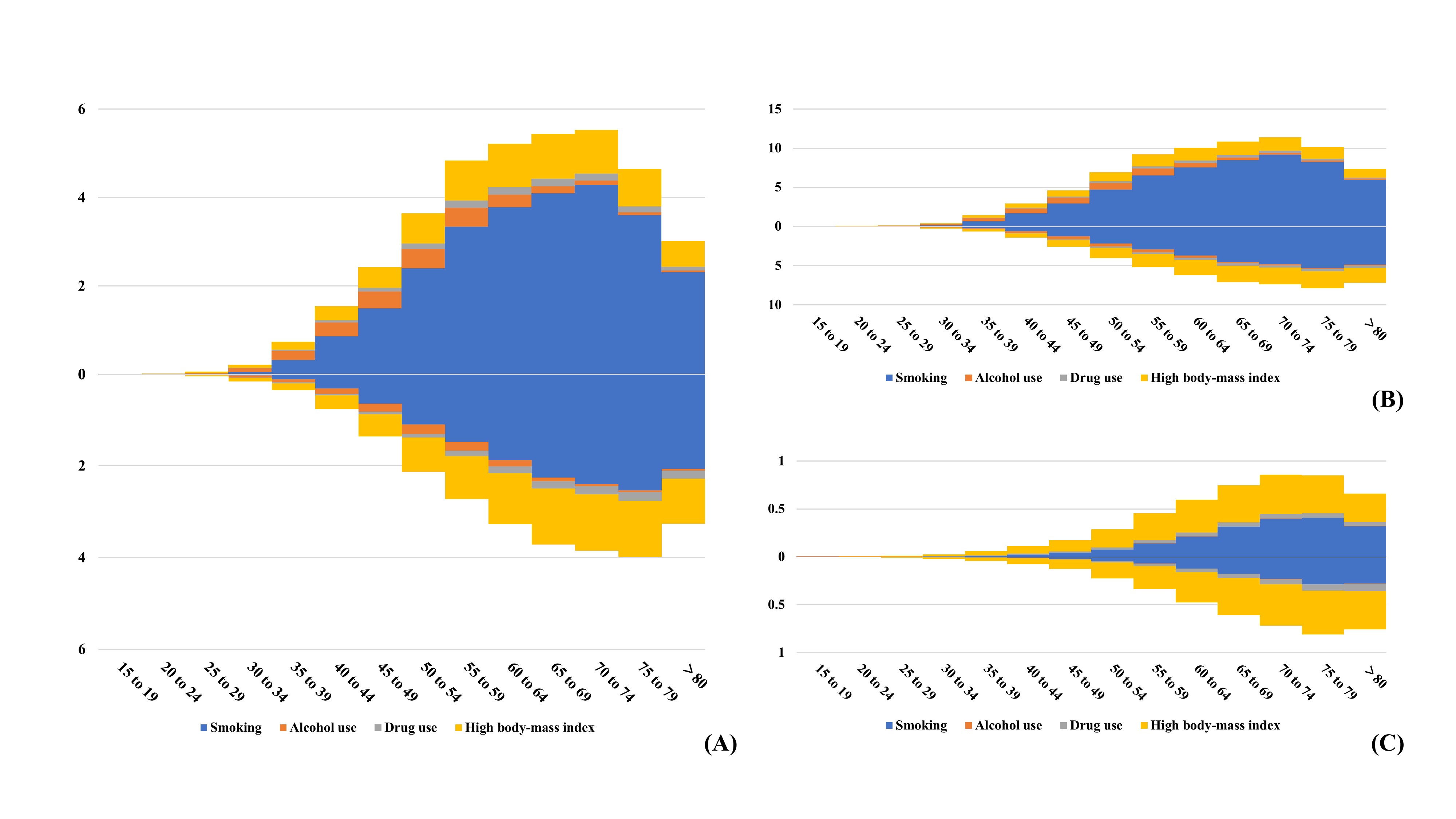


**Supplementary figure 12**. The overall death rate of LCHB by sex, age groups, and attributable risks. Death rate due to LCHB in both sexes, male, and female were (A), (B), and (C), respectively. The upper column in each group is data in 1990 and the lower column in 2019. LCHB, liver cancer due to hepatitis B.


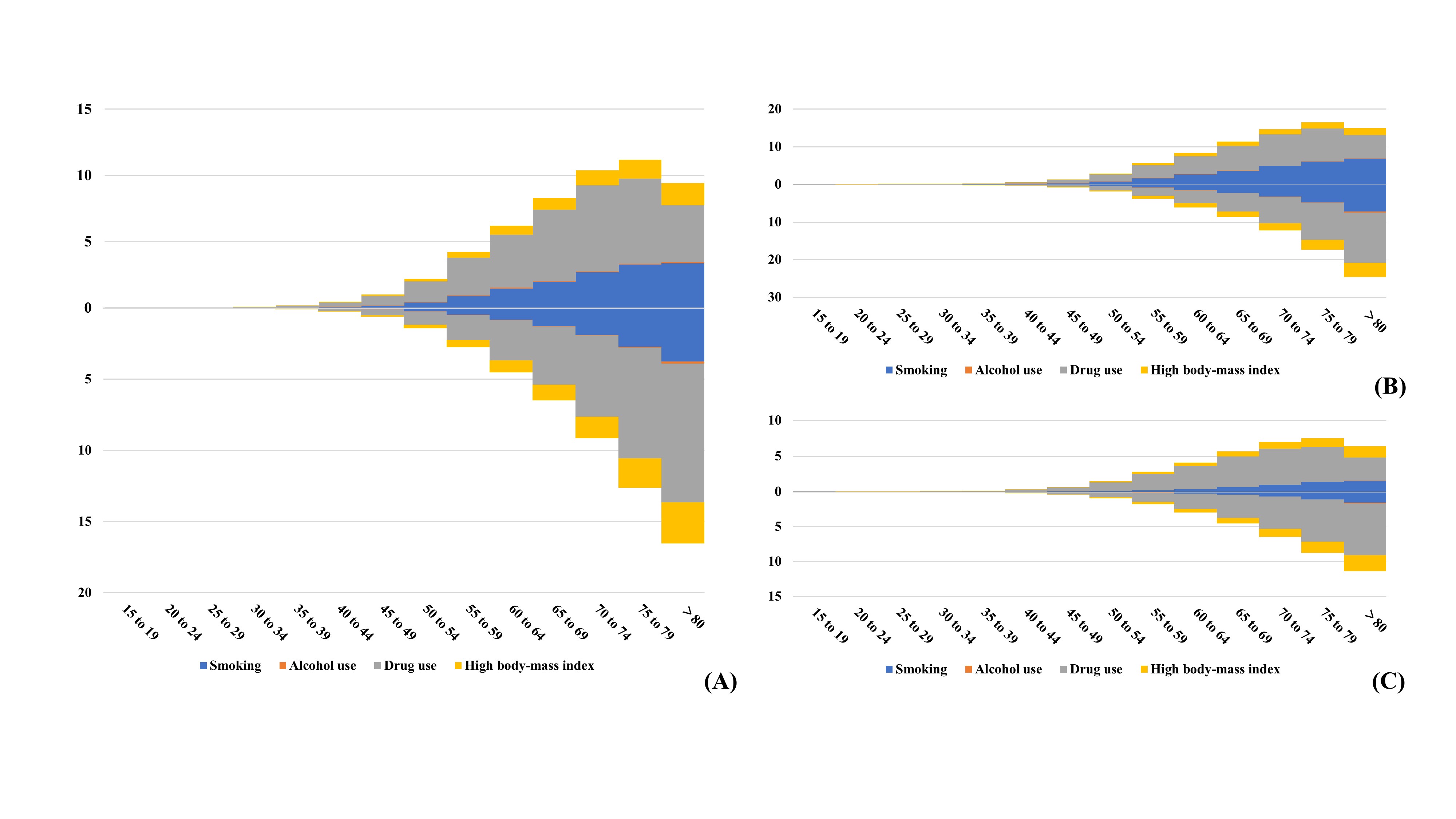


**Supplementary figure 13**. The death overall rate of caused by LCHC by sex, age groups, and attributable risks. Death rate due to LCHC in both sexes, male, and female were (A), (B), and (C), respectively. The upper column in each group is data in 1990 and the lower column in 2019. LCHC, liver cancer due to hepatitis C.


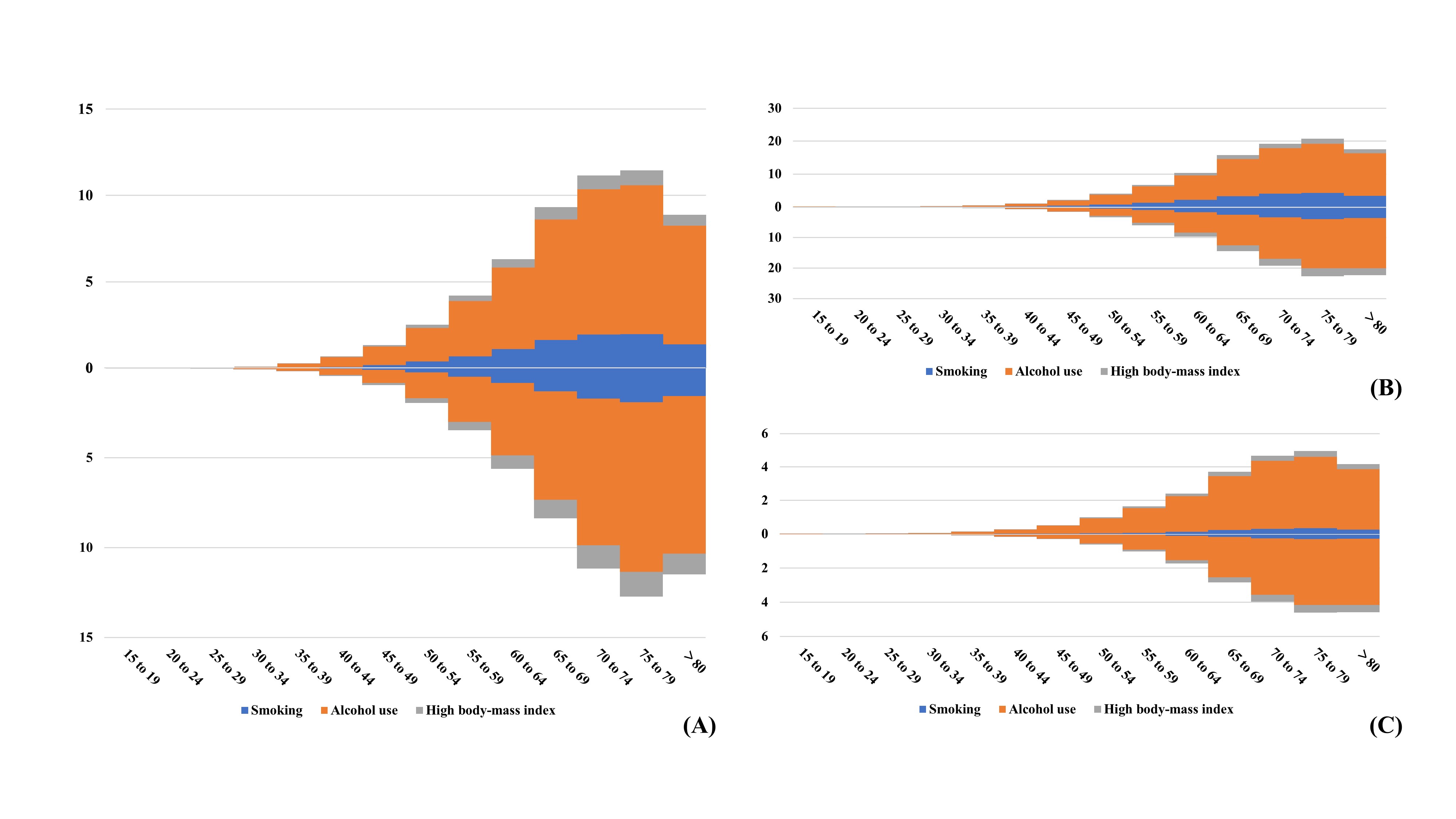


**Supplementary figure 14**. The death overall rate of caused by LCAL by sex, age groups, and attributable risks. Death rate due to LCAL in both sexes, male, and female were (A), (B), and (C), respectively. The upper column in each group is data in 1990 and the lower column in 2019. LCAL, liver cancer due to alcohol use.


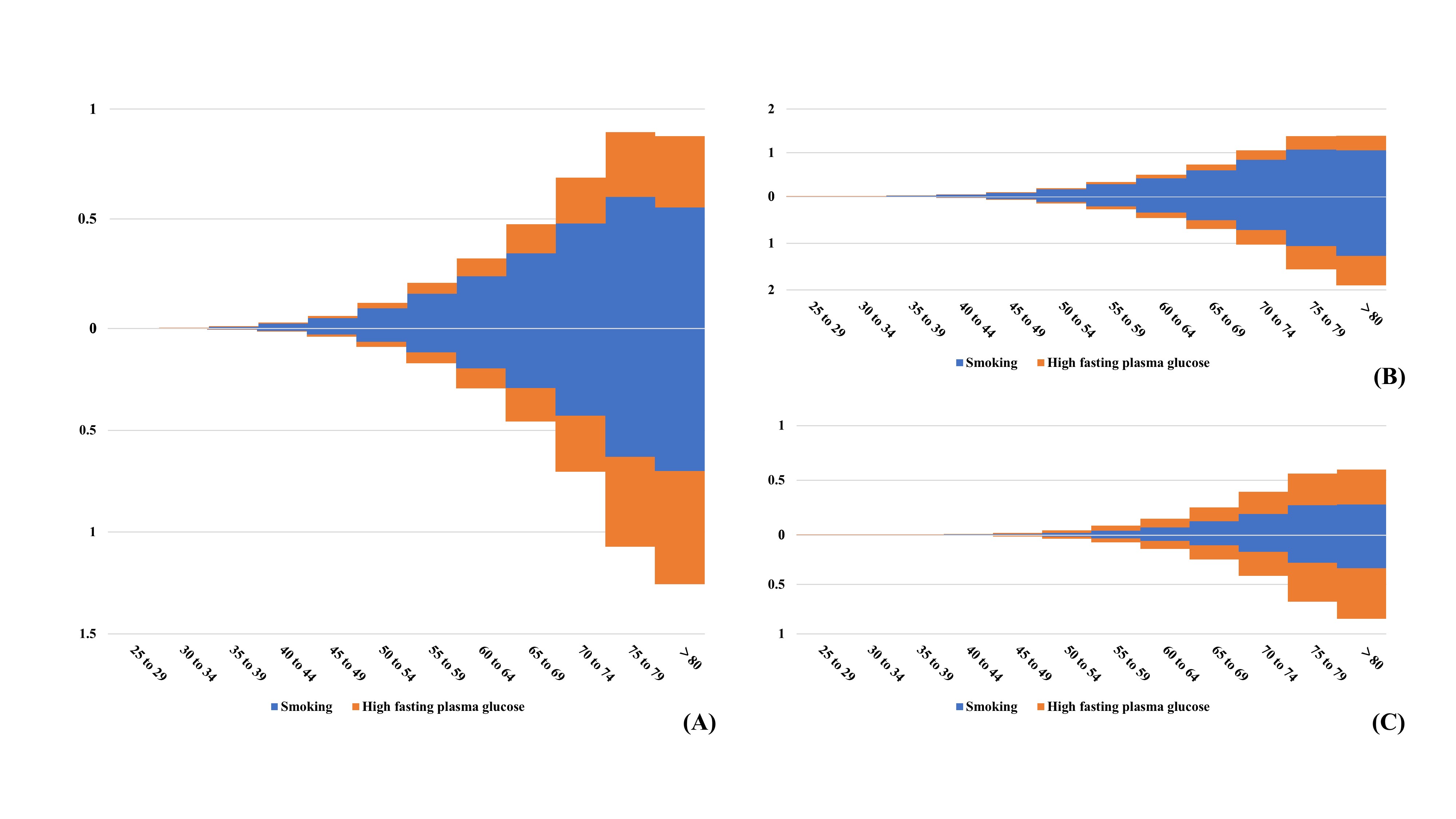


**Supplementary figure 15**. The death overall rate of caused by LCNA by sex, age groups, and attributable risks. Death rate due to LCNA in both sexes, male, and female were (A), (B), and (C), respectively. The upper column in each group is data in 1990 and the lower column in 2019. LCNA, liver cancer due to non-alcoholic steatohepatitis.
