# Supplementary material for: The trends in death of primary liver cancer caused by specific etiologies worldwide: results from the Global Burden of Disease Study 2019 and implications for liver cancer management
Source: BMC Cancer. 2023 Jun 28;23:598. doi: 10.1186/s12885-023-11038-3 (PMC10303795; doi:10.1186/s12885-023-11038-3)
Supplement: Supplementary file 2 — Additional file 2: Supplementary table 1. The number and age-standardized rate of death due to liver cancer at national level and both sexes in 1990 and 2019, and EAPCs and the percentage change in number from 1990 to 2019. Supplementary table 2. The percentage change in number and the EAPCs of death attribute to liver cancer caused by specific etiologies in global, sexes, SDI areas and geographic regions from 1990 to 2019. Supplementary table 3. The percentage change in number and the EAPCs of death due to liver cancer caused by specific etiologies at national level from 1990 to 2019. Supplementary table 4. The percentage change in number and the EAPCs of death attribute to liver cancer caused by specific etiologies in global, sexes, SDI areas and geographic regions from 1990 to 2019. Supplementary table 5. The percentage change in number and the EAPCs of death due to liver cancer caused by specific etiologies at national level from 1990 to 2019. [file 12885_2023_11038_MOESM2_ESM.doc]

**Supplementary table 1.** The number and age-standardized rate of death due to liver cancer at national level and both sexes in 1990 and 2019, and EAPCs and the percentage change in number from 1990 to 2019.

|  | **1990** | | **2019** | | **1990-2019** | |
| --- | --- | --- | --- | --- | --- | --- |
| **Characteristics** | Number  ×10^3^ (95% UI) | ASDR per 100k  (95% UI) | Number  ×10^3^ (95% UI) | ASDR per 100k  (95% UI) | Changes in number (%) | EAPC  (95%CI) |
| Afghanistan | 0.85(0.66-1.09) | 11.97(28.5-46.45) | 1.38(1.04-1.79) | 10.27(7.98-12.93) | 61.71 | −0.58(−0.68-−0.49) |
| Albania | 0.23(0.22-0.25) | 11.62(30.59-51.95) | 0.29(0.22-0.39) | 6.84(5.04-9.09) | 26.26 | −2.85(−3.40-−2.30) |
| Algeria | 0.21(0.17-0.26) | 1.81(18.43-30.78) | 0.69(0.53-0.87) | 2.20(1.72-2.78) | 229.12 | 0.69(0.54-0.85) |
| American Samoa | 0 | 5.69(29.84-49.83) | 0 | 7.02(5.85-8.45) | 155.97 | 1.11(0.89-1.32) |
| Andorra | 0.01(0-0.01) | 9.38(2.05-2.81) | 0.01(0.01-0.02) | 9.44(7.14-12.24) | 164.74 | 0.06(−0.02-0.15) |
| Angola | 0.12(0.09-0.15) | 2.75(57.3-99.50) | 0.30(0.23-0.39) | 2.59(2.09-3.23) | 158.12 | −0.35(−0.43-−0.27) |
| Antigua and Barbuda | 0 | 8.11(34.44-58.62) | 0 | 2.85(2.44-3.31) | −36.24 | −3.83(−4.8-−2.85) |
| Argentina | 0.48(0.42-0.55) | 1.51(28.73-47.96) | 1.17(1.09-1.27) | 2.15(2.00-2.33) | 143.72 | 1.98(1.75-2.21) |
| Armenia | 0.03(0.03-0.03) | 1.13(31.84-53.96) | 0.29(0.24-0.34) | 7.07(5.88-8.38) | 887.16 | 9.56(8.02-11.12) |
| Australia | 0.38(0.37-0.40) | 1.96(3.18-4.17) | 1.73(1.57-1.89) | 4.20(3.83-4.59) | 351.58 | 3.01(2.79-3.23) |
| Austria | 0.39(0.37-0.40) | 3.30(4.50-5.55) | 0.80(0.71-0.89) | 4.50(4.04-4.99) | 106.45 | 1.32(1.06-1.58) |
| Azerbaijan | 0.05(0.05-0.06) | 1.02(31.87-53.10) | 0.36(0.28-0.48) | 4.37(3.31-5.94) | 617.92 | 6.16(4.15-8.2) |
| Bahamas | 0.01(0.01-0.01) | 8.47(36.31-61.74) | 0.01(0.01-0.02) | 3.21(2.66-3.96) | −6.63 | −3.58(−4.43-−2.72) |
| Bahrain | 0.01(0.01-0.01) | 6.11(2.65-3.52) | 0.04(0.03-0.05) | 5.23(4.12-6.53) | 330.28 | −1.34(−1.76-−0.92) |
| Bangladesh | 1.74(1.40-2.12) | 3.24(26.97-46.18) | 3.52(2.77-4.40) | 2.75(2.17-3.42) | 102.34 | −0.94(−1.09-−0.79) |
| Barbados | 0.01(0.01-0.01) | 2.91(35.72-60.45) | 0.01(0.01-0.02) | 2.74(2.29-3.27) | 54.89 | −0.40(−0.63-−0.16) |
| Belarus | 0.17(0.16-0.18) | 1.31(6.83-9.33) | 0.38(0.28-0.50) | 2.41(1.76-3.24) | 125.80 | 2.96(2.30-3.64) |
| Belgium | 0.46(0.43-0.48) | 2.93(3.91-5.13) | 0.87(0.79-0.94) | 3.78(3.45-4.12) | 89.39 | 0.77(0.41-1.12) |
| Belize | 0.01(0.01-0.01) | 6.33(37.46-63.69) | 0.01(0.01-0.01) | 3.21(2.75-3.70) | 44.81 | −2.51(−3.09-−1.93) |
| Benin | 0.14(0.12-0.18) | 6.96(41.5-69.34) | 0.26(0.19-0.34) | 5.12(3.89-6.79) | 77.12 | −1.37(−1.52-−1.22) |
| Bermuda | 0(0-0.01) | 7.49(2.29-3.27) | 0 | 2.05(1.70-2.51) | −41.47 | −4.40(−5.45-−3.34) |
| Bhutan | 0.01(0-0.01) | 2.29(28.06-46.73) | 0.02(0.01-0.03) | 3.27(2.29-4.61) | 199.74 | 1.34(1.24-1.43) |
| Bolivia | 0.17(0.13-0.22) | 5.29(38.92-65.21) | 0.43(0.32-0.55) | 5.03(3.77-6.43) | 152.64 | −0.27(−0.36-−0.17) |
| Bosnia and Herzegovina | 0.23(0.22-0.25) | 5.78(2.90-4.00) | 0.48(0.38-0.60) | 8.01(6.40-10.00) | 106.30 | 1.62(1.28-1.97) |
| Botswana | 0.01(0-0.01) | 1.03(51.23-88.44) | 0.02(0.02-0.03) | 1.53(1.13-2.02) | 273.72 | 0.56(−0.22-1.35) |
| Brazil | 1.83(1.75-1.89) | 2.09(40.73-68.51) | 5.82(5.43-6.13) | 2.50(2.33-2.64) | 218.15 | 1.10(0.95-1.24) |
| Brunei Darussalam | 0.01(0.01-0.01) | 10.13(4.76-6.22) | 0.03(0.03-0.04) | 11.53(9.82-13.38) | 268.89 | 1.15(0.65-1.65) |
| Bulgaria | 0.81(0.76-0.86) | 6.43(4.08-5.23) | 0.65(0.52-0.79) | 4.62(3.68-5.71) | −19.92 | −0.55(−1.08-−0.02) |
| Burkina Faso | 0.15(0.12-0.19) | 3.25(41.27-70.49) | 0.25(0.19-0.32) | 2.37(1.80-2.96) | 61.84 | −1.27(−1.46-−1.07) |
| Burundi | 0.10(0.07-0.13) | 3.94(64.18-108.95) | 0.14(0.10-0.22) | 3.11(2.17-4.80) | 48.13 | −1.35(−1.53-−1.18) |
| Cabo Verde | 0 | 1.17(37.49-64.94) | 0.05(0.04-0.06) | 12.34(10.2-14.87) | 1786.75 | 6.41(4.17-8.70) |
| Cambodia | 0.68(0.52-0.90) | 11.62(23.12-37.91) | 1.18(0.92-1.46) | 9.86(7.85-12.03) | 75.12 | −0.81(−0.91-−0.70) |
| Cameroon | 0.03(0.02-0.05) | 0.69(41.53-71.47) | 0.08(0.06-0.12) | 0.69(0.51-0.92) | 177.10 | −0.19(−0.43-0.04) |
| Canada | 0.62(0.59-0.64) | 1.92(7.17-10.11) | 2.67(2.37-2.95) | 3.91(3.48-4.32) | 331.38 | 2.84(2.63-3.04) |
| Central African Republic | 0.05(0.03-0.07) | 3.82(66.49-112.67) | 0.08(0.05-0.11) | 3.42(2.27-5.01) | 56.67 | −0.90(−1.13-−0.67) |
| Chad | 0.19(0.15-0.24) | 6.54(41.21-69.30) | 0.33(0.25-0.42) | 5.68(4.37-7.29) | 70.97 | −0.64(−0.74-−0.55) |
| Chile | 0.22(0.20-0.24) | 2.21(3.30-4.47) | 0.73(0.66-0.79) | 3.02(2.76-3.29) | 232.60 | 1.72(1.45-1.98) |
| China | 232.45(197.4-275.39) | 25.99(18.62-29.89) | 187.7(158.26-222.77) | 9.41(7.95-11.13) | −19.25 | −5.06(−5.84-−4.27) |
| Colombia | 0.52(0.49-0.55) | 3.02(2.18-3.03) | 1.40(1.07-1.79) | 2.65(2.03-3.40) | 167.21 | −0.27(−0.72-0.18) |
| Comoros | 0.01(0-0.01) | 3.31(58.34-101.49) | 0.01(0.01-0.02) | 3.01(2.12-4.65) | 91.68 | −0.50(−0.64-−0.36) |
| Congo | 0.05(0.04-0.06) | 4.28(60.94-103.81) | 0.08(0.06-0.11) | 3.14(2.33-4.24) | 66.20 | −1.41(−1.56-−1.27) |
| Cook Islands | 0 | 13.60(7.86-10.18) | 0 | 11.13(9.03-13.51) | 56.99 | −0.62(−0.74-−0.51) |
| Costa Rica | 0.10(0.09-0.11) | 5.74(35.94-60.13) | 0.27(0.21-0.34) | 5.30(4.11-6.74) | 170.00 | −0.50(−1.02-0.03) |
| Croatia | 0.20(0.19-0.22) | 3.22(3.74-4.63) | 0.31(0.24-0.39) | 3.53(2.77-4.48) | 51.87 | 0.60(0.05-1.14) |
| Cuba | 0.70(0.66-0.73) | 6.71(35.29-59.92) | 0.46(0.37-0.57) | 2.43(1.95-3.00) | −33.23 | −3.79(−5.04-−2.53) |
| Cyprus | 0.03(0.02-0.03) | 3.40(4.01-5.13) | 0.06(0.06-0.07) | 3.42(2.95-3.94) | 146.71 | 0.22(−0.01-0.46) |
| Czechia | 0.63(0.59-0.66) | 4.52(4.43-5.84) | 0.63(0.52-0.78) | 2.99(2.45-3.68) | 0.94 | −1.64(−1.86-−1.43) |
| Côte d'Ivoire | 0.33(0.25-0.43) | 7.93(42.6-71.87) | 0.54(0.39-0.74) | 5.06(3.77-6.83) | 62.12 | −2.35(−2.66-−2.04) |
| Democratic People's Republic of Korea | 2.53(1.94-3.3) | 14.77(21.51-35.78) | 3.31(2.5-4.33) | 10.2(7.75-13.3) | 30.87 | −1.52(−1.62-−1.41) |
| Democratic Republic of the Congo | 0.48(0.38-0.59) | 2.67(61.85-105.87) | 0.89(0.68-1.16) | 2.28(1.77-2.94) | 85.93 | −0.62(−0.69-−0.56) |
| Denmark | 0.16(0.15-0.16) | 1.91(1.75-2.32) | 0.37(0.33-0.41) | 3.25(2.93-3.57) | 138.63 | 2.19(1.95-2.43) |
| Djibouti | 0.01(0-0.01) | 3.37(56.83-98.14) | 0.02(0.01-0.03) | 3.49(2.29-5.59) | 296.77 | 0.05(−0.07-0.17) |
| Dominica | 0.01(0.01-0.01) | 9.13(38.27-65.32) | 0 | 3.39(2.76-4.16) | −54.03 | −3.66(−4.44-−2.87) |
| Dominican Republic | 0.16(0.14-0.18) | 4.16(36.21-61.00) | 0.45(0.31-0.68) | 4.92(3.40-7.23) | 184.38 | 0.80(0.29-1.31) |
| Ecuador | 0.16(0.14-0.18) | 3.03(35.91-60.97) | 0.54(0.43-0.69) | 3.71(2.97-4.73) | 233.34 | 1.11(0.78-1.44) |
| Egypt | 5.20(4.13-6.22) | 17.43(25.47-41.43) | 13.59(9.72-18.63) | 21.25(15.44-28.92) | 161.47 | 1.52(1.18-1.86) |
| El Salvador | 0.11(0.10-0.11) | 3.55(38.66-65.46) | 0.13(0.10-0.16) | 2.12(1.61-2.76) | 19.40 | −1.98(−2.65-−1.31) |
| Equatorial Guinea | 0.01(0.01-0.01) | 3.24(53.39-91.85) | 0.02(0.01-0.02) | 3.48(2.04-5.04) | 141.53 | 0.20(−0.01-0.41) |
| Eritrea | 0.04(0.02-0.06) | 3.4(70.93-119.54) | 0.09(0.06-0.13) | 3.32(2.36-4.73) | 146.33 | −0.38(−0.55-−0.20) |
| Estonia | 0.04(0.04-0.05) | 2.12(6.91-9.89) | 0.09(0.07-0.12) | 3.61(2.76-4.54) | 120.00 | 0.98(0.41-1.56) |
| Eswatini | 0.02(0.01-0.03) | 5.95(53.08-90.85) | 0.12(0.03-0.21) | 19.09(5.98-33.88) | 559.55 | 5.04(3.75-6.35) |
| Ethiopia | 0.67(0.46-0.96) | 3.13(55.86-95.60) | 1.23(0.97-1.55) | 3.02(2.40-3.84) | 83.58 | −0.31(−0.41-−0.21) |
| Fiji | 0.02(0.02-0.03) | 5.95(23.14-39.16) | 0.05(0.04-0.06) | 6.23(4.86-7.83) | 113.05 | 0.67(0.30-1.05) |
| Finland | 0.21(0.19-0.22) | 2.85(1.25-1.73) | 0.51(0.46-0.56) | 4.03(3.68-4.42) | 148.07 | 1.56(1.42-1.69) |
| France | 4.01(3.81-4.2) | 4.94(3.01-3.88) | 7.79(6.83-8.77) | 5.80(5.10-6.56) | 94.35 | 0.26(0-0.51) |
| Gabon | 0.02(0.02-0.03) | 3.59(53.30-92.91) | 0.04(0.02-0.05) | 3.54(2.33-5.01) | 72.75 | −0.25(−0.39-−0.11) |
| Gambia | 0.12(0.09-0.16) | 30.76(43.05-73.30) | 0.41(0.30-0.54) | 39.51(29.01-50.99) | 236.90 | 0.55(0.37-0.74) |
| Georgia | 0.07(0.06-0.08) | 1.20(33.32-55.01) | 0.21(0.17-0.26) | 3.63(2.97-4.42) | 197.69 | 3.07(1.91-4.24) |
| Germany | 3.08(2.84-3.31) | 2.43(3.78-4.75) | 7.74(7.11-8.36) | 4.02(3.70-4.33) | 151.28 | 2.03(1.66-2.41) |
| Ghana | 0.40(0.31-0.54) | 6.21(40.5-69.03) | 0.99(0.73-1.27) | 6.00(4.59-7.59) | 147.90 | −0.37(−0.55-−0.19) |
| Greece | 0.35(0.33-0.37) | 2.30(1.62-2.18) | 0.78(0.71-0.85) | 3.21(2.95-3.47) | 122.70 | 1.38(1.23-1.53) |
| Greenland | 0 | 4.60(9.31-13.28) | 0(0-0.01) | 6.35(5.03-7.96) | 164.06 | 1.15(1.05-1.26) |
| Grenada | 0.01(0.01-0.01) | 9.40(36.61-63.13) | 0 | 3.13(2.76-3.54) | −50.14 | −4.11(−5.03-−3.17) |
| Guam | 0 | 3.91(25.95-44.31) | 0.01(0.01-0.01) | 5.79(4.71-7.05) | 273.38 | 1.88(1.67-2.09) |
| Guatemala | 0.35(0.31-0.39) | 10.05(40.81-68.86) | 0.52(0.41-0.65) | 4.81(3.84-6.01) | 47.77 | −3.46(−4.89-−2.02) |
| Guinea | 1.08(0.89-1.29) | 31.80(35.28-59.56) | 1.94(1.35-2.51) | 34.05(23.98-44.01) | 79.87 | 0.37(0.31-0.43) |
| Guinea-Bissau | 0.04(0.03-0.05) | 8.34(44.83-74.72) | 0.05(0.03-0.06) | 6.36(4.67-8.51) | 33.54 | −1.06(−1.13-−0.98) |
| Guyana | 0.03(0.02-0.03) | 6.86(41.26-69.38) | 0.02(0.02-0.02) | 3.20(2.53-4.03) | −26.01 | −2.74(−3.53-−1.93) |
| Haiti | 0.23(0.14-0.31) | 7.11(45.34-76.18) | 0.30(0.18-0.45) | 4.42(2.67-6.66) | 31.89 | −1.76(−1.98-−1.54) |
| Honduras | 0.24(0.09-0.35) | 11.92(40.71-68.24) | 0.93(0.43-1.37) | 16.14(7.41-23.5) | 286.82 | 1.35(1.17-1.54) |
| Hungary | 1.00(0.96-1.05) | 6.82(5.03-6.73) | 0.51(0.42-0.62) | 2.65(2.18-3.23) | −49.04 | −2.56(−3.36-−1.75) |
| Iceland | 0.01(0-0.01) | 1.88(1.70-2.25) | 0.02(0.01-0.02) | 2.84(2.51-3.20) | 192.86 | 1.46(1.32-1.6) |
| India | 11.86(9.88-13.74) | 2.70(31.68-53.52) | 30.71(25.88-36.35) | 2.75(2.32-3.27) | 158.95 | 0.06(−0.05-0.17) |
| Indonesia | 2.55(2.19-2.89) | 2.62(8.34-12.43) | 4.72(3.97-5.40) | 2.45(2.08-2.76) | 84.81 | −0.36(−0.45-−0.28) |
| Iran  (Islamic Republic of) | 1.08(0.89-1.26) | 4.57(22.49-37.17) | 2.50(2.27-2.75) | 3.64(3.29-4.02) | 132.49 | −1.21(−1.84-−0.57) |
| Iraq | 0.36(0.27-0.44) | 4.58(24.58-40.52) | 1.34(1.02-1.69) | 6.07(4.72-7.47) | 273.32 | 1.22(0.79-1.65) |
| Ireland | 0.06(0.06-0.07) | 1.47(2.28-2.97) | 0.26(0.23-0.28) | 3.40(3.04-3.75) | 323.75 | 3.62(3.28-3.96) |
| Israel | 0.13(0.12-0.14) | 2.65(3.20-4.04) | 0.32(0.29-0.35) | 2.79(2.55-3.04) | 150.21 | 0.1(0-0.21) |
| Italy | 5.83(5.64-5.96) | 6.50(5.56-7.37) | 6.92(6.23-7.50) | 4.80(4.36-5.17) | 18.69 | −1.55(−1.81-−1.29) |
| Jamaica | 0.07(0.07-0.08) | 4.10(36.62-62.09) | 0.08(0.07-0.10) | 2.75(2.19-3.41) | 10.17 | −0.73(−1.63-0.18) |
| Japan | 19.99(19.31-20.46) | 11.67(2.36-3.16) | 34.51(29.61-37.43) | 8.78(7.80-9.42) | 72.69 | −1.84(−2.39-−1.29) |
| Jordan | 0.04(0.03-0.05) | 3.21(1.46-1.99) | 0.14(0.11-0.18) | 2.38(1.91-2.97) | 251.67 | −1.27(−1.40-−1.13) |
| Kazakhstan | 0.45(0.42-0.48) | 3.57(6.26-7.99) | 1.12(0.96-1.29) | 6.54(5.63-7.50) | 146.06 | −1.17(−2.27-−0.06) |
| Kenya | 0.22(0.16-0.39) | 2.65(54.36-93.49) | 0.72(0.49-1.06) | 3.34(2.28-4.81) | 223.93 | 0.27(−0.10-0.65) |
| Kiribati | 0.01(0-0.01) | 14.29(33.79-55.32) | 0.01(0.01-0.01) | 11.95(9.29-15.08) | 56.16 | −0.67(−0.71-−0.62) |
| Kuwait | 0.02(0.02-0.02) | 2.76(1.72-2.43) | 0.05(0.04-0.07) | 2.36(1.85-2.96) | 215.19 | 0.23(−0.1-0.56) |
| Kyrgyzstan | 0.04(0.04-0.04) | 1.32(33.59-57.01) | 0.12(0.10-0.14) | 2.70(2.31-3.09) | 189.04 | 3.34(2.95-3.73) |
| Lao People's Democratic Republic | 0.22(0.15-0.3) | 10.47(31.28-51.69) | 0.31(0.23-0.40) | 7.11(5.4-9.01) | 38.57 | −1.61(−1.73-−1.49) |
| Latvia | 0.06(0.06-0.06) | 1.70(7.43-10.5) | 0.11(0.09-0.13) | 2.70(2.25-3.29) | 76.39 | 0.76(0-1.54) |
| Lebanon | 0.08(0.07-0.10) | 3.56(2.33-3.17) | 0.15(0.11-0.22) | 2.97(2.19-4.14) | 94.74 | −0.68(−0.77-−0.59) |
| Lesotho | 0.05(0.03-0.11) | 5.42(54.83-93.16) | 0.20(0.08-0.31) | 15.17(6.45-23.71) | 270.95 | 3.99(3.29-4.71) |
| Liberia | 0.08(0.06-0.10) | 7.22(44.49-75.01) | 0.11(0.08-0.16) | 5.29(3.89-7.78) | 33.98 | −1.27(−1.42-−1.12) |
| Libya | 0.10(0.08-0.13) | 5.37(22.84-38.66) | 0.26(0.19-0.35) | 5.05(3.79-6.86) | 153.89 | −0.19(−0.31-−0.08) |
| Lithuania | 0.07(0.07-0.08) | 1.66(7.66-10.61) | 0.17(0.13-0.21) | 3.03(2.38-3.70) | 127.60 | 1.42(1.06-1.77) |
| Luxembourg | 0.02(0.02-0.02) | 2.96(3.41-4.57) | 0.04(0.03-0.05) | 3.62(2.94-4.50) | 123.71 | 0.76(0.53-0.99) |
| Madagascar | 0.17(0.12-0.29) | 3.07(60.15-102.57) | 0.29(0.20-0.43) | 2.62(1.80-4.02) | 71.37 | −0.74(−0.86-−0.62) |
| Malawi | 0.17(0.12-0.23) | 3.42(66.66-114.41) | 0.24(0.19-0.30) | 3.04(2.43-3.74) | 46.70 | −1.31(−1.7-−0.92) |
| Malaysia | 0.53(0.46-0.62) | 5.78(22.39-37.18) | 1.63(1.24-2.10) | 6.22(4.78-7.94) | 206.21 | 0.40(0.11-0.70) |
| Maldives | 0.01(0-0.01) | 7.33(21.81-36.56) | 0.02(0.01-0.02) | 5.63(4.46-6.89) | 171.65 | −0.86(−0.99-−0.73) |
| Mali | 0.71(0.59-0.85) | 15.70(47.92-80.66) | 1.37(1.01-1.82) | 15.03(11.25-19.52) | 93.03 | −0.32(−0.42-−0.22) |
| Malta | 0.01(0.01-0.01) | 1.64(2.07-2.64) | 0.02(0.02-0.02) | 2.20(1.93-2.49) | 192.60 | 1.08(0.84-1.32) |
| Marshall Islands | 0 | 12.14(31.97-52.81) | 0(0-0.01) | 10.57(7.64-14.31) | 85.61 | −0.47(−0.54-−0.40) |
| Mauritania | 0.07(0.06-0.09) | 7.31(39.92-67.76) | 0.09(0.07-0.12) | 4.43(3.30-5.68) | 21.01 | −1.78(−1.91-−1.64) |
| Mauritius | 0.01(0.01-0.01) | 1.90(22.08-37.21) | 0.03(0.03-0.04) | 2.00(1.55-2.57) | 146.25 | 1.45(1.09-1.81) |
| Mexico | 1.01(0.97-1.04) | 2.43(16.16-25.77) | 4.18(3.61-4.79) | 3.69(3.18-4.22) | 314.24 | 1.49(1.33-1.64) |
| Micronesia  (Federated States of) | 0.01(0-0.01) | 11.58(32.13-52.53) | 0.01(0-0.01) | 10.70(7.24-14.74) | 40.96 | −0.35(−0.48-−0.21) |
| Monaco | 0 | 3.24(2.48-3.60) | 0.01(0.01-0.01) | 7.63(6.05-9.39) | 219.82 | 3.65(2.83-4.47) |
| Mongolia | 0.69(0.56-0.84) | 66.77(35.91-59.98) | 2.37(1.82-3.04) | 115.23(91.48-142.48) | 241.41 | 2.65(2.18-3.13) |
| Montenegro | 0.04(0.03-0.04) | 6.26(4.11-5.47) | 0.06(0.05-0.07) | 6.09(4.93-7.48) | 54.52 | −0.15(−0.37-0.07) |
| Morocco | 0.26(0.19-0.32) | 2.07(25.11-41.91) | 0.65(0.50-0.80) | 2.31(1.77-2.79) | 152.23 | 0.20(−0.06-0.45) |
| Mozambique | 0.14(0.10-0.22) | 2.30(65.59-112.83) | 0.46(0.32-0.61) | 3.99(2.79-5.24) | 229.17 | 1.86(1.66-2.06) |
| Myanmar | 0.68(0.49-1.00) | 3.03(30.06-49.59) | 1.96(1.63-2.32) | 4.44(3.76-5.26) | 188.03 | 1.28(1.09-1.46) |
| Namibia | 0.01(0.01-0.03) | 1.98(49.70-87.25) | 0.05(0.04-0.06) | 3.46(2.66-4.39) | 245.35 | 2.02(1.62-2.43) |
| Nauru | 0 | 10.89(16.47-20.29) | 0 | 9.39(6.7-12.73) | −2.44 | −0.68(−0.97-−0.39) |
| Nepal | 0.19(0.14-0.24) | 1.97(27.40-45.69) | 0.49(0.35-0.70) | 2.36(1.71-3.39) | 160.58 | 0.70(0.58-0.82) |
| Netherlands | 0.28(0.26-0.29) | 1.39(2.25-3.00) | 0.94(0.86-1.02) | 2.75(2.53-2.97) | 241.33 | 2.67(2.53-2.81) |
| New Zealand | 0.08(0.08-0.09) | 2.12(4.10-5.34) | 0.28(0.26-0.30) | 3.72(3.44-4.00) | 243.77 | 2.16(1.99-2.33) |
| Nicaragua | 0.05(0.04-0.06) | 3.24(39.81-66.91) | 0.17(0.14-0.21) | 4.10(3.37-4.96) | 237.33 | 0.97(0.63-1.32) |
| Niger | 0.02(0.02-0.03) | 0.71(42.12-71.15) | 0.05(0.04-0.07) | 0.65(0.49-0.84) | 154.04 | −0.41(−0.49-−0.33) |
| Nigeria | 1.65(1.26-2.06) | 3.57(42.28-72.00) | 2.92(2.28-3.70) | 3.57(2.87-4.44) | 77.43 | 0.08(0.02-0.14) |
| Niue | 0 | 8.32(10.5-13.42) | 0 | 7.34(5.67-9.36) | −12.50 | −0.54(−0.58-−0.50) |
| North Macedonia | 0.17(0.15-0.18) | 9.13(3.76-5.02) | 0.28(0.22-0.36) | 8.82(7.01-11.10) | 66.34 | −0.23(−0.35-−0.11) |
| Northern Mariana Islands | 0 | 6.93(27.78-46.98) | 0(0-0.01) | 7.84(6.50-9.45) | 207.66 | 0.73(0.55-0.90) |
| Norway | 0.10(0.10-0.11) | 1.54(1.79-2.52) | 0.23(0.21-0.26) | 2.45(2.20-2.76) | 124.90 | 2.07(1.90-2.25) |
| Oman | 0.03(0.02-0.04) | 4.36(2.21-3.01) | 0.08(0.07-0.10) | 4.75(3.98-5.69) | 178.02 | 1.06(0.67-1.45) |
| Pakistan | 2.06(1.43-2.75) | 3.47(39.03-65.25) | 3.91(3.16-4.83) | 3.46(2.75-4.30) | 89.84 | −0.04(−0.19-0.10) |
| Palau | 0 | 9.78(8.41-10.62) | 0 | 9.79(7.59-12.53) | 122.45 | 0(−0.06-0.07) |
| Palestine | 0.07(0.06-0.10) | 9(22.49-37.38) | 0.14(0.12-0.17) | 6.61(5.56-7.88) | 91.27 | −1.08(−1.16-1.00) |
| Panama | 0.05(0.05-0.06) | 3.45(34.22-57.44) | 0.13(0.10-0.16) | 3.06(2.32-3.93) | 143.54 | −0.09(−0.49-0.32) |
| Papua New Guinea | 0.02(0.02-0.03) | 1.52(32.29-52.61) | 0.07(0.05-0.09) | 1.67(1.31-2.12) | 167.38 | 0.40(0.38-0.43) |
| Paraguay | 0.05(0.05-0.06) | 2.47(36.82-62.99) | 0.12(0.09-0.15) | 2.19(1.64-2.82) | 115.13 | −0.29(−0.84-0.25) |
| Peru | 0.74(0.64-0.85) | 6.21(35.24-59.22) | 0.87(0.65-1.15) | 2.74(2.04-3.61) | 17.41 | −3.57(−4.15-−2.99) |
| Philippines | 3.15(2.36-3.84) | 10.04(19.49-32.54) | 5.28(4.27-6.45) | 6.58(5.35-7.98) | 67.6 | −1.84(−2.12-−1.56) |
| Poland | 3.24(3.10-3.34) | 7.57(4.92-6.68) | 1.45(1.22-1.72) | 2.06(1.73-2.44) | −55.12 | −4.30(−5.60-−2.99) |
| Portugal | 0.26(0.25-0.28) | 1.92(2.71-3.45) | 1.05(0.96-1.14) | 4.59(4.19-4.97) | 297.52 | 2.76(2.41-3.10) |
| Puerto Rico | 0.25(0.24-0.27) | 6.97(2.15-3.04) | 0.19(0.15-0.25) | 2.74(2.04-3.55) | −23.62 | −2.18(−2.93-−1.42) |
| Qatar | 0.01(0.01-0.02) | 17.37(2.47-3.50) | 0.09(0.06-0.12) | 15.88(11.76-20.79) | 526.52 | −0.13(−0.34-0.08) |
| Republic of Korea | 3.41(2.83-4.17) | 11.04(1.30-1.79) | 14.48(12.94-16.04) | 16.2(14.47-17.94) | 324.2 | 1.97(0.73-3.23) |
| Republic of Moldova | 0.07(0.07-0.08) | 1.70(5.24-7.05) | 0.14(0.12-0.16) | 2.40(2.03-2.81) | 85.47 | −1.07(−2.59-0.48) |
| Romania | 0.50(0.45-0.57) | 1.85(5.03-6.74) | 1.11(0.91-1.34) | 3.08(2.50-3.75) | 120.66 | 2.50(2.07-2.93) |
| Russian Federation | 3.10(2.96-3.27) | 1.76(7.46-9.71) | 6.83(5.81-8.14) | 2.97(2.53-3.55) | 120.13 | 2.25(1.97-2.53) |
| Rwanda | 0.16(0.12-0.22) | 5.36(59.65-102.68) | 0.29(0.22-0.37) | 4.72(3.78-5.98) | 76 | −1.12(−1.39-−0.85) |
| Saint Kitts and Nevis | 0.01(0-0.01) | 13.47(2.66-3.51) | 0 | 3.88(3.28-4.57) | −51.44 | −4.55(−5.56-−3.54) |
| Saint Lucia | 0.01(0.01-0.01) | 6.45(37.94-64.7) | 0(0-0.01) | 2.29(1.91-2.73) | −14.09 | −3.84(−4.86-−2.82) |
| Saint Vincent and the Grenadines | 0.01(0.01-0.01) | 7.61(38.2-64.67) | 0 | 3.13(2.72-3.61) | −24.14 | −2.99(−3.86-−2.11) |
| Samoa | 0.01(0-0.01) | 6.19(30.66-51.43) | 0.01(0.01-0.01) | 5.06(3.91-6.33) | 36.53 | −0.72(−0.80-−0.63) |
| San Marino | 0 | 2.07(4.04-5.34) | 0 | 2.75(1.80-3.98) | 156 | 1.47(1.26-1.69) |
| Sao Tome and Principe | 0 | 2.6(40.82-69.56) | 0 | 2.80(1.89-3.69) | 80.66 | 0.19(0.08-0.30) |
| Saudi Arabia | 0.33(0.23-0.43) | 5.91(2.44-3.22) | 0.73(0.55-0.96) | 4.90(3.86-6.25) | 121.73 | −0.96(−1.21-−0.71) |
| Senegal | 0.08(0.06-0.09) | 2.39(41.35-71.66) | 0.17(0.12-0.21) | 2.21(1.65-2.78) | 109.23 | −0.19(−0.32-−0.06) |
| Serbia | 0.7(0.58-0.81) | 6.27(3.56-4.78) | 0.88(0.70-1.11) | 5.49(4.36-6.87) | 26.41 | −0.68(−0.96-−0.40) |
| Seychelles | 0(0-0.01) | 8.01(21.85-37.05) | 0.01(0.01-0.01) | 5.70(4.81-6.72) | 38.25 | −1.08(−1.34-−0.82) |
| Sierra Leone | 0.12(0.09-0.16) | 6.24(44.03-74.83) | 0.18(0.13-0.24) | 4.94(3.79-6.39) | 47.99 | −0.88(−0.97-−0.79) |
| Singapore | 0.18(0.17-0.19) | 8.27(1.33-1.96) | 0.66(0.59-0.73) | 8.68(7.67-9.66) | 265.26 | 0.17(−0.13-0.47) |
| Slovakia | 0.28(0.26-0.30) | 4.66(5.95-8.48) | 0.31(0.24-0.40) | 3.39(2.64-4.28) | 11.99 | −1.65(−1.97-−1.34) |
| Slovenia | 0.07(0.06-0.09) | 3.00(4.99-6.57) | 0.22(0.17-0.28) | 5.14(3.93-6.61) | 201.15 | 2.11(1.78-2.45) |
| Solomon Islands | 0.01(0.01-0.01) | 6.81(33.67-54.87) | 0.02(0.01-0.02) | 5.52(4.46-6.68) | 76.94 | −0.71(−0.79-−0.63) |
| Somalia | 0.10(0.06-0.17) | 3.76(66.29-113.84) | 0.25(0.16-0.43) | 3.61(2.35-6.40) | 148.29 | −0.06(−0.14-0.01) |
| South Africa | 1.31(0.87-2.26) | 6.04(54.94-93.23) | 2.64(2.33-3.00) | 5.87(5.18-6.68) | 101.00 | −0.57(−1.19-0.06) |
| South Sudan | 0.08(0.05-0.15) | 3.26(51.88-88.51) | 0.11(0.07-0.20) | 2.97(1.79-5.14) | 40.29 | −0.28(−0.33-−0.23) |
| Spain | 2.19(2.07-2.29) | 3.98(3.95-4.97) | 4.97(4.47-5.44) | 5.18(4.67-5.67) | 127.20 | 0.70(0.41-0.98) |
| Sri Lanka | 0.23(0.20-0.26) | 2.22(4.09-5.60) | 0.71(0.51-0.94) | 2.84(2.11-3.77) | 208.57 | 1.84(1.49-2.19) |
| Sudan | 0.34(0.20-0.52) | 3.80(27.5-45.59) | 0.68(0.41-1.07) | 3.92(2.41-6.04) | 98.68 | 0.09(−0.04-0.22) |
| Suriname | 0.02(0.02-0.02) | 6.83(36.57-62.21) | 0.02(0.01-0.02) | 2.74(2.21-3.38) | −8.33 | −3.12(−3.88-−2.35) |
| Sweden | 0.38(0.36-0.40) | 2.52(1.86-2.56) | 0.62(0.57-0.67) | 3.02(2.80-3.24) | 64.11 | 1.19(0.61-1.78) |
| Switzerland | 0.24(0.23-0.26) | 2.36(2.52-3.50) | 0.77(0.68-0.85) | 4.43(3.97-4.91) | 213.32 | 1.72(1.20-2.23) |
| Syrian Arab Republic | 0.26(0.20-0.33) | 5.21(23.72-39.53) | 0.50(0.37-0.67) | 4.41(3.35-5.77) | 90.18 | −0.75(−0.92-−0.57) |
| Taiwan  (Province of China) | 2.02(1.94-2.11) | 11.98(8.04-10.12) | 2.85(2.23-3.66) | 7.27(5.69-9.32) | 40.85 | −2.69(−3.5-−1.86) |
| Tajikistan | 0.03(0.03-0.04) | 1.07(35.00-58.71) | 0.17(0.14-0.22) | 4.02(3.23-5.06) | 432.00 | 5.31(5.04-5.58) |
| Thailand | 7.88(6.84-9.01) | 21.6(22.02-36.63) | 24.53(18.17-32.51) | 24.01(17.88-31.65) | 211.14 | 0.48(0.36-0.61) |
| Timor−Leste | 0.02(0.02-0.03) | 7.84(26.08-43.83) | 0.05(0.04-0.07) | 6.60(4.53-9.09) | 127.94 | −0.79(−0.96-−0.62) |
| Togo | 0.09(0.07-0.11) | 6.76(43.49-73.41) | 0.19(0.15-0.25) | 5.24(4.09-6.74) | 119.67 | −1.31(−1.49-−1.13) |
| Tokelau | 0 | 8.64(12.22-15.27) | 0 | 7.48(5.46-10.06) | −14.90 | −0.58(−0.61-−0.54) |
| Tonga | 0.01(0.01-0.02) | 23.93(40.83-67.04) | 0.02(0.01-0.03) | 24.74(18.09-32.04) | 45.24 | 0.06(−0.08-0.20) |
| Trinidad and Tobago | 0.06(0.06-0.06) | 7.10(36.62-61.95) | 0.05(0.04-0.06) | 2.66(2.03-3.46) | −17.75 | −3.59(−4.59-−2.57) |
| Tunisia | 0.10(0.07-0.12) | 1.98(2.23-2.92) | 0.22(0.16-0.31) | 1.80(1.27-2.53) | 128.08 | −0.23(−0.30-−0.16) |
| Turkey | 1.38(1.10-1.67) | 3.92(1.91-2.53) | 2.54(2.01-3.12) | 2.96(2.36-3.63) | 84.23 | −1.00(−1.18-−0.82) |
| Turkmenistan | 0.02(0.02-0.03) | 1.21(32.83-55.34) | 0.23(0.18-0.29) | 5.59(4.41-7.07) | 837.39 | 6.16(5.16-7.17) |
| Tuvalu | 0 | 10.76(15.49-19.17) | 0 | 8.72(6.51-11.52) | 19.18 | −0.84(−0.97-−0.70) |
| Uganda | 0.33(0.27-0.40) | 4.75(68.41-128.32) | 0.97(0.76-1.22) | 6.39(5.12-7.88) | 195.69 | 1.17(0.98-1.37) |
| Ukraine | 0.70(0.67-0.74) | 1.02(5.54-7.31) | 1.96(1.67-2.29) | 2.66(2.24-3.11) | 178.90 | 4.11(3.55-4.68) |
| United Arab Emirates | 0.02(0.01-0.04) | 4.57(20.94-34.70) | 0.20(0.08-0.46) | 4.9(2.06-11.30) | 931.31 | 0.17(−0.08-0.42) |
| United Kingdom | 1.68(1.61-1.72) | 1.87(2.14-2.84) | 5.16(4.75-5.46) | 4.03(3.75-4.26) | 206.71 | 3.37(3.17-3.57) |
| United Republic of Tanzania | 0.27(0.22-0.33) | 2.21(60.37-103.32) | 0.64(0.50-0.81) | 2.46(1.98-3.02) | 136.15 | 0.31(0.17-0.45) |
| United States of America | 6.45(6.18-6.62) | 2.04(8.59-10.38) | 23.81(21.18-26.10) | 4.33(3.86-4.75) | 268.88 | 2.64(2.49-2.80) |
| United States Virgin Islands | 0 | 4.29(2.64-3.67) | 0(0-0.01) | 2.60(2.17-3.07) | 35.16 | −1.74(−2.32-−1.16) |
| Uruguay | 0.06(0.05-0.06) | 1.43(3.98-5.20) | 0.13(0.12-0.14) | 2.36(2.12-2.61) | 129.05 | 2.14(1.97-2.30) |
| Uzbekistan | 0.11(0.10-0.12) | 0.95(34.73-58.31) | 1.32(1.08-1.59) | 6.68(5.58-7.83) | 1075.97 | 9.34(8.30-10.4) |
| Vanuatu | 0.01(0-0.01) | 9.62(34.96-57.29) | 0.02(0.01-0.02) | 9.36(6.41-13.2) | 156.99 | −0.07(−0.18-0.05) |
| Venezuela | 0.64(0.61-0.67) | 6.75(2.19-2.98) | 0.69(0.53-0.89) | 2.44(1.88-3.15) | 7.58 | −2.84(−4.05-−1.62) |
| Viet Nam | 1.58(1.18-1.98) | 3.97(6.54-8.70) | 2.39(1.84-2.98) | 2.75(2.15-3.40) | 51.35 | −1.49(−1.7-−1.29) |
| Yemen | 0.16(0.10-0.23) | 3.42(32.89-53.99) | 0.42(0.31-0.58) | 3.42(2.49-4.57) | 165.77 | 0.04(−0.04-0.13) |
| Zambia | 0.08(0.06-0.11) | 2.60(60.76-103.95) | 0.20(0.16-0.25) | 2.99(2.37-3.67) | 151.99 | −0.17(−0.54-0.2) |
| Zimbabwe | 0.51(0.40-0.79) | 12.16(53.44-91.30) | 1.02(0.75-1.38) | 14.03(10.58-18.67) | 99.27 | −0.48(−1.05-0.11) |

EAPC: estimated annual percentage change; ASDR, age-standardized death rate; CI, confidence interval; UI: uncertainty interval.

**Supplementary table 2.** The percentage change in number and the EAPCs of death attribute to liver cancer caused by specific etiologies in global, sexes, SDI areas and geographic regions from 1990 to 2019.

|  | **LCHB** | | | **LCHC** | | |  |
| --- | --- | --- | --- | --- | --- | --- | --- |
| **Characteristics** | Number in 2019  ×10^3^ (95% UI) | Changes in  number (%) | EAPC  (95%CI) | Number in 2019  ×10^3^ (95% UI) | Changes in  number (%) | EAPC  (95%CI) | |
| **Overall** | 191.74(161.86-223.73) | 0.76 | −3.46(−4.01-−2.89) | 141.81(121.79-161.83) | 67.50 | −1.35(−1.59-−1.11) | |
| **Sex** |  |  |  |  |  |  | |
| Male | 157.06(131.21-186.54) | 2.17 | −3.44(−4.03-−2.85) | 74.37(62.93-86.18) | 79.80 | −1.12(−1.39-−0.85) | |
| Female | 34.67(27.99-42.28) | −5.18 | −3.41(−3.84-−2.99) | 67.44(56.53-76.81) | 55.74 | −1.57(−1.79-−1.35) | |
| **SDI** |  |  |  |  |  |  | |
| Low | 7.46(5.92-9.16) | 89.19 | −0.73(−0.83-−0.64) | 5.32(4.14-6.47) | 99.75 | −0.50(−0.56-−0.45) | |
| Low-middle | 23.19(19.84-26.87) | 30.26 | −2.59(−3.02-−2.16) | 14.20(11.42-16.83) | 107.83 | −0.84(−1.00-−0.67) | |
| Middle | 98.32(81.73-117.89) | −0.19 | −3.95(−4.6-−3.31) | 45.17(37.16-54.31) | 48.28 | −2.51(−2.93-−2.09) | |
| High-middle | 41.65(34.36-50.27) | −29.83 | −4.85(−5.54-−4.15) | 26.55(22.66-30.48) | 15.14 | −2.70(−3.06-−2.34) | |
| High | 21.06(17.52-24.91) | 97.81 | 0.13(−0.39-0.65) | 50.52(43.62-56.77) | 133.62 | 0.10(−0.37-0.58) | |
| **Regions** |  |  |  |  |  |  | |
| East Asia | 120.12(97.66-146.17) | −22.66 | −5.11(−5.88-−4.33) | 34.88(28.7-41.39) | −8.60 | −4.92(−5.59-−4.24) | |
| South Asia | 12.36(10.14-14.96) | 113.57 | −0.28(−0.42-−0.14) | 9.90(8.09-11.94) | 164.69 | −0.26(−0.36-−0.16) | |
| Southeast Asia | 16.44(12.18-21.72) | 111.29 | −0.29(−0.37-−0.21) | 9.71(7.33-12.7) | 147.09 | 0.16(0.09-0.23) | |
| Central Asia | 1.65(1.18-2.21) | 282.81 | 2.25(1.70-2.80) | 1.99(1.42-2.60) | 303.21 | 3.28(2.74-3.81) | |
| High-income Asia Pacific | 11.62(9.71-13.64) | 120.41 | 0.62(−0.15-1.40) | 27.96(23.73-30.94) | 105.75 | −1.54(−2.13-−0.95) | |
| Oceania | 0.12(0.09-0.16) | 96.72 | −0.43(−0.51-−0.35) | 0.05(0.03-0.07) | 112.32 | −0.20(−0.27-−0.12) | |
| Australasia | 0.25(0.18-0.35) | 256.37 | 2.29(2.08-2.50) | 0.68(0.51-0.86) | 392.76 | 3.17(2.99-3.34) | |
| Eastern Europe | 2.12(1.68-2.65) | 97.74 | 2.17(1.87-2.47) | 2.72(2.28-3.21) | 134.20 | 2.49(2.27-2.70) | |
| Western Europe | 4.30(3.13-5.82) | 76.39 | 0.46(0.33-0.59) | 17.57(14.48-20.79) | 104.73 | 0.65(0.50-0.80) | |
| Central Europe | 1.37(0.97-1.91) | −26.45 | −2.00(−2.30-−1.70) | 1.93(1.41-2.57) | −12.56 | −1.77(−2.18-−1.36) | |
| High-income North America | 2.91(2.35-3.59) | 225.05 | 2.34(2.15-2.52) | 9.75(8.36-11.23) | 282.39 | 2.75(2.55-2.95) | |
| Andean Latin America | 0.76(0.55-1.00) | 50.34 | −2.38(−2.78-−1.98) | 0.15(0.09-0.23) | 101.96 | −1.64(−2.04-−1.24) | |
| Central Latin America | 1.16(0.86-1.60) | 115.08 | −0.70(−1.03-−0.36) | 3.39(2.74-4.11) | 184.54 | −0.05(−0.34-0.23) | |
| Caribbean | 0.44(0.31-0.61) | −2.95 | −2.35(−3.17-−1.52) | 0.39(0.26-0.54) | −0.68 | −2.46(−3.27-−1.65) | |
| Tropical Latin America | 1.02(0.86-1.20) | 149.28 | 0.38(0.26-0.49) | 2.42(2.11-2.70) | 243.54 | 1.14(0.93-1.34) | |
| Southern Latin America | 0.32(0.22-0.47) | 137.53 | 1.64(1.45-1.83) | 0.74(0.54-0.93) | 186.51 | 2.11(1.89-2.33) | |
| Eastern Sub-Saharan Africa | 1.62(1.19-2.16) | 125.66 | −0.20(−0.37-−0.04) | 1.28(0.95-1.64) | 133.37 | 0.21(0.11-0.30) | |
| Southern Sub-Saharan Africa | 1.50(1.25-1.79) | 101.30 | −0.72(−1.35-−0.08) | 1.10(0.91-1.31) | 114.50 | −0.26(−0.76-0.23) | |
| Western Sub-Saharan Africa | 4.56(3.57-5.70) | 84.51 | −0.69(−0.80-−0.58) | 1.91(1.37-2.46) | 84.54 | −0.45(−0.50-−0.40) | |
| North Africa and Middle East | 6.74(5.02-8.81) | 113.62 | −0.58(−0.70-−0.47) | 12.74(9.25-17.06) | 149.33 | 0.47(0.26-0.67) | |
| Central Sub-Saharan Africa | 0.35(0.24-0.52) | 97.57 | −0.89(−1.00-−0.78) | 0.57(0.40-0.75) | 107.76 | −0.60(−0.68-−0.53) | |

LCHB, liver cancer due to hepatitis B; LCHC, liver cancer due to hepatitis C; EAPC: estimated annual percentage change; CI, confidence interval; SDI: socio-demographic index.

**Supplementary table 3.** The percentage change in number and the EAPCs of death due to liver cancer caused by specific etiologies at national level from 1990 to 2019.

|  | | **LCHB** | | | | | | | **LCHC** | | | | | | |
| --- | --- | --- | --- | --- | --- | --- | --- | --- | --- | --- | --- | --- | --- | --- | --- |
| **Characteristics** | | | ASDR/100,00  in 2019 | | Changes in number (%) | | EAPC  (95%CI) | | ASDR/100,00  in 2019 | | Changes in number (%) | | EAPC  (95%CI) | |  |
| Afghanistan | 3.60(2.51-4.96) | | | 72.01 | | −0.74(−0.85-−0.64) | | 4.13(2.91-5.72) | | 42.06 | | −0.61(−0.73-−0.50) | |  | |
| Albania | 1.64(1.04-2.44) | | | −2.80 | | −3.48(−4.08-−2.86) | | 1.85(1.19-2.73) | | 20.76 | | −3.48(−4.08-−2.87) | |  | |
| Algeria | 0.68(0.46-0.96) | | | 204.56 | | 0.40(0.20-0.61) | | 0.89(0.64-1.21) | | 250.36 | | 0.64(0.48-0.80) | |  | |
| American Samoa | 3.58(2.73-4.65) | | | 149.05 | | 1.22(1.03-1.40) | | 1.53(1.05-2.08) | | 159.56 | | 0.78(0.53-1.02) | |  | |
| Andorra | 1.12(0.70-1.75) | | | 128.22 | | −0.18(−0.28-−0.08) | | 3.51(2.33-4.97) | | 205.53 | | 0.29(0.14-0.45) | |  | |
| Angola | 0.55(0.37-0.79) | | | 147.78 | | −0.73(−0.84-−0.63) | | 1.26(0.93-1.65) | | 153.39 | | −0.55(−0.64-−0.46) | |  | |
| Antigua and Barbuda | 0.69(0.48-0.97) | | | −43.91 | | −4.64(−5.61-−3.67) | | 0.71(0.49-0.98) | | −39.77 | | −3.76(−4.71-−2.79) | |  | |
| Argentina | 0.35(0.24-0.50) | | | 129.37 | | 1.80(1.61-2.00) | | 0.77(0.57-0.98) | | 159.02 | | 2.09(1.86-2.31) | |  | |
| Armenia | 1.40(0.94-2.03) | | | 748.77 | | 9.21(7.65-10.79) | | 2.78(2-3.62) | | 971.94 | | 9.54(7.99-11.12) | |  | |
| Australia | 0.55(0.38-0.79) | | | 270.85 | | 2.36(2.14-2.59) | | 1.37(1.00-1.78) | | 413.95 | | 3.3(3.11-3.48) | |  | |
| Austria | 0.31(0.20-0.46) | | | 74.50 | | 0.61(0.31-0.91) | | 1.37(0.99-1.85) | | 111.55 | | 1.45(1.15-1.75) | |  | |
| Azerbaijan | 0.94(0.62-1.39) | | | 674.63 | | 6.07(3.91-8.28) | | 1.72(1.13-2.58) | | 618.27 | | 6.14(4.08-8.23) | |  | |
| Bahamas | 0.87(0.62-1.21) | | | −11.09 | | −3.73(−4.54-−2.92) | | 0.72(0.48-1.00) | | 3.65 | | −3.32(−4.14-−2.50) | |  | |
| Bahrain | 1.4(0.95-2.02) | | | 320.43 | | −2.08(−2.55-−1.62) | | 2.25(1.6-2.96) | | 328.86 | | −1.03(−1.46-−0.60) | |  | |
| Bangladesh | 0.56(0.39-0.82) | | | 50.24 | | −1.97(−2.17-−1.76) | | 1.24(0.89-1.61) | | 167.07 | | −0.36(−0.51-−0.22) | |  | |
| Barbados | 0.60(0.43-0.86) | | | 35.48 | | −1.16(−1.39-−0.94) | | 0.64(0.44-0.91) | | 48.45 | | −0.35(−0.52-−0.19) | |  | |
| Belarus | 0.47(0.29-0.74) | | | 96.96 | | 2.66(1.89-3.43) | | 0.61(0.4-0.87) | | 110.95 | | 2.40(1.90-2.89) | |  | |
| Belgium | 0.47(0.32-0.67) | | | 73.74 | | 0.59(0.31-0.86) | | 1.42(1.07-1.81) | | 85.31 | | 0.50(0.07-0.93) | |  | |
| Belize | 0.83(0.60-1.14) | | | 49.53 | | −2.79(−3.33-−2.23) | | 0.74(0.5-1.02) | | 22.03 | | −2.94(−3.57-−2.31) | |  | |
| Benin | 2.22(1.53-3.08) | | | 66.44 | | −1.84(−2.00-−1.67) | | 1.08(0.71-1.52) | | 73.15 | | −1.11(−1.24-−0.99) | |  | |
| Bermuda | 0.49(0.33-0.69) | | | −46.22 | | −4.29(−5.37-−3.20) | | 0.45(0.29-0.64) | | −38.68 | | −4.66(−5.67-−3.64) | |  | |
| Bhutan | 0.75(0.44-1.17) | | | 139.79 | | 0.58(0.45-0.71) | | 1.12(0.70-1.73) | | 271.65 | | 1.81(1.70-1.92) | |  | |
| Bolivia | 2.08(1.43-2.87) | | | 125.83 | | −0.75(−0.83-−0.66) | | 0.47(0.27-0.75) | | 174.23 | | −0.15(−0.23-−0.08) | |  | |
| Bosnia and Herzegovina | 1.69(1.12-2.54) | | | 42.95 | | 0.43(0.12-0.75) | | 2.50(1.74-3.50) | | 141.16 | | 2.06(1.63-2.50) | |  | |
| Botswana | 0.51(0.33-0.75) | | | 284.77 | | 0.30(−0.61-1.23) | | 0.46(0.30-0.64) | | 218.09 | | 0.38(−0.18-0.95) | |  | |
| Brazil | 0.42(0.35-0.49) | | | 149.98 | | 0.39(0.27-0.51) | | 1.04(0.91-1.15) | | 247.10 | | 1.17(0.97-1.36) | |  | |
| Brunei Darussalam | 4.91(3.67-6.40) | | | 263.72 | | 1.28(0.79-1.79) | | 4.39(3.24-5.71) | | 276.03 | | 0.97(0.45-1.49) | |  | |
| Bulgaria | 1.00(0.67-1.47) | | | −36.28 | | −1.18(−1.64-−0.72) | | 1.05(0.69-1.51) | | −18.65 | | −0.75(−1.30-−0.19) | |  | |
| Burkina Faso | 0.87(0.60-1.19) | | | 50.38 | | −1.57(−1.74-−1.40) | | 0.68(0.45-0.94) | | 43.92 | | −1.47(−1.68-−1.26) | |  | |
| Burundi | 0.79(0.46-1.33) | | | 60.75 | | −1.41(−1.60-−1.22) | | 0.81(0.50-1.22) | | 57.25 | | −0.90(−1.07-−0.73) | |  | |
| Cabo Verde | 5.36(4.04-6.98) | | | 1846.13 | | 6.16(3.81-8.57) | | 2.56(1.68-3.63) | | 1488.53 | | 6.22(4.12-8.35) | |  | |
| Cambodia | 2.95(2.05-4.14) | | | 72.77 | | −1.24(−1.37-−1.11) | | 3.92(2.84-5.08) | | 120.40 | | −0.82(−0.93-−0.71) | |  | |
| Cameroon | 0.31(0.21-0.44) | | | 167.32 | | −0.47(−0.77-−0.17) | | 0.12(0.08-0.18) | | 145.92 | | −0.36(−0.52-−0.21) | |  | |
| Canada | 0.27(0.18-0.40) | | | 251.19 | | 2.32(2.12-2.53) | | 0.73(0.50-1.02) | | 357.57 | | 2.92(2.68-3.16) | |  | |
| Central African  Republic | 0.82(0.47-1.35) | | | 52.87 | | −1.20(−1.46-−0.94) | | 1.62(1.01-2.37) | | 65.53 | | −0.73(−0.95-−0.51) | |  | |
| Chad | 2.76(1.93-3.79) | | | 71.37 | | −0.77(−0.89-−0.66) | | 1.21(0.77-1.72) | | 54.24 | | −0.71(−0.78-−0.63) | |  | |
| Chile | 0.48(0.33-0.69) | | | 162.51 | | 1.06(0.83-1.29) | | 1.10(0.80-1.42) | | 262.59 | | 1.84(1.58-2.10) | |  | |
| China | 5.76(4.68-7.02) | | | −23.34 | | −5.17(−5.96-−4.37) | | 1.75(1.45-2.07) | | −10.99 | | −5.07(−5.79-−4.35) | |  | |
| Colombia | 0.44(0.28-0.66) | | | 108.43 | | −0.84(−1.27-−0.4) | | 1.02(0.69-1.41) | | 185.06 | | −0.42(−0.85-0.01) | |  | |
| Comoros | 0.84(0.49-1.44) | | | 81.09 | | −0.92(−1.09-−0.75) | | 0.86(0.54-1.31) | | 112.26 | | −0.26(−0.35-−0.17) | |  | |
| Congo | 0.66(0.42-1.00) | | | 59.74 | | −1.83(−2.01-−1.66) | | 1.55(1.08-2.19) | | 71.11 | | −1.37(−1.51-−1.24) | |  | |
| Cook Islands | 5.18(3.82-6.86) | | | 35.15 | | −0.96(−1.16-−0.77) | | 2.39(1.61-3.34) | | 59.65 | | −0.98(−1.06-−0.90) | |  | |
| Costa Rica | 0.74(0.47-1.14) | | | 131.05 | | −0.95(−1.43-−0.48) | | 1.99(1.35-2.72) | | 173.88 | | −0.56(−1.07-−0.05) | |  | |
| Croatia | 0.74(0.49-1.11) | | | 29.09 | | 0.16(−0.34-0.67) | | 0.67(0.43-0.96) | | 45.93 | | 0.17(−0.40-0.73) | |  | |
| Cuba | 0.58(0.39-0.83) | | | −42.71 | | −4.28(−5.61-−2.93) | | 0.56(0.37-0.80) | | −36.27 | | −4.13(−5.32-−2.92) | |  | |
| Cyprus | 0.39(0.26-0.57) | | | 96.68 | | −0.54(−0.77-−0.30) | | 1.40(1.05-1.79) | | 161.01 | | 0.31(0-0.63) | |  | |
| Czechia | 0.54(0.36-0.80) | | | −19.31 | | −2.21(−2.54-−1.89) | | 0.69(0.45-0.98) | | 3.80 | | −1.61(−1.81-−1.40) | |  | |
| Côte d'Ivoire | 2.28(1.55-3.27) | | | 47.59 | | −2.89(−3.23-−2.55) | | 0.96(0.62-1.42) | | 82.23 | | −1.73(−1.93-−1.52) | |  | |
| Democratic People's Republic of Korea | 5.84(4.08-8.10) | | | 22.84 | | −1.58(−1.7-−1.47) | | 2.44(1.60-3.41) | | 52.79 | | −1.45(−1.53-−1.36) | |  | |
| Democratic Republic of the Congo | 0.49(0.32-0.76) | | | 93.76 | | −0.82(−0.93-−0.70) | | 1.17(0.85-1.55) | | 106.11 | | −0.52(−0.59-−0.45) | |  | |
| Denmark | 0.45(0.30-0.65) | | | 142.38 | | 2.36(2.11-2.60) | | 1.15(0.85-1.49) | | 140.26 | | 2.21(1.96-2.45) | |  | |
| Djibouti | 0.98(0.55-1.74) | | | 285.09 | | −0.15(−0.31-0.01) | | 0.92(0.57-1.47) | | 340.67 | | 0.04(−0.06-0.13) | |  | |
| Dominica | 0.84(0.56-1.19) | | | −56.08 | | −3.88(−4.65-−3.11) | | 0.79(0.52-1.11) | | −58.10 | | −3.88(−4.65-−3.09) | |  | |
| Dominican Republic | 1.30(0.77-2.15) | | | 153.97 | | 0.47(−0.08-1.02) | | 1.12(0.72-1.72) | | 196.13 | | 0.70(0.19-1.21) | |  | |
| Ecuador | 1.49(1.04-2.03) | | | 186.91 | | 0.50(0.21-0.79) | | 0.35(0.21-0.52) | | 270.39 | | 1.41(1.02-1.80) | |  | |
| Egypt | 2.41(1.46-3.89) | | | 132.94 | | 0.72(0.47-0.97) | | 14.05(9.83-19.71) | | 156.77 | | 1.48(1.12-1.84) | |  | |
| El Salvador | 0.31(0.19-0.47) | | | −9.46 | | −2.79(−3.45-−2.13) | | 0.84(0.57-1.18) | | 19.14 | | −2.19(−2.92-−1.45) | |  | |
| Equatorial Guinea | 0.77(0.41-1.25) | | | 150.21 | | −0.18(−0.40-0.05) | | 1.61(0.93-2.47) | | 123.06 | | −0.11(−0.33-0.11) | |  | |
| Eritrea | 0.87(0.52-1.37) | | | 126.10 | | −0.93(−1.13-−0.74) | | 0.98(0.62-1.44) | | 177.81 | | 0.01(−0.14-0.15) | |  | |
| Estonia | 0.67(0.43-0.99) | | | 55.33 | | −0.25(−0.91-0.41) | | 0.91(0.59-1.29) | | 114.12 | | 0.61(0.04-1.18) | |  | |
| Eswatini | 7.43(1.82-14.12) | | | 622.43 | | 5.37(3.92-6.85) | | 4.46(1.64-8.17) | | 384.05 | | 3.97(2.98-4.97) | |  | |
| Ethiopia | 0.60(0.44-0.81) | | | 78.91 | | −0.53(−0.65-−0.41) | | 1.21(0.95-1.55) | | 110.87 | | −0.18(−0.27-−0.09) | |  | |
| Fiji | 2.94(2.10-4.07) | | | 98.70 | | 0.58(0.13-1.03) | | 1.50(1.02-2.14) | | 119.69 | | 0.47(0.17-0.77) | |  | |
| Finland | 0.51(0.35-0.73) | | | 110.18 | | 1.22(1.09-1.34) | | 1.52(1.13-1.94) | | 151.81 | | 1.40(1.30-1.50) | |  | |
| France | 0.76(0.50-1.11) | | | 69.41 | | 0.01(−0.24-0.26) | | 2.45(1.84-3.11) | | 118.35 | | 0.60(0.31-0.89) | |  | |
| Gabon | 0.68(0.40-1.10) | | | 80.07 | | −0.32(−0.53-−0.12) | | 1.77(1.15-2.59) | | 70.21 | | −0.37(−0.50-−0.25) | |  | |
| Gambia | 20.34(14.26-28.33) | | | 218.08 | | 0.23(0.05-0.41) | | 6.04(3.83-8.89) | | 284.27 | | 0.80(0.63-0.97) | |  | |
| Georgia | 0.87(0.60-1.25) | | | 190.53 | | 3.32(2.01-4.64) | | 1.16(0.83-1.54) | | 170.41 | | 2.38(1.22-3.55) | |  | |
| Germany | 0.40(0.27-0.59) | | | 96.35 | | 1.04(0.66-1.42) | | 1.17(0.87-1.49) | | 146.68 | | 1.65(1.22-2.09) | |  | |
| Ghana | 2.83(1.99-3.86) | | | 137.83 | | −0.62(−0.83-−0.42) | | 1.02(0.66-1.46) | | 128.09 | | −0.62(−0.74-−0.50) | |  | |
| Greece | 1.13(0.85-1.48) | | | 110.03 | | 1.48(1.31-1.65) | | 0.59(0.41-0.80) | | 138.88 | | 1.07(0.97-1.18) | |  | |
| Greenland | 0.76(0.47-1.15) | | | 111.39 | | 0.92(0.83-1.00) | | 1.74(1.15-2.52) | | 185.67 | | 0.90(0.69-1.11) | |  | |
| Grenada | 0.74(0.52-1.02) | | | −51.70 | | −4.64(−5.59-−3.69) | | 0.73(0.50-1.00) | | −58.62 | | −4.19(−5.15-−3.22) | |  | |
| Guam | 3.33(2.54-4.24) | | | 278.07 | | 2.43(2.20-2.66) | | 0.96(0.63-1.40) | | 236.09 | | 0.41(0.22-0.61) | |  | |
| Guatemala | 0.74(0.48-1.10) | | | 21.99 | | −3.90(−5.27-−2.50) | | 2.13(1.55-2.85) | | 55.15 | | −3.49(−4.95-−2.01) | |  | |
| Guinea | 17.49(11.6-23.83) | | | 80.12 | | 0.31(0.22-0.40) | | 6.04(3.67-8.90) | | 64.73 | | 0.08(0.04-0.12) | |  | |
| Guinea-Bissau | 2.91(1.99-4.10) | | | 27.29 | | −1.37(−1.46-−1.29) | | 1.36(0.84-2.09) | | 39.94 | | −0.72(−0.79-−0.66) | |  | |
| Guyana | 0.82(0.55-1.18) | | | −30.13 | | −2.87(−3.60-−2.14) | | 0.75(0.49-1.06) | | −24.26 | | −2.73(−3.53-−1.92) | |  | |
| Haiti | 1.22(0.68-2.08) | | | 34.25 | | −1.74(−1.97-−1.51) | | 1.10(0.60-1.73) | | 25.3 | | −1.97(−2.19-−1.76) | |  | |
| Honduras | 2.53(0.98-4.37) | | | 214.95 | | 0.47(0.34-0.60) | | 6.82(2.82-10.85) | | 300.86 | | 1.39(1.18-1.60) | |  | |
| Hungary | 0.58(0.39-0.83) | | | −51.36 | | −2.54(−3.36-−1.70) | | 0.64(0.43-0.90) | | −45.78 | | −2.54(−3.33-−1.76) | |  | |
| Iceland | 0.42(0.29-0.61) | | | 165.29 | | 1.18(1.04-1.31) | | 1.03(0.76-1.35) | | 177.71 | | 1.13(0.97-1.29) | |  | |
| India | 0.91(0.74-1.12) | | | 120.16 | | −0.26(−0.39-−0.12) | | 0.61(0.47-0.77) | | 209.8 | | 0.16(0.03-0.29) | |  | |
| Indonesia | 0.43(0.34-0.53) | | | 69.75 | | −0.69(−0.84-−0.55) | | 1.22(1.02-1.39) | | 101.66 | | −0.32(−0.41-−0.23) | |  | |
| Iran  (Islamic Republic of) | 1.32(1.13-1.55) | | | 112.85 | | −1.38(−2-−0.75) | | 1.12(0.94-1.31) | | 164.95 | | −1.19(−1.85-−0.53) | |  | |
| Iraq | 1.97(1.34-2.76) | | | 267.34 | | 0.89(0.49-1.29) | | 2.43(1.70-3.31) | | 276.29 | | 1.35(0.90-1.81) | |  | |
| Ireland | 0.41(0.28-0.60) | | | 283.86 | | 3.52(3.15-3.89) | | 1.31(0.99-1.68) | | 324.27 | | 3.65(3.32-3.99) | |  | |
| Israel | 0.41(0.28-0.59) | | | 125.41 | | −0.21(−0.32-−0.10) | | 1.25(0.97-1.56) | | 144.6 | | −0.08(−0.20-0.05) | |  | |
| Italy | 0.52(0.43-0.64) | | | −4.77 | | −2.19(−2.55-−1.83) | | 2.66(2.34-2.93) | | 29.19 | | −1.38(−1.63-−1.14) | |  | |
| Jamaica | 0.71(0.48-1.00) | | | 1.38 | | −1.08(−1.98-−0.17) | | 0.65(0.43-0.94) | | 9.18 | | −0.86(−1.73-0.02) | |  | |
| Japan | 1.05(0.89-1.24) | | | 14.79 | | −2.63(−3.12-−2.14) | | 6.08(5.30-6.63) | | 91.82 | | −1.64(−2.21-−1.07) | |  | |
| Jordan | 0.77(0.53-1.08) | | | 224.32 | | −1.65(−1.79-−1.50) | | 0.88(0.61-1.19) | | 257.61 | | −1.34(−1.48-−1.20) | |  | |
| Kazakhstan | 1.40(0.96-1.98) | | | 122.67 | | −2.01(−3.23-−0.77) | | 2.30(1.62-3.05) | | 152.49 | | −0.65(−1.64-0.34) | |  | |
| Kenya | 0.81(0.52-1.20) | | | 236.29 | | 0.06(−0.39-0.52) | | 0.86(0.58-1.23) | | 229.19 | | 0.49(0.19-0.79) | |  | |
| Kiribati | 5.97(4.30-8.02) | | | 50.33 | | −0.97(−1.04-−0.89) | | 3.00(1.93-4.33) | | 58.89 | | −0.36(−0.43-−0.30) | |  | |
| Kuwait | 0.73(0.48-1.07) | | | 167.35 | | −0.41(−0.75-−0.06) | | 0.89(0.63-1.20) | | 268.48 | | 0.41(0.09-0.72) | |  | |
| Kyrgyzstan | 0.54(0.38-0.77) | | | 191.94 | | 2.89(2.44-3.34) | | 1.06(0.77-1.37) | | 181.98 | | 3.33(2.98-3.67) | |  | |
| Lao People's Democratic Republic | 2.64(1.79-3.70) | | | 29.89 | | −1.92(−2.00-−1.83) | | 1.98(1.31-2.75) | | 33.27 | | −1.71(−1.84-−1.57) | |  | |
| Latvia | 0.53(0.36-0.77) | | | 31.35 | | −0.19(−1.09-0.72) | | 0.69(0.46-0.95) | | 72.92 | | 0.54(−0.19-1.29) | |  | |
| Lebanon | 1.45(1.00-2.06) | | | 79.62 | | −0.81(−0.91-−0.71) | | 0.70(0.44-1.11) | | 113.73 | | −0.79(−0.87-−0.72) | |  | |
| Lesotho | 5.62(1.95-9.50) | | | 292.41 | | 4.02(3.17-4.88) | | 3.97(1.91-6.49) | | 188.33 | | 3.34(2.84-3.85) | |  | |
| Liberia | 2.38(1.57-3.65) | | | 33.30 | | −1.54(−1.70-−1.38) | | 1.09(0.67-1.64) | | 30.87 | | −1.14(−1.27-−1.01) | |  | |
| Libya | 1.71(1.14-2.50) | | | 148.11 | | −0.55(−0.64-−0.45) | | 1.83(1.21-2.66) | | 155.78 | | −0.17(−0.31-−0.02) | |  | |
| Lithuania | 0.61(0.40-0.90) | | | 73.45 | | 0.48(0.03-0.93) | | 0.73(0.48-1.00) | | 110.39 | | 0.85(0.49-1.21) | |  | |
| Luxembourg | 0.41(0.26-0.63) | | | 103.27 | | 0.33(0.03-0.63) | | 1.37(0.98-1.88) | | 141.57 | | 0.96(0.72-1.20) | |  | |
| Madagascar | 0.74(0.43-1.25) | | | 70.49 | | −1.19(−1.32-−1.07) | | 0.69(0.45-1.05) | | 81.77 | | −0.38(−0.48-−0.27) | |  | |
| Malawi | 0.75(0.50-1.10) | | | 40.32 | | −2.14(−2.63-−1.66) | | 0.77(0.52-1.07) | | 69.70 | | −0.86(−1.18-−0.55) | |  | |
| Malaysia | 3.36(2.39-4.44) | | | 189.49 | | 0.24(−0.02-0.49) | | 1.12(0.73-1.65) | | 206.44 | | 0.31(−0.08-0.70) | |  | |
| Maldives | 2.08(1.45-2.84) | | | 143.81 | | −1.36(−1.51-−1.2) | | 1.58(1.04-2.20) | | 196.97 | | −0.71(−0.82-−0.59) | |  | |
| Mali | 5.43(3.47-7.84) | | | 90.81 | | −0.55(−0.64-−0.46) | | 5.28(3.56-7.22) | | 95.10 | | −0.34(−0.44-−0.24) | |  | |
| Malta | 0.29(0.19-0.42) | | | 140.90 | | 0.63(0.36-0.91) | | 0.85(0.63-1.10) | | 190.17 | | 0.88(0.65-1.12) | |  | |
| Marshall Islands | 5.38(3.58-7.88) | | | 86.68 | | −0.56(−0.63-−0.48) | | 2.47(1.52-3.65) | | 70.97 | | −0.59(−0.66-−0.53) | |  | |
| Mauritania | 2.02(1.39-2.83) | | | 11.04 | | −2.16(−2.30-−2.01) | | 0.94(0.61-1.36) | | 25.60 | | −1.64(−1.76-−1.52) | |  | |
| Mauritius | 0.64(0.43-0.93) | | | 106.28 | | 0.98(0.63-1.34) | | 0.59(0.39-0.83) | | 167.13 | | 1.42(1.07-1.76) | |  | |
| Mexico | 0.40(0.32-0.50) | | | 241.76 | | 0.85(0.65-1.05) | | 1.56(1.31-1.86) | | 315.76 | | 1.26(1.09-1.44) | |  | |
| Micronesia  (Federated States of) | 5.31(3.29-7.72) | | | 41.02 | | −0.46(−0.62-−0.29) | | 2.53(1.54-3.79) | | 33.28 | | −0.37(−0.51-−0.22) | |  | |
| Monaco | 1.02(0.66-1.50) | | | 184.08 | | 3.04(2.27-3.81) | | 2.93(2.06-3.93) | | 221.66 | | 3.73(2.9-4.57) | |  | |
| Mongolia | 28.23(18.92-40.83) | | | 197.77 | | 1.32(0.97-1.67) | | 40.31(28.58-53.28) | | 223.70 | | 3.19(2.57-3.82) | |  | |
| Montenegro | 1.28(0.85-1.84) | | | 33.96 | | −0.36(−0.72-0) | | 1.55(1.07-2.18) | | 68.20 | | −0.02(−0.26-0.22) | |  | |
| Morocco | 0.74(0.49-1.06) | | | 130.14 | | −0.21(−0.44-0.02) | | 0.88(0.60-1.19) | | 161.45 | | 0.26(−0.01-0.52) | |  | |
| Mozambique | 1.49(0.95-2.15) | | | 228.87 | | 1.75(1.5-2.01) | | 0.72(0.45-1.07) | | 177.32 | | 1.34(1.19-1.50) | |  | |
| Myanmar | 1.35(0.95-1.88) | | | 158.56 | | 0.89(0.69-1.09) | | 1.46(1.02-1.93) | | 200.95 | | 1.16(0.97-1.36) | |  | |
| Namibia | 1.08(0.72-1.55) | | | 241.03 | | 1.97(1.44-2.49) | | 1.05(0.71-1.44) | | 204.33 | | 1.52(1.25-1.80) | |  | |
| Nauru | 4.60(3.09-6.58) | | | −3.89 | | −0.89(−1.21-−0.57) | | 2.18(1.38-3.28) | | −8.87 | | −0.63(−0.89-−0.36) | |  | |
| Nepal | 0.49(0.30-0.78) | | | 128.72 | | 0.09(0.01-0.18) | | 0.87(0.58-1.30) | | 212.14 | | 0.79(0.65-0.93) | |  | |
| Netherlands | 0.34(0.24-0.48) | | | 208.09 | | 2.54(2.39-2.69) | | 0.92(0.69-1.17) | | 252.02 | | 2.66(2.54-2.78) | |  | |
| New Zealand | 0.61(0.51-0.73) | | | 197.95 | | 1.96(1.8-2.13) | | 1.11(0.95-1.27) | | 286.19 | | 2.36(2.21-2.51) | |  | |
| Nicaragua | 0.55(0.36-0.81) | | | 158.70 | | 0.02(−0.41-0.44) | | 1.72(1.27-2.25) | | 260.19 | | 0.98(0.69-1.27) | |  | |
| Niger | 0.31(0.21-0.43) | | | 136.84 | | −0.58(−0.67-−0.5) | | 0.14(0.09-0.20) | | 171.63 | | −0.28(−0.36-−0.20) | |  | |
| Nigeria | 1.30(0.99-1.70) | | | 74.88 | | −0.13(−0.21-−0.05) | | 0.86(0.66-1.07) | | 81.79 | | −0.01(−0.1-0.08) | |  | |
| Niue | 3.54(2.52-4.84) | | | −13.96 | | −0.74(−0.82-−0.66) | | 1.66(1.09-2.40) | | −21.69 | | −0.69(−0.72-−0.65) | |  | |
| North Macedonia | 1.97(1.30-2.90) | | | 42.94 | | −0.65(−0.87-−0.43) | | 2.55(1.73-3.55) | | 75.78 | | 0(−0.12-0.12) | |  | |
| Northern Mariana | 4.26(3.21-5.54) | | | 196.28 | | 1.06(0.85-1.27) | | 1.54(1.00-2.13) | | 224.01 | | −0.03(−0.21-0.15) | |  | |
| Islands |  | | |  | |  | |  | |  | |  | |  | |
| Norway | 0.33(0.27-0.41) | | | 95.93 | | 1.54(1.33-1.74) | | 0.99(0.85-1.13) | | 123.04 | | 2.03(1.87-2.18) | |  | |
| Oman | 1.60(1.13-2.24) | | | 182.83 | | 0.63(0.22-1.04) | | 1.76(1.23-2.30) | | 143.54 | | 0.88(0.52-1.23) | |  | |
| Pakistan | 0.51(0.39-0.66) | | | 112.91 | | −0.04(−0.22-0.14) | | 1.94(1.48-2.50) | | 77.32 | | −0.16(−0.3-−0.02) | |  | |
| Palau | 5.55(4.02-7.45) | | | 117.50 | | −0.17(−0.24-−0.1) | | 1.69(1.11-2.38) | | 112.01 | | 0.01(−0.05-0.07) | |  | |
| Palestine | 2.02(1.46-2.75) | | | 102.61 | | −1.28(−1.41-−1.16) | | 2.89(2.13-3.75) | | 79.08 | | −1.05(−1.13-−0.98) | |  | |
| Panama | 0.44(0.28-0.66) | | | 91.31 | | −0.88(−1.25-−0.51) | | 1.13(0.77-1.59) | | 143.19 | | −0.23(−0.60-0.14) | |  | |
| Papua New Guinea | 0.72(0.50-1.00) | | | 159.34 | | 0.24(0.21-0.27) | | 0.51(0.34-0.73) | | 166.90 | | 0.36(0.33-0.39) | |  | |
| Paraguay | 0.36(0.23-0.56) | | | 119.37 | | −0.11(−0.62-0.41) | | 0.85(0.58-1.17) | | 121.81 | | −0.22(−0.76-0.32) | |  | |
| Peru | 1.10(0.74-1.58) | | | 2.10 | | −3.98(−4.54-−3.42) | | 0.22(0.13-0.34) | | 39.68 | | −3.39(−3.94-−2.83) | |  | |
| Philippines | 2.69(2.10-3.37) | | | 48.85 | | −2.26(−2.54-−1.99) | | 1.56(1.26-1.92) | | 86.23 | | −1.61(−1.91-−1.31) | |  | |
| Poland | 0.39(0.31-0.50) | | | −63.81 | | −5.02(−6.23-−3.8) | | 0.51(0.41-0.62) | | −61.01 | | −5.02(−6.37-−3.66) | |  | |
| Portugal | 0.63(0.43-0.91) | | | 255.08 | | 2.69(2.30-3.07) | | 1.63(1.21-2.09) | | 307.81 | | 2.64(2.31-2.97) | |  | |
| Puerto Rico | 0.67(0.43-0.99) | | | −33.29 | | −2.27(−2.99-−1.54) | | 0.62(0.4-0.91) | | −20.34 | | −2.4(−3.2-−1.58) | |  | |
| Qatar | 4.36(2.71-6.73) | | | 512.64 | | −0.81(−1.01-−0.61) | | 6.48(4.41-9.04) | | 492.81 | | 0.04(−0.2-0.29) | |  | |
| Republic of Korea | 8.76(7.19-10.42) | | | 254.74 | | 1.71(0.39-3.04) | | 3.08(2.18-4.04) | | 455.35 | | 2.07(0.98-3.17) | |  | |
| Republic of Moldova | 0.42(0.29-0.61) | | | 65.50 | | −1.53(−3.26-0.24) | | 0.57(0.39-0.79) | | 121.10 | | −0.14(−1.58-1.31) | |  | |
| Romania | 0.63(0.43-0.90) | | | 76.57 | | 1.90(1.46-2.35) | | 0.74(0.50-1.03) | | 122.15 | | 2.17(1.76-2.57) | |  | |
| Russian Federation | 0.68(0.53-0.88) | | | 84.33 | | 1.76(1.44-2.08) | | 0.80(0.66-0.97) | | 132.41 | | 2.39(2.09-2.68) | |  | |
| Rwanda | 1.13(0.74-1.62) | | | 72.84 | | −1.50(−1.79-−1.2) | | 1.19(0.82-1.67) | | 86.69 | | −0.77(−0.99-−0.55) | |  | |
| Saint Kitts and Nevis | 1.01(0.68-1.43) | | | −47.82 | | −5.11(−6.11-−4.09) | | 0.93(0.62-1.28) | | −59.06 | | −4.40(−5.38-−3.42) | |  | |
| Saint Lucia | 0.55(0.38-0.76) | | | −20.28 | | −3.98(−4.96-−2.98) | | 0.51(0.35-0.72) | | −17.64 | | −4.22(−5.29-−3.14) | |  | |
| Saint Vincent and the Grenadines | 0.81(0.58-1.09) | | | −30.77 | | −3.37(−4.12-−2.61) | | 0.67(0.46-0.93) | | −34.25 | | −3.38(−4.3-−2.45) | |  | |
| Samoa | 2.66(1.93-3.59) | | | 34.85 | | −0.79(−0.89-−0.69) | | 1.07(0.69-1.55) | | 38.55 | | −0.62(−0.74-−0.51) | |  | |
| San Marino | 0.35(0.19-0.58) | | | 120.08 | | 1.08(0.87-1.29) | | 1.00(0.61-1.53) | | 168.40 | | 1.48(1.24-1.72) | |  | |
| Sao Tome and Principe | 1.24(0.78-1.75) | | | 79.41 | | −0.15(−0.25-−0.05) | | 0.53(0.32-0.82) | | 53.20 | | 0(−0.12-0.13) | |  | |
| Saudi Arabia | 1.54(1.01-2.25) | | | 105.12 | | −1.80(−2.03-−1.56) | | 1.97(1.36-2.71) | | 118.48 | | −0.72(−1.01-−0.44) | |  | |
| Senegal | 1.23(0.87-1.62) | | | 105.60 | | −0.44(−0.56-−0.32) | | 0.23(0.14-0.35) | | 121.49 | | 0.06(−0.07-0.20) | |  | |
| Serbia | 1.04(0.67-1.56) | | | −3.30 | | −1.30(−1.69-−0.92) | | 1.87(1.30-2.56) | | 36.05 | | −0.57(−0.83-−0.32) | |  | |
| Seychelles | 2.03(1.48-2.71) | | | 29.30 | | −1.70(−1.99-−1.40) | | 1.44(1.00-1.93) | | 22.19 | | −1.10(−1.36-−0.84) | |  | |
| Sierra Leone | 2.21(1.52-3.14) | | | 42.89 | | −1.18(−1.28-−1.08) | | 1.05(0.66-1.51) | | 48.71 | | −0.59(−0.64-−0.55) | |  | |
| Singapore | 4.65(3.62-5.71) | | | 215.79 | | −0.28(−0.58-0.02) | | 2.65(1.77-3.55) | | 376.34 | | 0.87(0.57-1.17) | |  | |
| Slovakia | 0.66(0.42-0.99) | | | −7.21 | | −2.31(−2.7-−1.92) | | 0.82(0.54-1.18) | | 19.55 | | −1.43(−1.77-−1.08) | |  | |
| Slovenia | 1.13(0.71-1.70) | | | 159.14 | | 1.62(1.17-2.09) | | 1.33(0.86-1.90) | | 215.54 | | 2.17(1.86-2.48) | |  | |
| Solomon Islands | 2.73(2.05-3.57) | | | 66.89 | | −1.04(−1.14-−0.94) | | 1.27(0.86-1.75) | | 86.49 | | −0.41(−0.49-−0.33) | |  | |
| Somalia | 1.13(0.67-2.12) | | | 139.89 | | −0.29(−0.35-−0.22) | | 0.99(0.59-1.76) | | 161.4 | | 0.24(0.17-0.31) | |  | |
| South Africa | 1.94(1.63-2.29) | | | 94.49 | | −0.68(−1.33-−0.02) | | 1.70(1.45-2.04) | | 99.18 | | −0.59(−1.14-−0.03) | |  | |
| South Sudan | 0.89(0.46-1.69) | | | 45.91 | | −0.42(−0.49-−0.35) | | 0.78(0.44-1.30) | | 41.45 | | −0.18(−0.23-−0.13) | |  | |
| Spain | 0.56(0.37-0.81) | | | 102.8 | | 0.55(0.25-0.84) | | 2.70(2.10-3.30) | | 148.12 | | 0.91(0.64-1.19) | |  | |
| Sri Lanka | 0.98(0.64-1.44) | | | 157.54 | | 1.35(0.94-1.76) | | 0.77(0.50-1.12) | | 232.61 | | 1.82(1.51-2.14) | |  | |
| Sudan | 1.27(0.71-2.07) | | | 87.62 | | −0.27(−0.37-−0.17) | | 1.24(0.72-1.96) | | 89.48 | | −0.09(−0.21-0.03) | |  | |
| Suriname | 0.71(0.49-1.00) | | | −17.93 | | −3.47(−4.19-−2.75) | | 0.63(0.43-0.90) | | −8.50 | | −3.13(−3.9-−2.35) | |  | |
| Sweden | 0.18(0.14-0.24) | | | 56.08 | | 1.18(0.58-1.78) | | 1.08(0.91-1.27) | | 50.44 | | 0.82(0.23-1.42) | |  | |
| Switzerland | 0.59(0.39-0.85) | | | 207.98 | | 1.67(1.11-2.23) | | 1.58(1.15-2.08) | | 242.13 | | 2.03(1.51-2.55) | |  | |
| Syrian Arab Republic | 1.40(0.92-2.04) | | | 74.500 | | −1.18(−1.39-−0.97) | | 1.75(1.22-2.40) | | 100.28 | | −0.61(−0.77-−0.45) | |  | |
| Taiwan  (Province of China) | 3.08(2.22-4.17) | | | 6.09 | | −3.58(−4.29-−2.86) | | 2.60(1.81-3.56) | | 107.69 | | −1.63(−2.62-−0.64) | |  | |
| Tajikistan | 0.63(0.42-0.89) | | | 592.56 | | 5.04(4.79-5.29) | | 2.10(1.57-2.75) | | 477.45 | | 5.64(5.35-5.93) | |  | |
| Thailand | 9.50(6.5-13.47) | | | 151.59 | | −0.13(−0.22-−0.04) | | 4.30(2.73-6.36) | | 233.18 | | 0.38(0.22-0.55) | |  | |
| Timor−Leste | 2.54(1.42-3.92) | | | 90.62 | | −1.04(−1.21-−0.87) | | 1.94(1.27-2.81) | | 166.52 | | −0.74(−0.90-−0.58) | |  | |
| Togo | 2.36(1.68-3.25) | | | 111.73 | | −1.59(−1.81-−1.37) | | 1.13(0.74-1.60) | | 125.60 | | −1.04(−1.15-−0.94) | |  | |
| Tokelau | 3.74(2.43-5.33) | | | −11.71 | | −0.55(−0.59-−0.51) | | 1.78(1.18-2.53) | | −25.77 | | −0.89(−0.93-−0.84) | |  | |
| Tonga | 12.87(8.72-18.05) | | | 34.55 | | −0.11(−0.2-−0.02) | | 5.15(3.28-7.50) | | 53.76 | | 0.06(−0.07-0.19) | |  | |
| Trinidad and Tobago | 0.65(0.42-0.96) | | | −27.98 | | −3.96(−4.92-−2.99) | | 0.63(0.40-0.92) | | −17.84 | | −3.77(−4.78-−2.74) | |  | |
| Tunisia | 0.48(0.29-0.74) | | | 98.38 | | −0.69(−0.79-−0.59) | | 0.89(0.60-1.30) | | 145.30 | | −0.12(−0.20-−0.05) | |  | |
| Turkey | 1.20(0.86-1.63) | | | 58.35 | | −1.54(−1.67-−1.41) | | 0.86(0.58-1.14) | | 112.81 | | −0.63(−0.91-−0.35) | |  | |
| Turkmenistan | 1.40(0.94-1.99) | | | 1097.66 | | 6.76(5.51-8.03) | | 1.79(1.19-2.44) | | 721.92 | | 5.14(4.22-6.07) | |  | |
| Tuvalu | 4.43(3.08-6.32) | | | 15.25 | | −0.86(−1-−0.72) | | 2.07(1.30-3.00) | | 17.99 | | −1.06(−1.19-−0.92) | |  | |
| Uganda | 1.63(1.10-2.35) | | | 183.19 | | 0.58(0.36-0.79) | | 1.46(0.98-2.04) | | 208.66 | | 1.50(1.30-1.70) | |  | |
| Ukraine | 0.64(0.51-0.81) | | | 179.75 | | 4.49(3.76-5.23) | | 0.73(0.60-0.89) | | 154.51 | | 3.40(2.98-3.82) | |  | |
| United Arab Emirates | 1.72(0.68-4.20) | | | 999.18 | | 0.15(0.02-0.29) | | 1.70(0.67-4.26) | | 823.27 | | −0.06(−0.43-0.03) | |  | |
| United Kingdom | 0.53(0.44-0.63) | | | 182.43 | | 3.16(2.96-3.36) | | 1.55(1.36-1.73) | | 214.12 | | 3.40(3.19-3.62) | |  | |
| United Republic of Tanzania | 0.58(0.39-0.84) | | | 129.48 | | −0.08(−0.24-0.09) | | 0.62(0.42-0.84) | | 155.67 | | 0.33(0.21-0.45) | |  | |
| United States of America | 0.53(0.43-0.65) | | | 223.58 | | 2.36(2.15-2.56) | | 1.63(1.40-1.87) | | 278.89 | | 2.77(2.56-2.98) | |  | |
| United States Virgin Islands | 0.66(0.45-0.93) | | | 8.73 | | −2.06(−2.68-−1.43) | | 0.58(0.39-0.79) | | 35.37 | | −2.15(−2.70-−1.60) | |  | |
| Uruguay | 0.42(0.29-0.59) | | | 98.44 | | 1.73(1.54-1.92) | | 0.85(0.61-1.10) | | 134.69 | | 2.09(1.94-2.24) | |  | |
| Uzbekistan | 1.50(1.04-2.11) | | | 1378.88 | | 9.53(8.31-10.77) | | 2.48(1.80-3.26) | | 973.59 | | 9.03(8.04-10.03) | |  | |
| Vanuatu | 4.86(3.15-7.21) | | | 145.85 | | −0.21(−0.34-−0.07) | | 2.12(1.27-3.15) | | 160.99 | | −0.13(−0.24-−0.02) | |  | |
| Venezuela | 0.34(0.22-0.53) | | | −6.69 | | −3.19(−4.37-−2.00) | | 0.90(0.62-1.25) | | 7.79 | | −2.97(−4.19-−1.72) | |  | |
| Viet Nam | 1.00(0.68-1.42) | | | 30.83 | | −2.22(−2.48-−1.96) | | 0.72(0.43-1.11) | | 51.21 | | −1.35(−1.51-−1.19) | |  | |
| Yemen | 0.98(0.61-1.51) | | | 148.63 | | −0.30(−0.37-−0.22) | | 1.66(1.13-2.33) | | 171.05 | | 0.05(−0.05-0.15) | |  | |
| Zambia | 0.77(0.52-1.10) | | | 164.34 | | −0.44(−0.9-0.01) | | 0.75(0.51-1.04) | | 168.07 | | −0.04(−0.35-0.26) | |  | |
| Zimbabwe | 4.49(2.96-6.87) | | | 76.00 | | −1.35(−2.04-−0.65) | | 5.36(3.65-7.57) | | 130.34 | | 0.52(0.12-0.93) | |  | |

LCHB, liver cancer due to hepatitis B; LCHC, liver cancer due to hepatitis C; LCAL, liver cancer due to alcohol use; LCNA, liver cancer due to non-alcoholic steatohepatitis; EAPC: estimated annual percentage change; CI, confidence interval.

**Supplementary table 4.** The percentage change in number and the EAPCs of death attribute to liver cancer caused by specific etiologies in global, sexes, SDI areas and geographic regions from 1990 to 2019.

|  | **LCAL** | | | | **LCNA** | | |
| --- | --- | --- | --- | --- | --- | --- | --- |
| **Characteristics** | Number in 2019  ×10^3^ (95% UI) | Changes in number (%) | EAPCs  (95%CI) | Number in 2019  ×10^3^ (95% UI) | | Changes in number (%) | EAPC  (95%CI) |
| **Overall** | 90.74(73.35-109.4) | 89.60 | −0.68(−0.87-−0.49) | 34.73(28.39-43.18) | | 95.10 | −0.74(−1.02-−0.46) |
| **Sex** |  |  |  |  | |  |  |
| Male | 71.67(58.14-86.84) | 100.72 | −0.52(−0.70-−0.34) | 17.38(13.97-21.99) | | 109.00 | −0.63(−0.95-−0.31) |
| Female | 19.07(14.45-24.11) | 56.95 | −1.34(−1.55-−1.13) | 17.35(13.81-21.79) | | 82.91 | −0.84(−1.09-−0.59) |
| **SDI** |  |  |  |  | |  |  |
| Low | 4.16(3.12-5.31) | −73.74 | −0.17(−0.23-−0.10) | 1.80(1.37-2.37) | | 133.08 | 0.03(−0.02-0.08) |
| Low-middle | 11.57(9.17-14.15) | 497.18 | −0.33(−0.49-−0.16) | 4.84(3.89-6.06) | | 128.20 | −0.38(−0.57-−0.19) |
| Middle | 29.45(22.18-37.36) | 108.39 | −1.41(−1.8-−1.03) | 13.68(10.95-17.31) | | 84.58 | −1.54(−1.99-−1.08) |
| High-middle | 18.20(14.80-21.80) | 68.10 | −1.90(−2.18-−1.62) | 6.15(4.98-7.56) | | 29.57 | −2.25(−2.69-−1.80) |
| High | 27.32(22.49-32.05) | 435.54 | 1.03(0.79-1.26) | 8.24(6.48-10.35) | | 200.58 | 1.45(1.09-1.81) |
| **Regions** |  |  |  |  | |  |  |
| East Asia | 17.97(13.15-23.27) | 0.34 | −4.40(−5.19-−3.60) | 9.79(7.87-12.10) | | 7.89 | −4.10(−4.86-−3.32) |
| South Asia | 10.08(8.09-12.26) | 180.46 | 0.27(0.18-0.37) | 4.16(3.35-5.05) | | 196.11 | 0.40(0.29-0.51) |
| Southeast Asia | 11.18(7.88-15.39) | 208.50 | 1.07(0.96-1.18) | 4.08(2.9-5.63) | | 227.83 | 1.31(1.21-1.42) |
| Central Asia | 1.86(1.31-2.51) | 361.47 | 2.94(2.41-3.46) | 0.44(0.31-0.62) | | 433.18 | 4.14(3.64-4.65) |
| High-income Asia Pacific | 6.22(4.96-7.73) | 104.17 | −0.78(−1.31-−0.24) | 2.52(1.92-3.19) | | 162.15 | −0.34(−0.97-0.29) |
| Oceania | 0.03(0.02-0.04) | 128.49 | 0.19(0.11-0.26) | 0.02(0.01-0.03) | | 146.59 | 0.26(0.20-0.32) |
| Australasia | 0.77(0.60-0.93) | 288.29 | 2.65(2.42-2.88) | 0.22(0.16-0.30) | | 521.72 | 4.02(3.73-4.31) |
| Eastern Europe | 3.64(3.00-4.38) | 157.31 | 2.97(2.66-3.28) | 0.82(0.68-1.00) | | 171.28 | 3.14(2.90-3.37) |
| Western Europe | 14.02(11.22-16.81) | 102.76 | 0.97(0.82-1.12) | 2.75(2.01-3.73) | | 147.11 | 1.49(1.31-1.66) |
| Central Europe | 3.09(2.40-3.85) | −2.10 | −1.05(−1.45-−0.65) | 0.60(0.43-0.82) | | 0.26 | −1.24(−1.68-−0.80) |
| High-income North America | 8.86(7.23-10.43) | 298.38 | 2.78(2.72-2.84) | 2.89(2.35-3.50) | | 300.11 | 3.03(2.84-3.22) |
| Andean Latin America | 0.59(0.41-0.82) | 89.10 | −1.70(−2.20-−1.19) | 0.20(0.13-0.28) | | 137.03 | −1.05(−1.44-−0.65) |
| Central Latin America | 2.59(2.00-3.26) | 200.56 | 0.33(−0.03-0.69) | 0.78(0.61-1.01) | | 258.20 | 0.95(0.65-1.24) |
| Caribbean | 0.59(0.43-0.80) | 11.05 | −1.93(−2.73-−1.12) | 0.17(0.11-0.23) | | 19.39 | −1.67(−2.53-−0.80) |
| Tropical Latin America | 1.82(1.57-2.07) | 252.03 | 1.41(1.26-1.55) | 0.38(0.32-0.45) | | 267.04 | 1.54(1.32-1.76) |
| Southern Latin America | 0.65(0.47-0.83) | 159.54 | 1.96(1.68-2.25) | 0.20(0.14-0.29) | | 247.50 | 2.83(2.62-3.05) |
| Eastern Sub-Saharan Africa | 1.38(0.98-1.93) | 134.33 | 0.11(−0.04-0.26) | 0.63(0.46-0.84) | | 163.00 | 0.52(0.42-0.63) |
| Southern Sub-Saharan Africa | 0.80(0.63-0.99) | 118.93 | −0.50(−1.17-0.18) | 0.43(0.35-0.53) | | 144.75 | 0.18(−0.39-0.76) |
| Western Sub-Saharan Africa | 1.85(1.33-2.45) | 110.60 | −0.04(−0.14-0.07) | 0.84(0.63-1.12) | | 128.41 | 0.20(0.12-0.28) |
| North Africa and Middle East | 2.57(1.65-3.91) | 165.52 | 0.50(0.34-0.66) | 2.72(1.89-3.93) | | 226.93 | 1.11(0.96-1.26) |
| Central Sub-Saharan Africa | 0.18(0.12-0.27) | 116.44 | −0.46(−0.54-−0.39) | 0.09(0.06-0.13) | | 143.74 | −0.18(−0.26-−0.10) |

LCAL, liver cancer due to alcohol use; LCNA, liver cancer due to non-alcoholic steatohepatitis; EAPC: estimated annual percentage change; CI, confidence interval; SDI: socio-demographic index.

**Supplementary table 5.** The percentage change in number and the EAPCs of death due to liver cancer caused by specific etiologies at national level from 1990 to 2019.

|  | **LCAL** | | | | **LCNA** | | | |
| --- | --- | --- | --- | --- | --- | --- | --- | --- |
| **Characteristics** | ASDR/100,000  in 2019 | Changes in number (%) | EAPC  (95%CI) | | ASDR/100,000  in 2019 | Changes in number (%) | EAPC  (95%CI) | |
| Afghanistan | 0.88(0.53-1.33) | 45.09 | −0.55(−0.59-−0.51) | 0.90(0.57-1.34) | | 81.11 | | 0.05(0.01-0.08) |
| Albania | 2.53(1.60-3.68) | 62.87 | −1.82(−2.29-−1.36) | | 0.56(0.35-0.86) | 45.44 | −2.72(−3.32-−2.11) | |
| Algeria | 0.23(0.13-0.35) | 271.32 | 1.13(1.01-1.26) | | 0.25(0.16-0.37) | 339.92 | 1.66(1.57-1.74) | |
| American Samoa | 0.78(0.49-1.17) | 167.97 | 1.21(1.02-1.39) | | 0.84(0.59-1.20) | 201.21 | 1.28(0.91-1.65) | |
| Andorra | 3.73(2.45-5.15) | 137.31 | −0.18(−0.21-−0.14) | | 0.65(0.40-1.01) | 236.18 | 0.73(0.62-0.84) | |
| Angola | 0.44(0.28-0.65) | 235.75 | 0.66(0.58-0.73) | | 0.18(0.12-0.27) | 229.26 | 0.29(0.21-0.37) | |
| Antigua and Barbuda | 1.00(0.72-1.35) | −28.80 | −3.43(−4.42-−2.42) | | 0.28(0.19-0.40) | −24.59 | −3.10(−4.06-−2.12) | |
| Argentina | 0.70(0.50-0.91) | 131.64 | 1.88(1.59-2.18) | | 0.20(0.14-0.28) | 207.87 | 2.74(2.51-2.98) | |
| Armenia | 2.06(1.44-2.80) | 1137.65 | 10.45(8.85-12.08) | | 0.57(0.39-0.83) | 1343.80 | 10.87(9.25-12.51) | |
| Australia | 1.62(1.22-2.01) | 303.20 | 2.78(2.53-3.02) | | 0.45(0.32-0.62) | 555.71 | 4.19(3.89-4.49) | |
| Austria | 2.40(1.94-2.90) | 106.76 | 1.33(1.08-1.57) | | 0.22(0.15-0.33) | 139.86 | 1.94(1.63-2.26) | |
| Azerbaijan | 1.16(0.72-1.76) | 802.78 | 6.70(4.72-8.72) | | 0.37(0.23-0.60) | 862.15 | 7.29(5.34-9.27) | |
| Bahamas | 1.09(0.75-1.49) | −13.16 | −3.88(−4.81-−2.95) | | 0.32(0.22-0.47) | 20.34 | −2.73(−3.52-−1.92) | |
| Bahrain | 0.52(0.31-0.81) | 297.80 | −1.88(−2.25-−1.51) | | 0.77(0.51-1.16) | 445.64 | −0.25(−0.63-0.14) | |
| Bangladesh | 0.58(0.35-0.84) | 124.70 | −1.09(−1.27-−0.92) | | 0.22(0.14-0.32) | 147.16 | −0.28(−0.41-−0.14) | |
| Barbados | 1.00(0.70-1.33) | 74.19 | −0.05(−0.39-0.29) | | 0.29(0.20-0.43) | 75.05 | 0.26(0.07-0.44) | |
| Belarus | 1.01(0.66-1.48) | 166.62 | 3.72(2.91-4.54) | | 0.18(0.11-0.28) | 148.51 | 3.11(2.64-3.59) | |
| Belgium | 1.47(1.10-1.87) | 98.47 | 1.12(0.80-1.45) | | 0.24(0.16-0.35) | 108.00 | 0.89(0.50-1.28) | |
| Belize | 1.13(0.82-1.48) | 64.55 | −2.01(−2.60-−1.40) | | 0.32(0.22-0.46) | 63.19 | −2.01(−2.48-−1.54) | |
| Benin | 1.00(0.62-1.55) | 89.66 | −1.12(−1.30-−0.94) | | 0.53(0.35-0.80) | 134.44 | −0.21(−0.31-−0.10) | |
| Bermuda | 0.79(0.57-1.06) | −41.54 | −4.38(−5.45-−3.29) | | 0.22(0.15-0.32) | −30.24 | −4.08(−5.08-−3.06) | |
| Bhutan | 0.94(0.54-1.48) | 212.36 | 1.18(1.09-1.27) | | 0.32(0.19-0.51) | 313.09 | 2.37(2.23-2.51) | |
| Bolivia | 1.57(1.02-2.27) | 195.96 | 0.23(0.09-0.36) | | 0.53(0.34-0.83) | 224.15 | 0.46(0.38-0.54) | |
| Bosnia and Herzegovina | 2.73(1.86-3.77) | 117.96 | 1.75(1.48-2.02) | | 0.78(0.52-1.15) | 205.53 | 3.07(2.59-3.55) | |
| Botswana | 0.32(0.2-0.5) | 333.18 | 0.98(−0.01-1.98) | | 0.16(0.1-0.24) | 328.15 | 1.23(0.59-1.89) | |
| Brazil | 0.76(0.65-0.86) | 256.99 | 1.46(1.32-1.61) | | 0.16(0.14-0.19) | 270.27 | 1.57(1.36-1.78) | |
| Brunei Darussalam | 1.06(0.65-1.57) | 260.97 | 1.17(0.66-1.68) | | 0.71(0.47-1.04) | 310.14 | 1.38(0.88-1.89) | |
| Bulgaria | 2.08(1.49-2.81) | −11.68 | −0.09(−0.66-0.48) | | 0.35(0.23-0.5) | −11.57 | −0.52(−1.04-0) | |
| Burkina Faso | 0.4(0.25-0.58) | 70.16 | −0.91(−1.12-−0.7) | | 0.21(0.14-0.31) | 105.17 | −0.32(−0.51-−0.13) | |
| Burundi | 0.89(0.51-1.58) | 30.52 | −1.88(−2.11-−1.65) | | 0.37(0.23-0.6) | 61.51 | −0.9(−1.07-−0.73) | |
| Cabo Verde | 2.4(1.62-3.32) | 2060.08 | 6.71(4.5-8.97) | | 1.41(0.96-2.09) | 2165.84 | 7.47(5.28-9.72) | |
| Cambodia | 1.61(1.03-2.32) | 241.15 | 0.61(0.44-0.78) | | 0.66(0.43-0.97) | 150.36 | −0.16(−0.28-−0.04) | |
| Cameroon | 0.15(0.09-0.23) | 205.34 | 0.3(0.1-0.51) | | 0.07(0.05-0.11) | 209.64 | 0.26(0.07-0.45) | |
| Canada | 2.17(1.75-2.56) | 322.60 | 2.78(2.56-3) | | 0.5(0.36-0.7) | 435.67 | 3.53(3.32-3.75) | |
| Central African  Republic | 0.54(0.3-0.92) | 58.37 | −1.06(−1.31-−0.82) | | 0.24(0.14-0.39) | 81.91 | −0.41(−0.63-−0.2) | |
| Chad | 0.92(0.56-1.41) | 76.93 | −0.38(−0.49-−0.28) | | 0.48(0.31-0.72) | 85.77 | −0.21(−0.29-−0.12) | |
| Chile | 0.94(0.68-1.22) | 233.08 | 1.75(1.42-2.08) | | 0.32(0.22-0.45) | 346.52 | 2.62(2.39-2.84) | |
| China | 0.85(0.63-1.09) | −0.51 | −4.46(−5.28-−3.63) | | 0.48(0.39-0.59) | 6.03 | −4.2(−5.01-−3.39) | |
| Colombia | 0.84(0.56-1.21) | 196.78 | 0.08(−0.42-0.57) | | 0.21(0.13-0.32) | 252.97 | 0.57(0.11-1.02) | |
| Comoros | 0.61(0.34-1.16) | 92.19 | −0.49(−0.65-−0.34) | | 0.46(0.28-0.74) | 129.69 | −0.04(−0.18-0.09) | |
| Congo | 0.52(0.32-0.79) | 88.07 | −1.12(−1.3-−0.94) | | 0.26(0.16-0.39) | 112.37 | −0.74(−0.9-−0.59) | |
| Cook Islands | 1.82(1.17-2.63) | 123.32 | 0.73(0.61-0.86) | | 1.34(0.91-1.87) | 113.40 | 0.08(−0.05-0.2) | |
| Costa Rica | 1.82(1.24-2.58) | 179.99 | −0.4(−0.97-0.16) | | 0.48(0.31-0.72) | 246.06 | 0.34(−0.18-0.86) | |
| Croatia | 1.66(1.17-2.22) | 64.41 | 1(0.43-1.57) | | 0.34(0.22-0.5) | 75.06 | 0.85(0.31-1.39) | |
| Cuba | 0.9(0.62-1.23) | −23.57 | −3.21(−4.45-−1.96) | | 0.25(0.16-0.38) | −20.54 | −3.31(−4.55-−2.05) | |
| Cyprus | 1.24(0.9-1.61) | 149.36 | 0.31(0.14-0.48) | | 0.23(0.15-0.33) | 191.35 | 0.79(0.51-1.07) | |
| Czechia | 1.44(1.07-1.88) | 7.15 | −1.47(−1.66-−1.28) | | 0.22(0.15-0.33) | 18.31 | −1.2(−1.41-−1) | |
| Côte d'Ivoire | 1.08(0.67-1.67) | 80.78 | −2.16(−2.52-−1.8) | | 0.49(0.32-0.76) | 120.78 | −1.23(−1.48-−0.99) | |
| Democratic People's Republic of Korea | 0.83(0.48-1.31) | 49.39 | −1.12(−1.29-−0.95) | | 0.51(0.31-0.79) | 46.59 | −1.39(−1.51-−1.28) | |
| Democratic Republic of the Congo | 0.29(0.18-0.44) | 96.65 | −0.76(−0.87-−0.66) | | 0.16(0.1-0.24) | 132.14 | −0.25(−0.32-−0.18) | |
| Denmark | 1.28(0.95-1.63) | 134.00 | 2.11(1.87-2.35) | | 0.2(0.14-0.29) | 166.07 | 2.62(2.36-2.87) | |
| Djibouti | 0.84(0.46-1.62) | 345.95 | 0.15(0.01-0.29) | | 0.48(0.28-0.8) | 391.47 | 0.52(0.41-0.63) | |
| Dominica | 1.22(0.84-1.7) | −49.66 | −3.45(−4.27-−2.63) | | 0.34(0.23-0.49) | −48.08 | −3.11(−3.86-−2.36) | |
| Dominican Republic | 1.73(1.04-2.87) | 220.73 | 1.01(0.53-1.48) | | 0.45(0.28-0.7) | 265.11 | 1.69(1.17-2.22) | |
| Ecuador | 1.11(0.75-1.59) | 309.71 | 1.78(1.42-2.14) | | 0.49(0.32-0.71) | 322.02 | 1.75(1.42-2.09) | |
| Egypt | 1.8(1-3.09) | 212.83 | 2.08(1.7-2.46) | | 1.96(1.18-3.09) | 279.46 | 2.65(2.33-2.97) | |
| El Salvador | 0.67(0.43-0.96) | 48.33 | −1.33(−1.89-−0.76) | | 0.19(0.12-0.3) | 52.45 | −1.14(−1.82-−0.46) | |
| Equatorial Guinea | 0.62(0.32-1.02) | 223.19 | 1.36(1.15-1.56) | | 0.32(0.17-0.52) | 276.37 | 1.73(1.61-1.86) | |
| Eritrea | 0.74(0.42-1.24) | 155.82 | −0.48(−0.69-−0.27) | | 0.45(0.27-0.71) | 194.05 | 0.12(−0.07-0.31) | |
| Estonia | 1.6(1.13-2.12) | 168.85 | 1.95(1.37-2.53) | | 0.3(0.19-0.45) | 160.21 | 1.46(0.94-1.98) | |
| Eswatini | 4.58(1.14-9.27) | 748.70 | 6.06(4.6-7.55) | | 1.87(0.62-3.44) | 552.34 | 4.79(3.54-6.06) | |
| Ethiopia | 0.69(0.51-0.93) | 96.48 | −0.18(−0.31-−0.05) | | 0.31(0.24-0.41) | 116.76 | −0.02(−0.13-0.09) | |
| Fiji | 0.84(0.51-1.31) | 147.21 | 1.05(0.69-1.41) | | 0.67(0.43-0.98) | 168.78 | 1.22(0.99-1.45) | |
| Finland | 1.54(1.14-1.94) | 158.00 | 1.88(1.68-2.08) | | 0.27(0.18-0.4) | 178.45 | 1.81(1.66-1.95) | |
| France | 1.97(1.4-2.61) | 69.63 | −0.21(−0.44-0.03) | | 0.36(0.24-0.53) | 150.86 | 1.08(0.8-1.36) | |
| Gabon | 0.64(0.34-1.1) | 92.90 | 0.04(−0.11-0.19) | | 0.3(0.17-0.47) | 138.19 | 0.69(0.49-0.88) | |
| Gambia | 7.7(4.5-11.55) | 281.28 | 0.98(0.74-1.21) | | 3.43(2.23-5.16) | 333.39 | 1.23(1.01-1.46) | |
| Georgia | 1.22(0.85-1.63) | 277.89 | 4.12(2.95-5.3) | | 0.26(0.18-0.37) | 206.98 | 2.96(1.89-4.04) | |
| Germany | 1.88(1.53-2.24) | 166.95 | 2.5(2.16-2.84) | | 0.33(0.23-0.46) | 192.93 | 2.57(2.1-3.03) | |
| Ghana | 1.36(0.89-1.98) | 186.94 | 0.13(−0.04-0.3) | | 0.51(0.34-0.76) | 212.27 | 0.33(0.11-0.56) | |
| Greece | 1.1(0.82-1.41) | 122.11 | 1.5(1.31-1.68) | | 0.24(0.16-0.34) | 156.62 | 1.46(1.33-1.58) | |
| Greenland | 2.68(1.86-3.67) | 174.96 | 1.51(1.4-1.63) | | 0.67(0.44-0.95) | 212.20 | 1.31(1.13-1.49) | |
| Grenada | 1.19(0.88-1.54) | −41.99 | −3.81(−4.68-−2.92) | | 0.29(0.19-0.41) | −44.75 | −3.36(−4.31-−2.4) | |
| Guam | 0.73(0.45-1.1) | 307.29 | 2.11(1.88-2.35) | | 0.57(0.39-0.84) | 333.79 | 1.83(1.64-2.02) | |
| Guatemala | 1.2(0.78-1.73) | 52.72 | −3.41(−4.77-−2.03) | | 0.43(0.28-0.64) | 93.73 | −2.65(−4.14-−1.14) | |
| Guinea | 6.14(3.54-9.17) | 94.48 | 0.77(0.72-0.82) | | 2.75(1.73-4.23) | 101.26 | 0.68(0.63-0.73) | |
| Guinea-Bissau | 1.15(0.71-1.75) | 37.08 | −1.03(−1.11-−0.94) | | 0.61(0.37-0.94) | 68.83 | −0.24(−0.31-−0.18) | |
| Guyana | 1.11(0.76-1.54) | −25.31 | −2.83(−3.68-−1.96) | | 0.3(0.2-0.44) | −7.43 | −1.98(−2.77-−1.18) | |
| Haiti | 1.47(0.8-2.5) | 36.58 | −1.66(−1.87-−1.45) | | 0.32(0.17-0.52) | 40.34 | −1.51(−1.74-−1.27) | |
| Honduras | 4.55(1.87-7.31) | 323.29 | 1.7(1.47-1.93) | | 1.38(0.52-2.42) | 396.73 | 2.29(2.09-2.48) | |
| Hungary | 1.12(0.8-1.48) | −51.16 | −2.64(−3.45-−1.82) | | 0.22(0.15-0.31) | −38.4 | −2.16(−2.94-−1.37) | |
| Iceland | 1.04(0.76-1.35) | 231.67 | 2.04(1.91-2.17) | | 0.2(0.13-0.3) | 213.85 | 1.67(1.51-1.84) | |
| India | 0.76(0.61-0.94) | 194.97 | 0.31(0.23-0.4) | | 0.33(0.26-0.41) | 209.59 | 0.34(0.23-0.45) | |
| Indonesia | 0.43(0.34-0.53) | 110.46 | −0.14(−0.22-−0.06) | | 0.25(0.2-0.29) | 117.07 | 0.17(0.11-0.22) | |
| Iran  (Islamic Republic of) | 0.4(0.3-0.52) | 129.44 | −1.14(−1.73-−0.54) | | 0.54(0.45-0.64) | 197.76 | −0.65(−1.31-0.01) | |
| Iraq | 0.6(0.37-0.91) | 297.6 | 1.31(0.86-1.76) | | 0.69(0.45-1.01) | 338.73 | 1.84(1.36-2.33) | |
| Ireland | 1.26(0.93-1.61) | 338.97 | 3.62(3.26-3.98) | | 0.25(0.17-0.37) | 388.17 | 4.18(3.82-4.53) | |
| Israel | 0.73(0.51-0.97) | 177.86 | 0.63(0.55-0.72) | | 0.23(0.16-0.33) | 172.22 | 0.3(0.17-0.43) | |
| Italy | 1.25(1.05-1.45) | 8.00 | −1.61(−1.89-−1.34) | | 0.22(0.18-0.27) | 26.27 | −1.47(−1.75-−1.19) | |
| Jamaica | 0.93(0.64-1.28) | 14.57 | −0.56(−1.47-0.37) | | 0.28(0.18-0.42) | 36.80 | 0.13(−0.78-1.04) | |
| Japan | 0.99(0.83-1.16) | 37.44 | −2.25(−2.75-−1.75) | | 0.4(0.32-0.48) | 104.77 | −1.36(−1.95-−0.77) | |
| Jordan | 0.27(0.16-0.42) | 318.65 | −0.62(−0.74-−0.51) | | 0.32(0.21-0.48) | 360.36 | −0.34(−0.48-−0.19) | |
| Kazakhstan | 2.07(1.49-2.72) | 157.39 | −1.37(−2.54-−0.19) | | 0.52(0.35-0.77) | 222.87 | 0.19(−0.79-1.18) | |
| Kenya | 0.86(0.54-1.33) | 225.44 | 0.02(−0.43-0.47) | | 0.53(0.36-0.77) | 265.26 | 0.78(0.49-1.07) | |
| Kiribati | 1.21(0.72-1.78) | 58.90 | −0.63(−0.69-−0.56) | | 1.17(0.77-1.76) | 87.43 | 0.01(−0.13-0.16) | |
| Kuwait | 0.26(0.15-0.4) | 242.67 | 0.44(0.11-0.78) | | 0.35(0.23-0.53) | 340.66 | 1.43(1.07-1.8) | |
| Kyrgyzstan | 0.81(0.57-1.08) | 249.70 | 4(3.5-4.51) | | 0.18(0.12-0.26) | 227.54 | 3.71(3.4-4.01) | |
| Lao People's Democratic Republic | 1.69(1.1-2.51) | 62.09 | −1.08(−1.25-−0.91) | | 0.58(0.37-0.85) | 59.80 | −1.09(−1.22-−0.96) | |
| Latvia | 1.16(0.84-1.52) | 111.69 | 1.52(0.75-2.3) | | 0.22(0.15-0.33) | 104.02 | 1.21(0.5-1.94) | |
| Lebanon | 0.38(0.22-0.63) | 98.25 | −0.53(−0.64-−0.43) | | 0.29(0.18-0.47) | 166.59 | 0.17(0.05-0.28) | |
| Lesotho | 3.56(1.22-6.01) | 363.57 | 4.77(3.94-5.61) | | 1.4(0.62-2.34) | 296.53 | 4.38(3.78-4.98) | |
| Liberia | 1.01(0.61-1.65) | 28.55 | −1.25(−1.41-−1.08) | | 0.55(0.35-0.85) | 70.31 | −0.17(−0.35-0) | |
| Libya | 0.5(0.28-0.8) | 171.35 | 0.06(−0.07-0.19) | | 0.69(0.44-1.06) | 211.01 | 0.63(0.45-0.8) | |
| Lithuania | 1.36(0.96-1.78) | 186.61 | 2.45(2.1-2.8) | | 0.22(0.14-0.32) | 143.67 | 1.48(1.13-1.83) | |
| Luxembourg | 1.41(1-1.91) | 109.66 | 0.66(0.44-0.88) | | 0.24(0.15-0.35) | 157.21 | 1.2(0.94-1.46) | |
| Madagascar | 0.63(0.34-1.15) | 73.29 | −0.78(−0.94-−0.62) | | 0.34(0.2-0.54) | 94.80 | −0.24(−0.35-−0.13) | |
| Malawi | 0.76(0.5-1.08) | 76.74 | −1.09(−1.55-−0.63) | | 0.4(0.27-0.58) | 82.51 | −0.67(−1-−0.34) | |
| Malaysia | 0.85(0.52-1.31) | 233.50 | 0.5(0.19-0.8) | | 0.73(0.48-1.08) | 313.43 | 1.42(1.04-1.8) | |
| Maldives | 1.26(0.82-1.83) | 187.68 | −0.61(−0.72-−0.49) | | 0.56(0.37-0.86) | 284.8 | 0.34(0.17-0.5) | |
| Mali | 2.59(1.59-3.9) | 114.30 | 0.13(0.02-0.24) | | 1.06(0.68-1.59) | 142.2 | 0.41(0.33-0.49) | |
| Malta | 0.78(0.56-1.02) | 218.45 | 1.48(1.25-1.7) | | 0.16(0.11-0.23) | 245.64 | 1.52(1.15-1.88) | |
| Marshall Islands | 1.35(0.8-2.18) | 99.11 | −0.12(−0.25-0) | | 0.92(0.59-1.4) | 109.62 | 0.01(−0.09-0.12) | |
| Mauritania | 0.67(0.4-1.03) | 27.03 | −1.52(−1.67-−1.36) | | 0.55(0.35-0.81) | 63.45 | −0.79(−0.88-−0.71) | |
| Mauritius | 0.47(0.3-0.7) | 179.71 | 1.87(1.49-2.25) | | 0.24(0.16-0.36) | 224.22 | 2.35(1.95-2.76) | |
| Mexico | 1.11(0.89-1.36) | 387.57 | 1.93(1.79-2.08) | | 0.38(0.31-0.47) | 421.74 | 2.3(2.15-2.46) | |
| Micronesia  (Federated States of) | 1.34(0.75-2.1) | 45.4 | −0.24(−0.38-−0.09) | | 1.08(0.65-1.67) | 64.75 | 0.19(0.09-0.29) | |
| Monaco | 2.68(1.84-3.71) | 224.28 | 3.77(2.94-4.61) | | 0.63(0.4-0.96) | 257.35 | 4.13(3.26-5.01) | |
| Mongolia | 34.2(23.11-47.83) | 298.49 | 3(2.6-3.41) | | 8.72(5.64-12.87) | 343.38 | 4.23(3.6-4.86) | |
| Montenegro | 2.53(1.8-3.42) | 54.92 | −0.2(−0.34-−0.06) | | 0.52(0.35-0.75) | 87.50 | 0.44(0.25-0.64) | |
| Morocco | 0.26(0.16-0.41) | 169.08 | 0.42(0.08-0.75) | | 0.28(0.18-0.41) | 225.36 | 1.11(0.84-1.38) | |
| Mozambique | 0.99(0.57-1.45) | 278.85 | 2.49(2.25-2.73) | | 0.47(0.31-0.7) | 230.38 | 1.9(1.72-2.09) | |
| Myanmar | 0.89(0.59-1.27) | 235.88 | 1.74(1.56-1.92) | | 0.48(0.33-0.71) | 264.87 | 2.01(1.8-2.22) | |
| Namibia | 0.84(0.54-1.2) | 331.17 | 2.92(2.43-3.41) | | 0.32(0.21-0.47) | 257.13 | 2.01(1.63-2.38) | |
| Nauru | 1.24(0.71-1.9) | 5.14 | −0.3(−0.6-0) | | 0.94(0.6-1.45) | 13.76 | −0.25(−0.49-−0.02) | |
| Nepal | 0.64(0.38-1.04) | 210.34 | 0.97(0.81-1.12) | | 0.24(0.15-0.37) | 257.28 | 1.44(1.26-1.63) | |
| Netherlands | 1.11(0.86-1.36) | 240.09 | 2.69(2.54-2.85) | | 0.23(0.16-0.32) | 295.55 | 3.26(3.09-3.43) | |
| New Zealand | 1.41(1.23-1.6) | 215.81 | 1.92(1.73-2.1) | | 0.4(0.34-0.47) | 370.73 | 3.12(2.87-3.36) | |
| Nicaragua | 1.26(0.85-1.75) | 289.05 | 1.32(0.92-1.72) | | 0.35(0.24-0.51) | 341.85 | 1.94(1.65-2.22) | |
| Niger | 0.11(0.06-0.16) | 160.85 | −0.35(−0.45-−0.25) | | 0.05(0.03-0.08) | 198.93 | 0.08(−0.01-0.16) | |
| Nigeria | 0.81(0.61-1.07) | 108.50 | 0.55(0.46-0.63) | | 0.38(0.3-0.49) | 118.24 | 0.57(0.51-0.62) | |
| Niue | 1.03(0.65-1.54) | 2.11 | −0.13(−0.22-−0.04) | | 0.81(0.52-1.19) | 6.59 | 0.3(0.18-0.42) | |
| North Macedonia | 3.2(2.15-4.47) | 71.33 | −0.27(−0.39-−0.16) | | 0.79(0.52-1.16) | 107.00 | 0.6(0.49-0.71) | |
| Northern Mariana Islands | 0.98(0.63-1.43) | 283.58 | 1.24(1.02-1.46) | | 0.8(0.54-1.15) | 235.61 | 0.4(0.3-0.51) | |
| Norway | 0.82(0.69-0.97) | 141.03 | 2.41(2.21-2.61) | | 0.17(0.14-0.2) | 143.09 | 2.41(2.24-2.57) | |
| Oman | 0.52(0.33-0.81) | 206.15 | 1.46(0.97-1.95) | | 0.59(0.4-0.87) | 335.32 | 2.92(2.51-3.34) | |
| Pakistan | 0.54(0.41-0.72) | 99.33 | 0.07(−0.11-0.24) | | 0.29(0.22-0.38) | 120.47 | 0.46(0.35-0.57) | |
| Palau | 1.36(0.85-2.04) | 139.12 | 0.24(0.16-0.33) | | 0.88(0.56-1.3) | 178.47 | 0.82(0.62-1.02) | |
| Palestine | 0.62(0.38-0.93) | 106.68 | −0.82(−0.91-−0.72) | | 0.7(0.48-1.02) | 108.83 | −0.69(−0.76-−0.63) | |
| Panama | 1.06(0.71-1.54) | 175.77 | 0.37(−0.12-0.85) | | 0.25(0.16-0.38) | 210.92 | 0.69(0.29-1.08) | |
| Papua New Guinea | 0.23(0.15-0.37) | 190.24 | 0.82(0.75-0.88) | | 0.13(0.08-0.2) | 210.10 | 0.84(0.78-0.89) | |
| Paraguay | 0.74(0.47-1.07) | 116.97 | −0.48(−1.07-0.11) | | 0.12(0.08-0.19) | 144.80 | 0.19(−0.37-0.76) | |
| Peru | 0.95(0.61-1.35) | 30.46 | −3.3(−3.96-−2.65) | | 0.27(0.17-0.4) | 60.99 | −2.83(−3.39-−2.26) | |
| Philippines | 1.57(1.18-2.03) | 89.07 | −1.53(−1.82-−1.23) | | 0.55(0.43-0.68) | 103.88 | −1.18(−1.48-−0.87) | |
| Poland | 0.91(0.74-1.13) | −44.28 | −3.38(−4.67-−2.06) | | 0.17(0.14-0.22) | −53.18 | −4.38(−5.73-−3.01) | |
| Portugal | 1.83(1.36-2.3) | 305.27 | 2.98(2.62-3.34) | | 0.28(0.19-0.4) | 359.17 | 2.99(2.6-3.38) | |
| Puerto Rico | 0.95(0.62-1.36) | −24.92 | −2.24(−2.98-−1.5) | | 0.36(0.23-0.54) | 1.83 | −1.29(−2.08-−0.48) | |
| Qatar | 1.8(1.02-2.9) | 592.31 | 0.07(−0.15-0.28) | | 2.53(1.56-3.92) | 664.98 | 0.73(0.54-0.93) | |
| Republic of Korea | 2.88(2-4.03) | 491.67 | 2.84(1.63-4.06) | | 0.94(0.64-1.37) | 461.61 | 2.24(1.11-3.37) | |
| Republic of Moldova | 1.16(0.92-1.45) | 79.15 | −1.45(−2.98-0.11) | | 0.16(0.11-0.23) | 161.69 | 0.57(−0.79-1.94) | |
| Romania | 1.37(0.98-1.79) | 142.13 | 3.01(2.56-3.47) | | 0.23(0.16-0.34) | 174.98 | 3.04(2.61-3.46) | |
| Russian Federation | 1.09(0.87-1.37) | 145.02 | 2.66(2.38-2.93) | | 0.25(0.21-0.31) | 170.01 | 3.01(2.68-3.35) | |
| Rwanda | 1.46(1-2.08) | 76.77 | −1.35(−1.68-−1.02) | | 0.58(0.38-0.82) | 102.82 | −0.53(−0.77-−0.3) | |
| Saint Kitts and Nevis | 1.32(0.93-1.8) | −47.69 | −4.42(−5.47-−3.36) | | 0.4(0.28-0.58) | −48.63 | −3.77(−4.75-−2.79) | |
| Saint Lucia | 0.9(0.66-1.17) | −7.61 | −3.57(−4.57-−2.56) | | 0.21(0.15-0.3) | 4.74 | −3.36(−4.4-−2.3) | |
| Saint Vincent and the Grenadines | 1.19(0.88-1.52) | −9.44 | −2.51(−3.44-−1.57) | | 0.29(0.2-0.41) | −12.47 | −2.32(−3.23-−1.41) | |
| Samoa | 0.67(0.41-1) | 35.36 | −0.71(−0.78-−0.64) | | 0.46(0.3-0.68) | 54.34 | −0.5(−0.58-−0.43) | |
| San Marino | 1.05(0.6-1.63) | 154.07 | 1.59(1.38-1.81) | | 0.19(0.11-0.32) | 194.21 | 1.93(1.69-2.18) | |
| Sao Tome and Principe | 0.61(0.35-0.92) | 116.99 | 0.96(0.82-1.09) | | 0.29(0.18-0.44) | 104.97 | 0.81(0.7-0.92) | |
| Saudi Arabia | 0.42(0.24-0.67) | 132.72 | −0.7(−0.96-−0.43) | | 0.76(0.49-1.15) | 219.23 | 0.41(0.07-0.74) | |
| Senegal | 0.3(0.18-0.45) | 111.94 | −0.01(−0.19-0.17) | | 0.29(0.19-0.43) | 156.72 | 0.43(0.3-0.55) | |
| Serbia | 2.03(1.37-2.79) | 33.21 | −0.49(−0.75-−0.23) | | 0.4(0.26-0.58) | 60.14 | −0.04(−0.27-0.18) | |
| Seychelles | 1.45(0.97-2.02) | 71.26 | −0.33(−0.56-−0.11) | | 0.63(0.43-0.89) | 54.10 | −0.41(−0.68-−0.14) | |
| Sierra Leone | 0.93(0.58-1.4) | 43.92 | −0.95(−1.1-−0.81) | | 0.47(0.3-0.71) | 82.88 | −0.01(−0.08-0.06) | |
| Singapore | 0.69(0.43-1.04) | 300.76 | 0.35(0.01-0.69) | | 0.44(0.28-0.67) | 395.95 | 1.19(0.89-1.5) | |
| Slovakia | 1.52(1.05-2.05) | 15.34 | −1.55(−1.82-−1.28) | | 0.26(0.17-0.4) | 36.05 | −1.07(−1.43-−0.71) | |
| Slovenia | 2.06(1.39-2.91) | 206.96 | 2.26(1.96-2.56) | | 0.45(0.28-0.7) | 278.66 | 2.88(2.53-3.22) | |
| Solomon Islands | 0.66(0.4-0.98) | 88.12 | −0.44(−0.52-−0.36) | | 0.5(0.34-0.72) | 113.62 | −0.1(−0.28-0.08) | |
| Somalia | 0.74(0.4-1.43) | 139.14 | −0.3(−0.4-−0.2) | | 0.45(0.26-0.78) | 164.88 | 0.21(0.12-0.3) | |
| South Africa | 1.23(1-1.47) | 111.77 | −0.61(−1.31-0.09) | | 0.73(0.61-0.87) | 136.15 | 0.06(−0.53-0.66) | |
| South Sudan | 0.68(0.33-1.41) | 35.16 | −0.34(−0.41-−0.28) | | 0.4(0.22-0.69) | 54.48 | 0.1(0.06-0.15) | |
| Spain | 1.45(1-1.93) | 96.64 | 0.33(0.03-0.63) | | 0.29(0.19-0.42) | 170.98 | 1.17(0.79-1.55) | |
| Sri Lanka | 0.7(0.43-1.05) | 296.54 | 2.64(2.26-3.01) | | 0.29(0.18-0.45) | 258.08 | 2.19(1.89-2.49) | |
| Sudan | 0.57(0.3-1.07) | 113.26 | 0.46(0.31-0.61) | | 0.54(0.31-0.86) | 164.93 | 1.19(0.98-1.41) | |
| Suriname | 0.96(0.68-1.34) | −1.28 | −2.99(−3.77-−2.21) | | 0.27(0.18-0.39) | 14.11 | −2.31(−3.13-−1.49) | |
| Sweden | 1.35(1.16-1.53) | 81.33 | 1.61(1.04-2.18) | | 0.23(0.19-0.28) | 70.57 | 1.24(0.66-1.82) | |
| Switzerland | 1.8(1.32-2.29) | 187.02 | 1.41(0.9-1.92) | | 0.26(0.17-0.38) | 262.04 | 2.31(1.8-2.81) | |
| Syrian Arab Republic | 0.41(0.24-0.65) | 104.03 | −0.7(−0.85-−0.55) | | 0.55(0.35-0.83) | 137.58 | −0.05(−0.22-0.13) | |
| Taiwan  (Province of China) | 0.66(0.4-1.02) | 29.89 | −3.24(−4.15-−2.32) | | 0.58(0.38-0.87) | 130.47 | −1.09(−1.96-−0.21) | |
| Tajikistan | 0.89(0.57-1.3) | 550.42 | 5.62(5.37-5.87) | | 0.24(0.15-0.36) | 612.34 | 6.21(5.87-6.56) | |
| Thailand | 7.17(4.61-10.45) | 285.06 | 1.2(1.03-1.37) | | 2.41(1.52-3.6) | 352.71 | 1.62(1.46-1.78) | |
| Timor−Leste | 1.33(0.72-2.16) | 182.55 | −0.5(−0.69-−0.32) | | 0.57(0.34-0.85) | 175.31 | −0.39(−0.58-−0.2) | |
| Togo | 0.94(0.6-1.39) | 136.10 | −1.34(−1.55-−1.12) | | 0.53(0.35-0.81) | 179.68 | −0.48(−0.63-−0.33) | |
| Tokelau | 0.88(0.51-1.35) | −9.41 | −0.32(−0.36-−0.27) | | 0.76(0.49-1.12) | −0.33 | 0(−0.08-0.07) | |
| Tonga | 3.31(1.96-5.14) | 58.97 | 0.43(0.19-0.66) | | 2.46(1.56-3.62) | 86.62 | 0.54(0.16-0.92) | |
| Trinidad and Tobago | 0.92(0.6-1.32) | −10.81 | −3.34(−4.36-−2.31) | | 0.3(0.19-0.45) | 1.44 | −2.85(−3.89-−1.8) | |
| Tunisia | 0.21(0.11-0.38) | 153.11 | 0.02(−0.1-0.14) | | 0.14(0.08-0.22) | 197.04 | 0.73(0.61-0.85) | |
| Turkey | 0.39(0.24-0.6) | 106.68 | −0.72(−0.9-−0.53) | | 0.34(0.23-0.49) | 160.36 | 0.13(−0.17-0.42) | |
| Turkmenistan | 1.71(1.14-2.43) | 1338.65 | 7.41(6.36-8.47) | | 0.41(0.27-0.62) | 1197.26 | 6.93(6.02-7.85) | |
| Tuvalu | 1.06(0.63-1.66) | 28.58 | −0.51(−0.66-−0.37) | | 0.8(0.51-1.19) | 47.29 | −0.36(−0.47-−0.25) | |
| Uganda | 2.07(1.42-2.85) | 183.00 | 1.12(0.93-1.31) | | 0.76(0.5-1.12) | 254.63 | 1.89(1.68-2.09) | |
| Ukraine | 0.92(0.74-1.13) | 228.75 | 4.95(4.28-5.63) | | 0.21(0.17-0.26) | 190.22 | 4.12(3.69-4.54) | |
| United Arab Emirates | 0.61(0.2-1.63) | 911.33 | 0.13(−0.05-0.31) | | 0.64(0.25-1.62) | 1178.08 | 1.18(0.81-1.55) | |
| United Kingdom | 1.45(1.28-1.63) | 202.58 | 3.4(3.19-3.61) | | 0.3(0.25-0.35) | 259.83 | 3.88(3.68-4.08) | |
| United Republic of Tanzania | 0.68(0.44-0.98) | 158.13 | 0.48(0.31-0.65) | | 0.34(0.23-0.48) | 189.51 | 0.82(0.68-0.95) | |
| United States of America | 1.35(1.1-1.6) | 293.92 | 2.74(2.66-2.82) | | 0.45(0.37-0.54) | 286.45 | 2.96(2.76-3.16) | |
| United States Virgin Islands | 0.9(0.64-1.22) | 56.64 | −1.27(−1.88-−0.65) | | 0.33(0.23-0.47) | 74.43 | −1.12(−1.62-−0.62) | |
| Uruguay | 0.72(0.51-0.94) | 134.85 | 2.38(2.2-2.57) | | 0.23(0.16-0.33) | 183.72 | 2.78(2.62-2.94) | |
| Uzbekistan | 1.87(1.28-2.55) | 1486.05 | 10.06(8.97-11.17) | | 0.55(0.37-0.8) | 1463.99 | 10.39(9.44-11.34) | |
| Vanuatu | 1.22(0.66-2.01) | 178.40 | 0.28(0.15-0.41) | | 0.79(0.47-1.21) | 211.83 | 0.52(0.42-0.62) | |
| Venezuela | 0.85(0.57-1.21) | 14.40 | −2.68(−3.88-−1.47) | | 0.21(0.13-0.32) | 31.46 | −2.17(−3.43-−0.88) | |
| Viet Nam | 0.66(0.4-0.97) | 99.63 | −0.48(−0.67-−0.28) | | 0.27(0.17-0.4) | 70.46 | −0.98(−1.18-−0.78) | |
| Yemen | 0.24(0.13-0.4) | 196.41 | 0.52(0.43-0.61) | | 0.3(0.18-0.46) | 218.17 | 0.7(0.59-0.81) | |
| Zambia | 0.83(0.55-1.16) | 182.33 | −0.1(−0.53-0.33) | | 0.41(0.27-0.6) | 195.21 | 0.23(−0.08-0.54) | |
| Zimbabwe | 2.14(1.3-3.49) | 79.18 | −1.17(−1.9-−0.44) | | 1.31(0.84-2.02) | 134.14 | 0.16(−0.42-0.75) | |

LCHB, liver cancer due to hepatitis B; LCHC, liver cancer due to hepatitis C; LCAL, liver cancer due to alcohol use; LCNA, liver cancer due to non-alcoholic steatohepatitis; EAPC: estimated annual percentage change; CI, confidence interval.
